# A family of *cis*-macrocyclic diphosphines: modular, stereoselective synthesis and application in catalytic CO_2_/ethylene coupling†
†Electronic supplementary information (ESI) available: Full experimental, crystallographic and spectroscopic data. CCDC 1477899–1477902. For ESI and crystallographic data in CIF or other electronic format see DOI: 10.1039/c6sc03614g



**DOI:** 10.1039/c6sc03614g

**Published:** 2016-10-11

**Authors:** Ioana Knopf, Daniel Tofan, Dirk Beetstra, Abdulaziz Al-Nezari, Khalid Al-Bahily, Christopher C. Cummins

**Affiliations:** a Department of Chemistry , Massachusetts Institute of Technology , 77 Massachusetts Avenue , Cambridge , MA 02139-4307 , USA . Email: ccummins@mit.edu ; Tel: +1 617 253 5332; b SABIC CRD , Fundamental Catalysis , Thuwal 23955-6900 , Saudi Arabia

## Abstract

A family of *cis*-macrocyclic diphosphines was prepared in just three steps from white phosphorus and commercial materials using a modular synthetic approach. Alkylation of bicyclic diphosphane 3,4,8,9-tetramethyl-1,6-diphosphabicyclo(4.4.0)deca-3,8-diene, or P_2_(dmb)_2_, produced phosphino-phosphonium salts [R-P_2_(dmb)_2_]X, where R is methyl, benzyl and isobutyl, in yields of 90–96%. Treatment of these salts with organolithium or Grignard reagents yielded symmetric and unsymmetric macrocyclic diphosphines of the form *cis*-1-R-6-R′-3,4,8,9-tetramethyl-2,5,7,10-tetrahydro-1,6-DiPhospheCine, or R,R′-DPC, in which R′ is methyl, cyclohexyl, phenyl or mesityl, in yields of 46–94%. Alternatively, symmetric diphosphine Cy_2_-DPC was synthesized in 74% yield from the dichlorodiphosphine Cl_2_P_2_(dmb)_2_. As a first application, these *cis*-macrocyclic diphosphines were used as ligands in the nickel-catalyzed synthesis of acrylate from CO_2_ and ethylene, for which they showed promising catalytic activity.

## Abstract 2

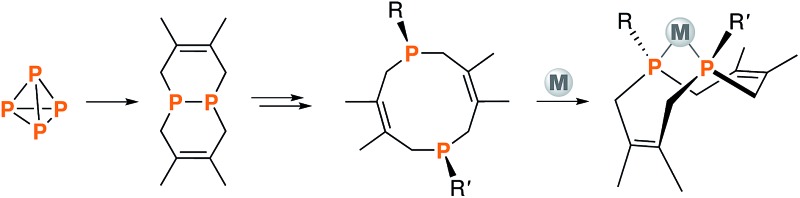
The stereoselective synthesis of a family of *cis*-macrocyclic diphosphines was achieved in only three steps from white phosphorus and commercial materials. These new ligands showed activity in the nickel-catalyzed coupling of CO_2_ and ethylene.

While chelating diphosphines have established a wide range of uses in areas from fundamental chemistry to catalysis,^1^ macrocyclic diphosphines are an underrepresented class of ligands. Their scarcity is primarily due to challenges associated with their synthesis. Macrocycles containing two or more phosphorus atoms exhibit multiple stereoisomers since the phosphines, which are locked in place by the cyclic framework, have a high barrier to inversion. Typically, syntheses of macrocyclic diphosphines yield mixtures of diastereomers;^2,3^ stereoselective syntheses of either *cis* or *trans* macrocyclic diphosphines are rare.^4,5^ While synthetically challenging, embedding one or more phosphorus atoms in a cyclic framework is desirable as it leads to more rigid and robust structures compared to their acyclic phosphine counterparts.^6^

*cis*-Macrocyclic diphosphines have been postulated to be “interesting ligands for transition metal complexes, comparable to, but perhaps usefully different from, the familiar range of chelating diphosphines”,^7^ yet their coordination chemistry is essentially unexplored.^8^ In this context, it is useful to distinguish between medium-sized (7–12-membered rings) and large (>12 membered rings) macrocyclic diphosphines. Complexes of large macrocyclic diphosphines have been synthesized as mixtures of diastereomers *via* ring closing metathesis and hydrogenation of preassembled metal complexes of *trans*-spanning monophosphines with pendant olefins.^3^ This synthetic strategy was also extended to prepare metal complexes of large *trans*-spanning macrobicyclic diphosphines.^9^ While both *cis* and *trans* isomers of a diphosphine embedded in a very large macrocycle can bind to a metal center, only the *cis* isomer can bind to a single metal center in medium-sized ring systems. The only medium-sized macrocyclic diphosphine with structurally characterized metal complexes is *cis*-1,5-diphenyl-1,5-diphosphacyclooctane, a ligand obtained after tedious separation of the *cis* and *trans* diastereomers produced by the lithium aluminum hydride reduction of the corresponding phosphine oxides.^10^

The only stereoselective synthetic route to *cis* medium-sized macrocyclic diphosphines was reported by Alder *et al.* ^5^ We were surprised to find no coordination complexes reported for these diphosphines, despite them being synthesized two decades ago. In the Alder synthesis, the desired *cis*-macrocyclic diphosphines are obtained by stereoselective cleavage of the P–P bond in alkylated diphosphabicyclo[*k*.*l*.0]alkanes (*k* = 3–5, *l* = 3–4) by organometallic reagents. These diphosphabicyclo[*k*.*l*.0]alkanes are prepared *via* a tedious procedure from diphosphinoalkanes H_2_P–(CH_2_)_*k*_–PH_2_, which in turn are prepared in two steps from the corresponding dibromoalkanes. While the bicyclic diphosphane framework is key to the stereoselectivity of the entire process, its assembly represents the most cumbersome part of the synthesis. Our group has recently accessed bicyclic diphosphanes in only one step from white phosphorus and commercial dienes under photochemical conditions.^11,12^ For example, diphosphane 3,4,8,9-tetramethyl-1,6-diphosphabicyclo(4.4.0)deca-3,8-diene or P_2_(dmb)_2_ (**1**) is obtained in gram quantities directly from white phosphorus and 2,3-dimethylbutadiene.^11^ This synthesis is part of a greater endeavor pursued by our group^13^ and others^14^ to prepare phosphorus-containing compounds in an atom economical fashion directly from P_4_,^15^ the precursor to the more widely used phosphorus source PCl_3_.

We envisioned that alkylating **1** would yield phosphino-phosphonium salts that would be prone to reacting with nucleophiles to break the P–P bond. This process would afford *cis*-macrocyclic diphosphines in a stereoselective manner in only three steps from white phosphorus! We describe herein an expedited synthetic route to a family of *cis*-macrocyclic diphosphines that will facilitate investigating the coordination chemistry of these molecules and enable their use as ligands in catalysis.

We began this synthetic endeavor by investigating the alkylation of diphosphane **1** with methyl iodide (MeI). Previous studies have shown excellent selectivity in functionalizing only one of the phosphorus atoms in **1**.^16^ As expected, phosphino-phosphonium salt [Me-P_2_(dmb)_2_]I (**2**) was obtained in 90% yield after treatment of **1** with methyl iodide in diethyl ether at room temperature overnight. The structure of [Me-P_2_(dmb)_2_]I was confirmed by X-ray crystallography (Fig. 2), and displayed a typical P–P single bond distance of 2.1862(17) Å. Treatment of **1** with benzyl bromide (BnBr) also proceeded smoothly to give [Bn-P_2_(dmb)_2_]Br (**3**) in 96% yield after 2 hours at room temperature in dichloromethane (Fig. 1). The reaction of **1** with isobutyl bromide (^*i*^BuBr) was more sluggish and required heating at 100 °C for 20 h in the presence of an excess of the alkyl halide in order to achieve full conversion to [^*i*^Bu-P_2_(dmb)_2_]Br (**4**).^17^ All of these phosphino-phosphonium salts have characteristic ^31^P{^1^H} NMR spectra that show strong coupling between the two phosphorus atoms. For example, the ^31^P{^1^H} NMR shifts of [Me-P_2_(dmb)_2_]I are +44.7 ppm and –69.3 ppm, with a ^1^ *J*_PP_ = 276 Hz (see Table S1 in ESI† for a summary of all the ^31^P{^1^H} NMR chemical shifts and coupling constants).

**Fig. 1:**
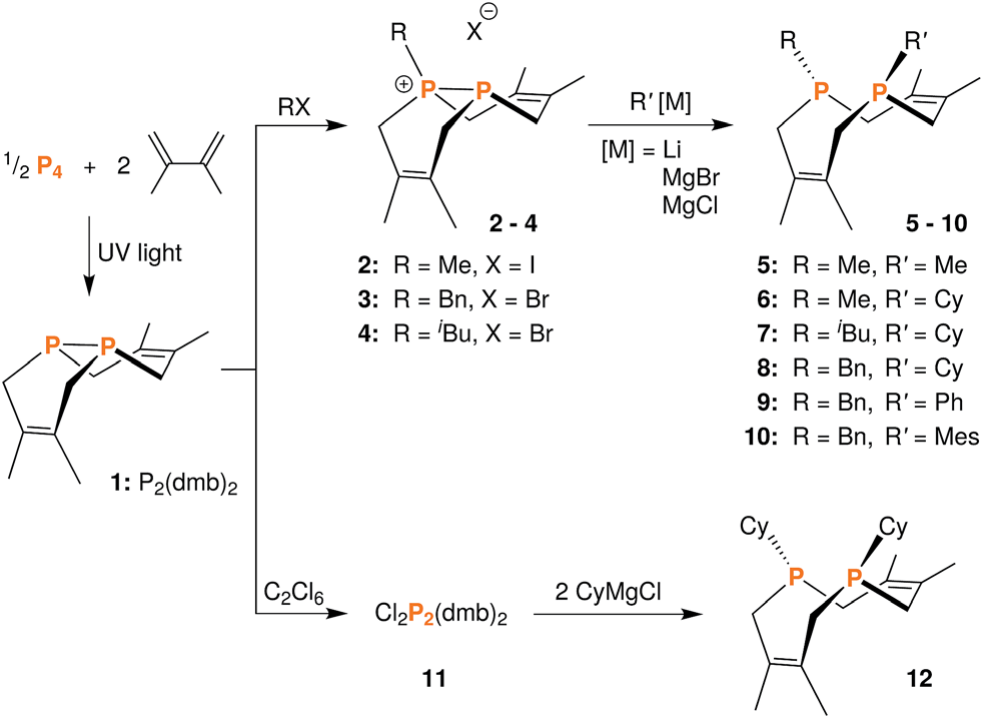
Synthetic routes to phosphino-phosphonium salts **2–4**, dichloride **11**, and macrocyclic diphosphines **5–10** and **12**.

**Fig. 2:**
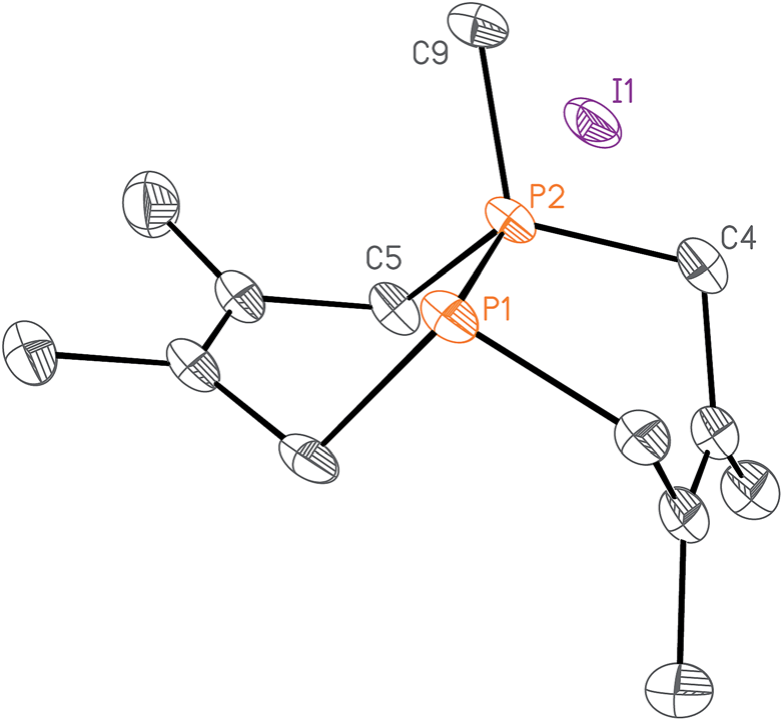
Solid-state structure of [Me-P_2_(dmb)_2_]I (**2**) with ellipsoids at the 50% probability level and disordered THF omitted for clarity. Representative interatomic distances [Å] and angles [°]: P2–C4 1.813(5), P2–C5 1.817(5), P2–C9 1.791(5), P1–P2 2.1862(17); C9–P2–C4 111.7(2), C9–P2–C5 106.9(2), C4–P2–C5 112.0(2), C9–P2–P1 114.01(19).

With these salts in hand, we began investigating their reactivity with organometallic reagents. Upon adding a methyllithium solution to a slurry of **2** in THF or diethyl ether at room temperature, a new signal indicative of *cis*-1,3,4,6,8,9-hexamethyl-2,5,7,10-tetrahydro-1,6-DiPhospheCine, or Me_2_-DPC (**5**), was observed by ^31^P{^1^H} NMR spectroscopy. A potential side reaction we had considered was deprotonation of the phosphino-phosphonium salt by methyllithium and formation of the Wittig reagent (CH_2_)P_2_(dmb)_2_. However, no evidence was found for the formation of such an ylide by ^31^P{^1^H} NMR spectroscopy. Diphosphine **5** was also characterized by X-ray crystallography (Fig. 3), revealing a conformation with nearly *C*_2v_ symmetry in which the phosphorus lone pairs are pointed away from each other.

**Fig. 3:**
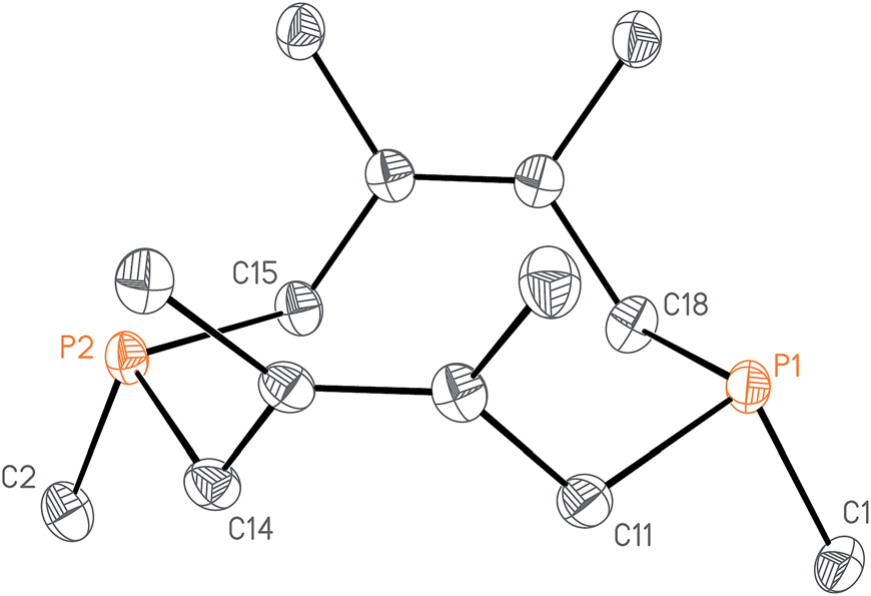
Solid-state structure of Me_2_-DPC (**5**) with ellipsoids at the 50% probability level and hydrogen atoms omitted for clarity. Representative interatomic distances [Å] and angles [°]: P1–C1 1.8433(16), P1–C11 1.8583(16), P1–C18 1.8624(16), P2–C2 1.8421(17), P2–C15 1.8617(16), P2–C14 1.8630(16); C1–P1–C11 96.84(7), C1–P1–C18 98.07(7), C11–P1–C18 101.22(7), C2–P2–C15 97.03(8), C2–P2–C14 98.02(7), C15–P2–C14 101.54(7).

Treatment of phosphino-phosphonium salts **2**, **4**, and **3** with cyclohexylmagnesium chloride led to the formation of Me,Cy-DPC (**6**), ^*i*^Bu,Cy-DPC (**7**), and Bn,Cy-DPC (**8**), respectively (Fig. 1). All of these diphosphines can be isolated in yields of 90–94% and they each show two distinct signals in their ^31^P{^1^H} NMR spectra, with small ^5^ *J*_PP_ coupling constants of *ca.* 5 Hz. For example, the ^31^P{^1^H} NMR spectrum of **6** shows two signals at –38.9 and –60.8 ppm. In order to demonstrate the versatility of this synthetic strategy, **3** was also treated with two aryl Grignard reagents, phenylmagnesium bromide and mesitylmagnesium bromide. The resulting diphosphines, Bn,Ph-DPC (**9**) and Bn,Mes-DPC (**10**), showed similar spectroscopic features to the dialkyl diphosphines described above.

While this modular approach had already enabled the synthesis of a variety of macrocyclic diphosphines, the initial alkylation step was limited to primary halide substrates. In order to overcome this limitation, we sought to add a dihalodiphosphine to our synthetic toolbox. This intermediate would provide access to a symmetrical diphosphine upon addition of two equivalents of organometallic reagent. Treatment of **1** with iodine, a mild oxidant, did not yield a diiododiphosphine, but rather an iodine adduct, I_2_·**1**, which we were able to characterize by X-ray crystallography (Fig. 4). The elongated I–I distance of 3.4169(12) Å is similar to metrical data reported for other phosphine–iodine adducts.^18^ While several monophosphine–iodine adducts have been structurally characterized and described in the literature,^18,19^ this is an unusual example of a diphosphane–iodine adduct in which the P–P bond is still intact.

**Fig. 4:**
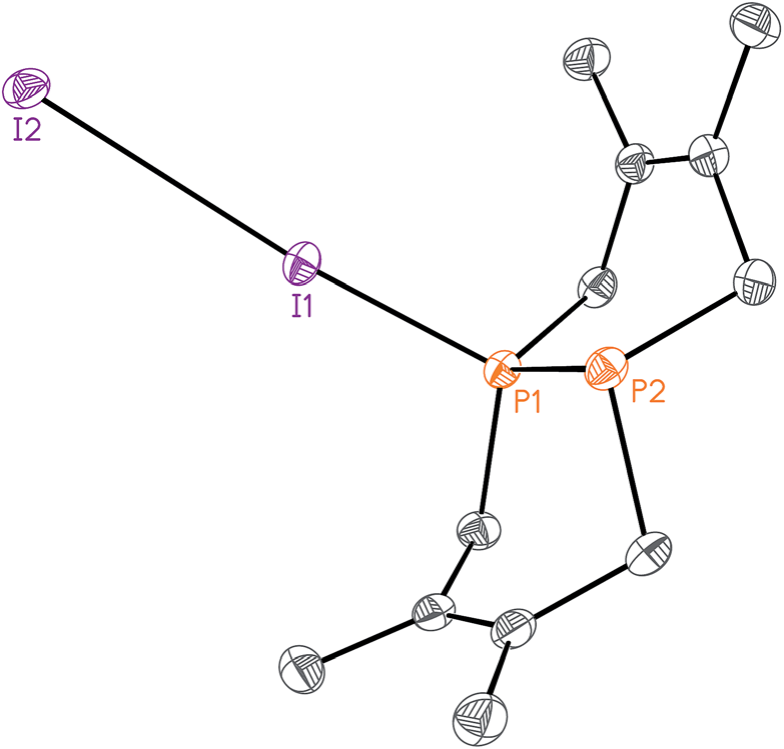
Solid-state structure of I_2_·P_2_(dmb)_2_ (I_2_·**1**) with ellipsoids at the 50% probability level and disordered solvent and iodine omitted for clarity. Representative interatomic distances [Å] and angles [°]: I1–P1 2.4110(5), I1–I2 3.4169(12), P1–P2 2.1913(7); P1–I1–I2 173.21(4).

The reaction of **1** with many potent halogenating agents (*e.g.* bromine, *N*-bromosuccinimide, xenon difluoride) proved to be unselective, yet oxidation of **1** with hexachloroethane produced the desired Cl_2_P_2_(dmb)_2_ (**11**) in good purity. We expected this product to have a symmetrical open structure, as the P–P bond would have been cleaved upon oxidation. However, NMR spectroscopy suggests that **11** is best described as a chloronium chloride salt in equilibrium with the open form. At room temperature, signals are broad in both the ^1^H NMR and ^31^P{^1^H} NMR (Δ*ν*_1/2_ ≈ 1700 Hz) spectra of **11**; upon cooling to –40 °C, the ^31^P{^1^H} NMR spectrum shows two distinct phosphorus environments at +143.4 ppm and –48.7 ppm with a ^1^ *J*_PP_ = 291 Hz. In order to elucidate whether the broad NMR signals were due to chloride association/dissociation at the same phosphorus center *versus* chloride-mediated P–P bond scission/reformation, a 2D EXSY (EXchange SpectroscopY) experiment was performed.^20^ In the first pathway, the two phosphorus centers would remain distinct throughout the exchange process, while in the second pathway the two phosphorus centers would become chemically equivalent in the open form. The ^31^P{^1^H} 2D EXSY spectrum of **11** (Fig. 5) clearly shows the presence of exchange cross peaks, thus supporting the chemical exchange pathway depicted in the same figure. The proposed role of free chloride in the exchange was corroborated by treatment of **11** with GaCl_3_, which sequestered the chloride into the tetrachlorogallate [GaCl_4_]^–^ anion; this inhibited the exchange process and “froze” the compound in the chloronium salt form with sharp ^31^P{^1^H} NMR resonances at room temperature.

**Fig. 5:**
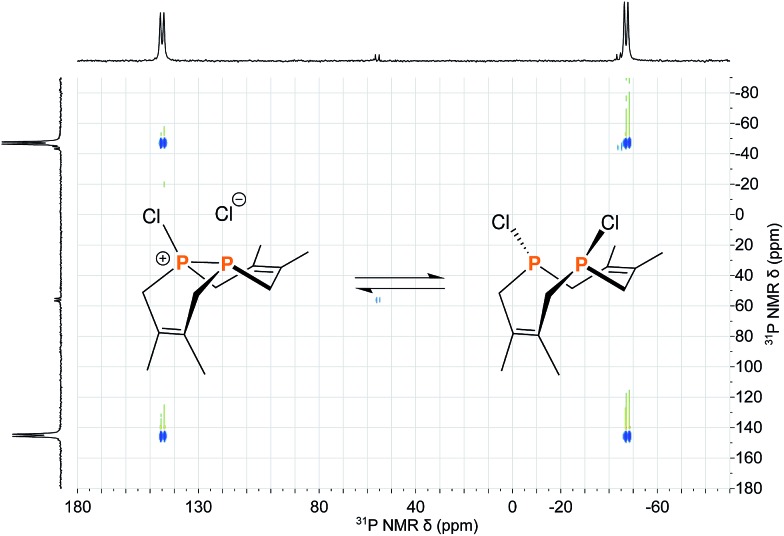
Reaction scheme depicting the equilibrium between the two structural forms of **11** overlayed on the ^31^P{^1^H} 2D EXSY spectrum of **11** acquired at 0 °C in CDCl_3_.

Addition of two equivalents of cyclohexylmagnesium chloride to a suspension of **11** in diethyl ether or THF results in the formation of the desired Cy_2_-DPC (**12**). The ^31^P{^1^H} NMR spectrum of this product reveals a characteristic singlet resonance at –38.7 ppm for **12**, but also a small amount of **1** (*ca.* 2%). This by-product is likely due to the reduction of **11** under the reaction conditions. While reduction was not a major reaction pathway during the formation of **12**, it became problematic when attempting to prepare ^*t*^Bu_2_-DPC. Treatment of **11** with *tert*-butylmagnesium chloride resulted in a roughly equimolar mixture of ^*t*^Bu_2_-DPC and **1**. Due to their similar solubility properties, these two products could not be separated.

While all syntheses were conducted under inert atmosphere, we wondered if these new diphosphines had any stability to air and moisture. Exposing a benzene-*d*_6_ solution of **12** to air led to no visible changes by ^31^P NMR spectroscopy even after 24 hours. This diphosphine proved to be remarkably robust, as less than 10% of the material had decomposed to the phosphine–phosphine oxide after 48 hours in solution.

Having accessed a family of new chelating macrocyclic diphosphines, we were interested in exploring their use as supporting ligands in a catalytic process. Given our interest in CO_2_ utilization,^21^ we turned to the coupling of CO_2_ and ethylene as a first application. While formation of nickelalactones from Ni(0) species, CO_2_ and ethylene had been known since the 1980s from the pioneering work of Hoberg and coworkers,^22^ the first catalytic system to produce acrylate from this coupling was only reported in 2012.^23,24^ The stability of nickelalactones has been one of the major challenges in assembling a catalytic process; conversion of a nickelalactone to its corresponding nickel acrylate complex requires both a base and an alkali metal Lewis acid.^23,25,26^ Furthermore, extensive ligand screening has shown that only a small, select group of electron-rich diphosphines gives rise to any catalytic activity whatsoever.^27,28^ Despite the progress made in the last few years, typical turnover numbers are in the double digits and only extensively optimized systems yield turnover numbers greater than 100.^28^

In order to test whether these *cis*-macrocyclic diphosphines could support a nickelalactone, we prepared (Fig. 6) and crystallographically characterized the nickelalactone of **12**, (Cy_2_-DPC)Ni(CH_2_CH_2_COO) (**13**) (Fig. 7). The Ni1–C3 and Ni1–O1 distances of 1.982(6) and 1.899(4) Å, respectively, are similar to those reported for other nickelalactones.^29^ The bite angle of the diphosphine in complex **13** is 91.46(5)°, a value intermediate between that observed for the lactone of dicyclohexylphosphinoethane (dcpe), namely 88.07(5)°,^26^ and that observed for the lactone of diphenylphosphinobutane (dppb), namely 93.91(4)°.^29^ The smaller bite angle compared to dppb, a ligand that also has a four carbon atom bridge, might be due to the increased backbone rigidity of **12**.

**Fig. 6:**
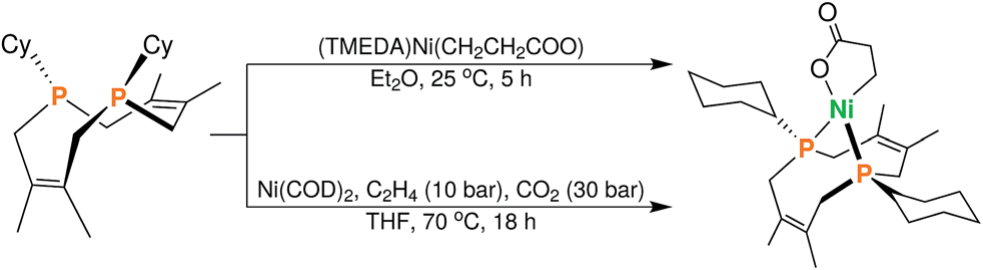
Synthesis of (Cy_2_-DPC)Ni(CH_2_CH_2_COO) (**13**) from **12** *via* two complementary routes (see ESI† for details).

**Fig. 7:**
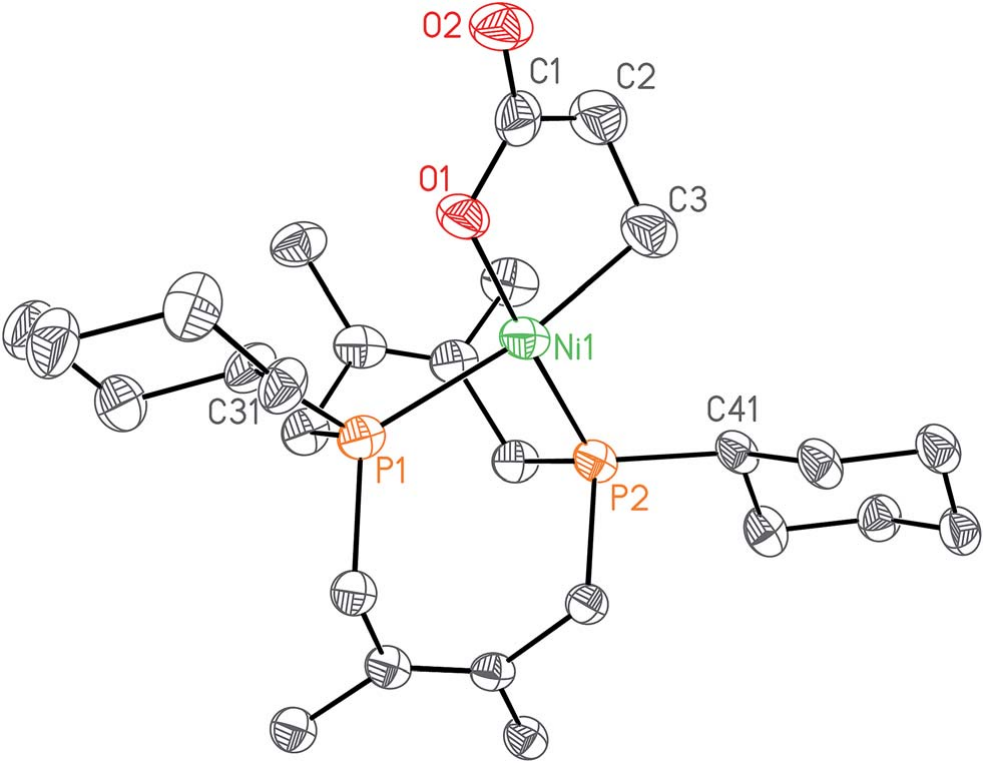
Solid-state structure of nickelalactone (Cy_2_-DPC)Ni(CH_2_CH_2_COO) (**13**) with ellipsoids at the 50% probability level and hydrogen atoms omitted for clarity. Representative interatomic distances [Å] and angles [°]: Ni1–P1 2.2454(15), Ni1–P2 2.1283(15), Ni1–C3 1.982(6), Ni1–O1 1.899(4), O1–C1 1.283(6), O2–C1 1.225(6); O1–Ni1–P1 90.86(12), O1–Ni1–C3 85.3(2), C3–Ni1–P2 92.56(19), P2–Ni1–P1 91.46(5); P1–Ni1–P2–C41–173.1(2), P2–Ni1–P1–C31 175.33(18).

While helpful for comparing **12** with other ligands previously used to support nickelalactones, bite angle is an insufficient metric for describing the steric profile of **12**. The cyclic backbone of this ligand renders an unusual placement of the cyclohexyl substituents in the P–Ni–P plane, a feature best described by the P1–Ni1–P2–C41 and P2–Ni1–P1–C31 torsion angles of –173.1(2)° and 175.33(18)°, respectively. In contrast, a ligand such as dcpe places its backbone linker in the P–Ni–P plane, and the cyclohexyl substituents above and below that plane (Fig. 8). The absolute values of the corresponding P–Ni–P–C torsion angles in (dcpe)Ni(CH_2_CH_2_COO) range from 96.6° to 136.7°, and average 117°.^26^ In effect, **12** has its cyclohexyl substituents rotated by 60° compared to a typical diphosphine such as dcpe.

**Fig. 8:**
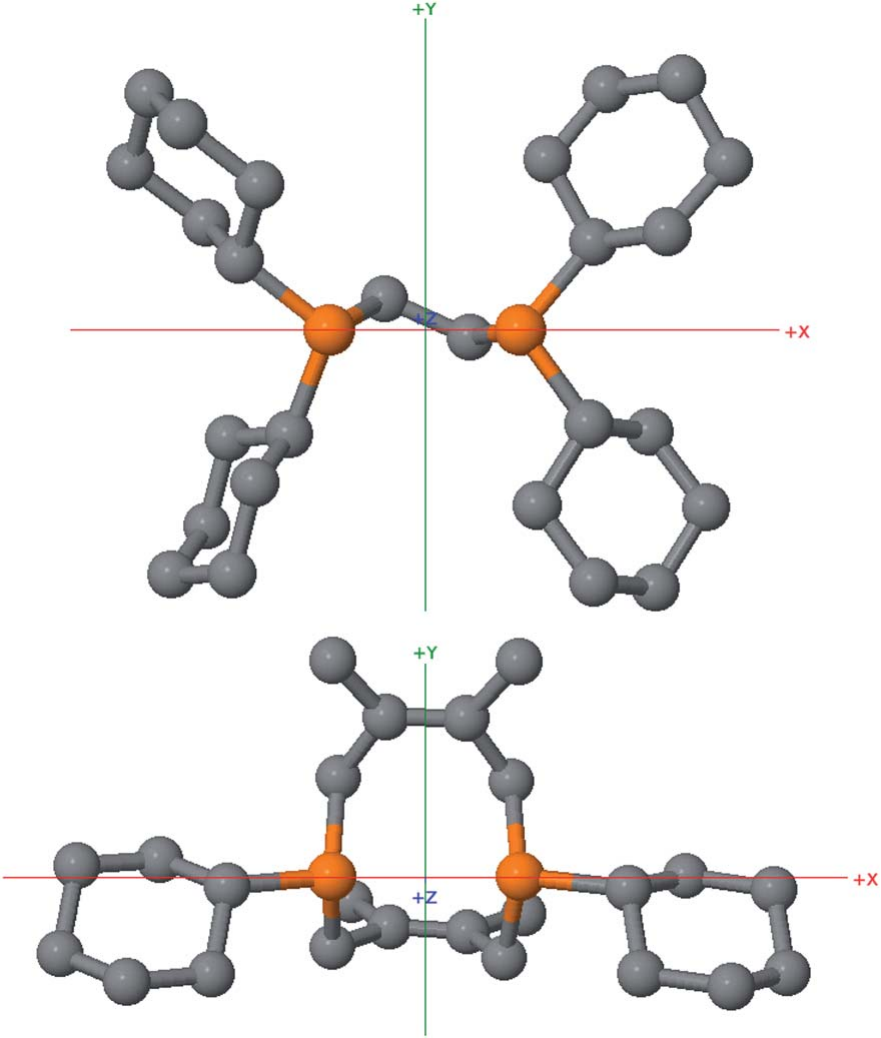
Ball and stick models of dcpe (top) and Cy_2_-DPC (bottom) with hydrogen atoms omitted for clarity, highlighting the differences in steric profiles between these two diphosphines. Both ligands are viewed along the *Z* axis, defined using Samb*V*ca 2.0 as the P–Ni–P angle bisector in their respective nickelalactone complexes.

In order to further probe the comparison between dcpe and **12**, their % buried volumes (%*V*_bur_, defined as the percent volume occupied by the ligand out of the total volume of a sphere centered at the metal and typically set to have a 3.5 Å radius)^30^ were calculated^31^ using the Samb*V*ca 2.0 web application developed by Cavallo and coworkers.^32^ Even though dcpe has four cyclohexyl substituents and **12** has only two, these ligands have almost identical % buried volumes: 54.5% and 54.8%, respectively. This result can be in part attributed to the unusual placement of the cyclohexyl groups in **12** with respect to the metal center, but also to the significant bulk added by the protruding methyl groups of the tetramethyltetrahydroDiPhospheCine (DPC) backbone.

Having determined that nickelalactones are accessible with this new ligand platform, we proceeded in measuring the catalytic activity of diphosphines **5–10** and **12**. In doing so, we used two different protocols: one adapted from the work of Vogt *et al.* (method A),^25^ and one adapted from Limbach *et al.* (method B).^27^ In a typical catalytic run, Ni(COD)_2_ was mixed with the ligand, along with a base, Lewis acid, and zinc as an additive. For runs using method A, the base was NEt_3_ and the Lewis acid was LiI, whereas for runs using method B, sodium 2-fluorophenoxide served as both the base and source of Lewis acid. As shown in Table 1, all of the new ligands but the bulky Bn,Mes-DPC (**10**) showed catalytic activity in this transformation. Dicyclohexylphosphinoethane (dcpe), dicyclohexylphosphinopropane (dcpp) and dicyclohexylphosphinobutane (dcpb) were used as benchmarks, as they are some of the best performing ligands reported in the literature so far.^25,27^ We found the turnover numbers of our most active ligands such as **12** to be comparable to or better than those of the benchmark ligands. Notably, **12** performed significantly better than dcpb, a ligand that has a similar 4-carbon atom backbone.

**Table 1:** Catalytic acrylate production from CO_2_ and ethylene

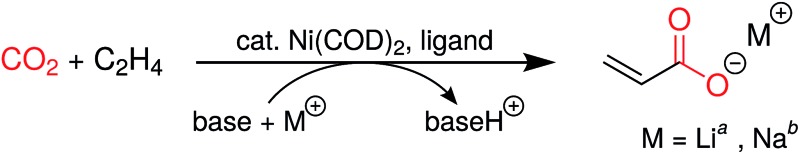		
Ligand	TON^*a*^	TON^*b*^
Me_2_-DPC (**5**)	1	6
Me,Cy-DPC (**6**)	4	15
^*i*^Bu,Cy-DPC (**7**)	10	11
Bn,Cy-DPC (**8**)	9	17
Bn,Ph-DPC (**9**)	12	4
Bn,Mes-DPC (**10**)	0	0
Cy_2_-DPC (**12**)	12	19 (16)^*c*^
Cy_2_P–(CH_2_)_2_–PCy_2_ (dcpe)	8	12
Cy_2_P–(CH_2_)_3_–PCy_2_ (dcpp)	18	7
Cy_2_P–(CH_2_)_4_–PCy_2_ (dcpb)	6	4

^*a*^Ni(COD)_2_ (0.05 mmol), ligand (0.05 mmol), Zn (2.5 mmol), LiI (1.25 mmol), NEt_3_ (2.5 mmol), PhCl (2 mL), pressurized with C_2_H_4_ (25 bar) and CO_2_ (5 bar) and heated to 50 °C for 24 h. The TONs listed are averages of two independent runs.

^*b*^Ni(COD)_2_ (0.07 mmol), ligand (0.077 mmol), Zn (3.5 mmol), sodium 2-fluorophenoxide (3.5 mmol), THF (10 mL), pressurized with C_2_H_4_ (10 bar) and CO_2_ (10 bar) and heated to 100 °C for 20 h.

^*c*^Complex **13** (0.07 mmol) was used as the starting nickel source *in lieu* of the typical mixture of Ni(COD)_2_ and ligand.

Employing this new family of *cis*-macrocyclic diphosphines in CO_2_/ethylene coupling is only the first step in exploring the catalytic relevance of these compounds. We plan to expand this ligand family using the synthetic methods disclosed herein, which have laid the groundwork for accessing phosphorus-based ligands with unique structures and steric profiles in a modular fashion. Asymmetric ligands will be easily accessible by installing chiral groups on the phosphorus centers, thus enabling numerous applications in the realm of asymmetric catalysis. Given the unusual steric profiles of the *cis*-macrocyclic diphosphines reported herein, our group is also actively pursuing the synthesis and characterization of a variety of metal complexes supported by these ligands in order to learn more about their coordination chemistry.
