# Supplementary material for: A family of cis-macrocyclic diphosphines: modular, stereoselective synthesis and application in catalytic CO2/ethylene coupling
Source: Chem Sci. 2016 Oct 11;8(2):1463–8. doi: 10.1039/c6sc03614g (PMC5460601; doi:10.1039/c6sc03614g)
Supplement: Supplementary file 1 [file SC-008-C6SC03614G-s001.pdf]

Electronic Supplementary Material (ESI) for Chemical Science.
This journal is © The Royal Society of Chemistry 2016

Supporting Information

A family of *cis*-macrocyclic diphosphines: stereoselective synthesis and application in catalytic CO_2/ethylene coupling

Ioana Knopf,[a] Daniel Tofan,[a] Dirk Beetstra,[b] Abdulaziz Al-Nezari,[b] Khalid Al-Bahily,[b] and Christopher C. Cummins [*,a]

[a] *Department of Chemistry, Massachusetts Institute of Technology, 77 Massachusetts Avenue, Cambridge, MA 02139-4307, USA*
[b] *SABIC CRD, Fundamental Catalysis, Thuwal 23955-6900, Saudi Arabia*

Contents

1 General Methods **S3**

2 Summary of $^{31}P\{^{1}H\}$ NMR spectroscopic data for compounds 1–13 **S4**

3 [Me–P_2(dmb)$_2$]I (2) **S5**

3.1 **Synthesis of [Me–P_2(dmb)$_2$]I (2)** S5

3.2 **Characterization of [Me–P_2(dmb)$_2$]I (2)** S5

4 [Bn–P_2(dmb)$_2$]Br (3) **S8**

4.1 **Synthesis of [Bn–P_2(dmb)$_2$]Br (3)** S8

4.2 **Characterization of [Bn–P_2(dmb)$_2$]Br (3)** S8

5 [^{i}Bu–P_2(dmb)$_2$]Br (4) **S11**

5.1 **Synthesis of [^{i}Bu–P_2(dmb)$_2$]Br (4)** S11

5.2 **Characterization of [^{i}Bu–P_2(dmb)$_2$]Br (4)** S11

6 Me_2–DPC (5) **S14**

6.1 **Synthesis of Me_2–DPC (5)** S14

6.2 **Characterization of Me_2–DPC (5)** S14

7 Me,Cy–DPC (6) **S17**

7.1 **Synthesis of Me,Cy–DPC (6)** S17

7.2 **Characterization of Me,Cy–DPC (6)** S17

8 ^{i}Bu,Cy–DPC (7) **S20**

8.1 **Synthesis of ^{i}Bu,Cy–DPC (7)** S20

8.2 **Characterization of ^{i}Bu,Cy–DPC (7)** S20

9 Bn,Cy–DPC (8) **S23**

9.1 **Synthesis of Bn,Cy–DPC (8)** S23

9.2 **Characterization of Bn,Cy–DPC (8)** S23

10 Bn,Ph–DPC (9) **S26**

10.1 **Synthesis of Bn,Ph–DPC (9)** S26

10.2 **Characterization of Bn,Ph–DPC (9)** S26

11 Bn,Mes–DPC (10) **S29**

11.1 **Synthesis of Bn,Mes–DPC (10)** S29

11.2 **Characterization of Bn,Mes–DPC (10)** S29

12 $Cl_2P_2(dmb)_2$ (11) **S32**

12.1 **Synthesis of $Cl_2P_2(dmb)_2$ (11)** S32

12.2 **Characterization of $Cl_2P_2(dmb)_2$ (11)** S32

13 Cy_2–DPC (12) **S38**

13.1 **Synthesis of Cy_2–DPC (12)** S38

13.2 **Characterization of Cy_2–DPC (12)** S39

14 (Cy_2–DPC)Ni(CH_2CH_2COO) (13) **S42**

14.1 Synthesis of (Cy_2–DPC)Ni(CH_2CH_2COO) (**13**) S42

14.2 Characterization of (Cy_2–DPC)Ni(CH_2CH_2COO) (**13**) S43

15 Experimental details for CO_2/C_2H_4 coupling catalytic tests **S46**

16 Crystallographic details **S48**

1 General Methods

All manipulations were performed using standard Schlenk techniques or in a nitrogen atmosphere glovebox, unless otherwise stated. All reagents were purchased from Sigma Aldrich, Alfa Aesar or Strem Chemicals. Basic alumina was dried by heating at 250 °C under dynamic vacuum for at least 4 days prior to use. Solvents (EMD Chemicals) were either used as received or purified on a Glass Contour Solvent Purification System built by SG Water USA, LLC. IR spectra were recorded on a Bruker Tensor 37 Fourier transform IR (FTIR) spectrometer. Elemental analyses were performed by Robertson Microlit Laboratories, Inc. NMR solvents were obtained from Cambridge Isotope Laboratories, degassed and dried over 4 Å molecular sieves for at least 2 days prior to use. NMR spectra were obtained on a Bruker 400 MHz or a Varian 500 MHz spectrometer. ^{1}H NMR and ^{13}C{^{1}H} NMR chemical shifts are referenced to residual protio-solvent signals, and ^{31}P{^{1}H} NMR spectra are referenced to a 85% H_3PO_4 external standard (δ = 0 ppm). Many of the ^{1}H NMR and ^{13}C NMR spectra display non first order multiplets; in those cases, please be aware that the J values reported are only the *apparent* J values. The ^{31}P{^{1}H} 2D EXSY spectrum was acquired at 0 °C on a Varian 500 MHz spectrometer using typical 2D NOESY parameters, 64 t_1 increments, a spectral window of 180 to −90 ppm, and a mixing time of 50 ms. The cross peaks observed have the same phase as the diagonal peaks, and were therefore attributed to chemical exchange rather than NOE effects.[1–4]

Low-temperature (100 K) X-ray diffraction data were collected on a Bruker X8 Kappa DUO four-circle diffractometer coupled to a Bruker Smart APEX2 CCD detector with Mo Kα radiation (λ = 0.71073 Å) from an IμS micro-source for the structures of $I_2 \cdot P_2dmb_2$ ($I_2 \cdot$**1**) and (Cy_2–DPC)Ni(CH_2CH_2COO) (**13**) or on a Bruker D8 three-circle diffractometer coupled to a Bruker-AXS Smart Apex CCD detector with graphite-monochromated Cu Kα radiation (λ = 1.54178 Å) for the structures of [Me–$P_2(dmb)_2$]I (**2**) and Me_2–DPC (**5**). Data reduction was done using SAINT[5] and absorption correction was done using SADABS.[6] The structures were solved by direct methods using SHELXS[7] or intrinsic phasing using SHELXT[8] and refined against F^2 on all data by full-matrix least squares with SHELXL-2013[9] using established methods.[10,11] All non-hydrogen atoms were refined anisotropically. All hydrogen atoms were included in the model at geometrically calculated positions and refined using a riding model.

2 Summary of $^{31}P\{^1H\}$ NMR spectroscopic data for compounds 1−13

Table S1 $^{31}P\{^1H\}$ NMR chemical shifts of compounds **1**−**13** and corresponding P–P coupling constants

Compound	Solvent	Temp.	δ P1 (ppm)	δ P2 (ppm)	J_{PP} (Hz)
$P_2(dmb)_2$ (**1**)[12]	C_6D_6	25 °C	−53.8	–	–
[Me–$P_2(dmb)_2$]I (**2**)	$CDCl_3$	25 °C	+44.7	−69.3	276
[Bn–$P_2(dmb)_2$]Br (**3**)	$CDCl_3$	25 °C	+50.8	−72.3	293
[^{i}Bu–$P_2(dmb)_2$]Br (**4**)	$CDCl_3$	25 °C	+48.0	−70.9	286
Me_2–DPC (**5**)	C_6D_6	25 °C	−60.9	–	–
Me,Cy–DPC (**6**)	C_6D_6	25 °C	−38.9	−60.8	5
^{i}Bu,Cy–DPC (**7**)	C_6D_6	25 °C	−38.9	−54.6	5
Bn,Cy–DPC (**8**)	C_6D_6	25 °C	−38.9	−44.7	5
Bn,Ph–DPC (**9**)	C_6D_6	25 °C	−45.5	−46.1	6
Bn,Mes–DPC (**10**)	C_6D_6	25 °C	−45.6	−47.5	9
$Cl_2P_2(dmb)_2$ (**11**)	$CDCl_3$	−40 °C	+143.4	−48.7	291
Cy_2–DPC (**12**)	C_6D_6	25 °C	−38.7	–	–
(Cy_2–DPC)Ni(CH_2CH_2COO) (**13**)	C_6D_6	25 °C	+21.6	−12.9	37

3 [Me–P_2(dmb)$_2$]I (2)

3.1 Synthesis of [Me–P_2(dmb)$_2$]I (2)

Inside the glovebox, P_2dmb$_2$ (1.36 g, 6.0 mmol, 1 equiv) was dissolved in 100 mL of Et_2O in a 200 mL round bottom flask containing a stir bar. To this homogeneous solution, methyl iodide (0.5 mL, 1.14 g, 8.0 mmol, 1.33 equiv) was added by syringe. The reaction flask was capped with a septum and the reaction mixture was allowed to stir at room temperature overnight. After ca. 14 h, the reaction mixture, now heterogeneous, was put under vacuum to remove volatiles. The solids were slurried in ca. 20 mL Et_2O, then filtered and washed with 2 x 10 mL Et_2O. After drying the solids, 1.985 g of white powder were obtained (5.39 mmol, 90% yield).

3.2 Characterization of [Me–P_2(dmb)$_2$]I (2)

^{1}H NMR (400 MHz, $CDCl_3$, 25 °C) δ: 3.63 (t, J = 14.8 Hz, 2H), 3.45 (t, J = 15.0 Hz, 2H), 2.98 – 2.64 (m, 4H), 2.35 (dd, J = 13.6, 5.6 Hz, 3H), 1.88 (s, 6H), 1.80 (d, J = 5.8 Hz, 6H) ppm.

^{13}C NMR (101 MHz, $CDCl_3$, 25 °C) δ: 131.31 (dd, J = 13.0, 2.1 Hz), 122.46 (dd, J = 10.5, 4.6 Hz), 27.62 (dd, J = 27.0, 5.3 Hz), 26.80 (dd, J = 29.4, 2.7 Hz), 21.35 (d, J = 2.0 Hz), 21.31 (d, J = 1.3 Hz), 9.07 (dd, J = 46.4, 20.3 Hz) ppm.

^{31}P{^{1}H} NMR (162 MHz, $CDCl_3$, 25 °C) δ: 44.71 (d, J = 276.0 Hz), −69.30 (d, J = 276.0 Hz) ppm.

ATR-IR: 2917, 1442, 1195, 1124, 1064, 937, 846, 815, 703, 417 cm^{-1}.

Elemental analysis [%] found (calculated for $C_{13}H_{23}IP_2$): C 42.36 (42.41), H 5.78 (6.30), N $<$ 0.02 (0).

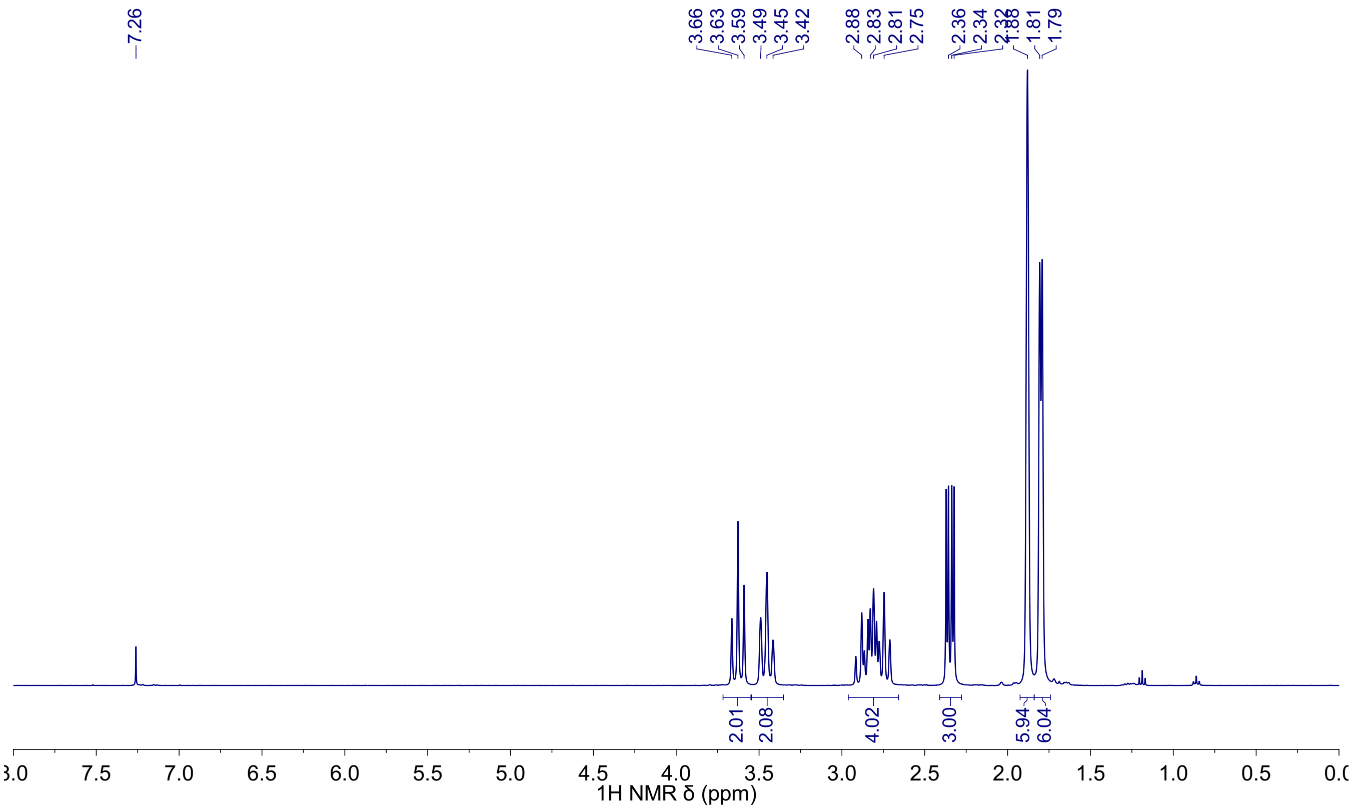

Figure S1 ^{1}H NMR spectrum of [Me–P_2(dmb)$_2$]I in $CDCl_3$ at 25 °C

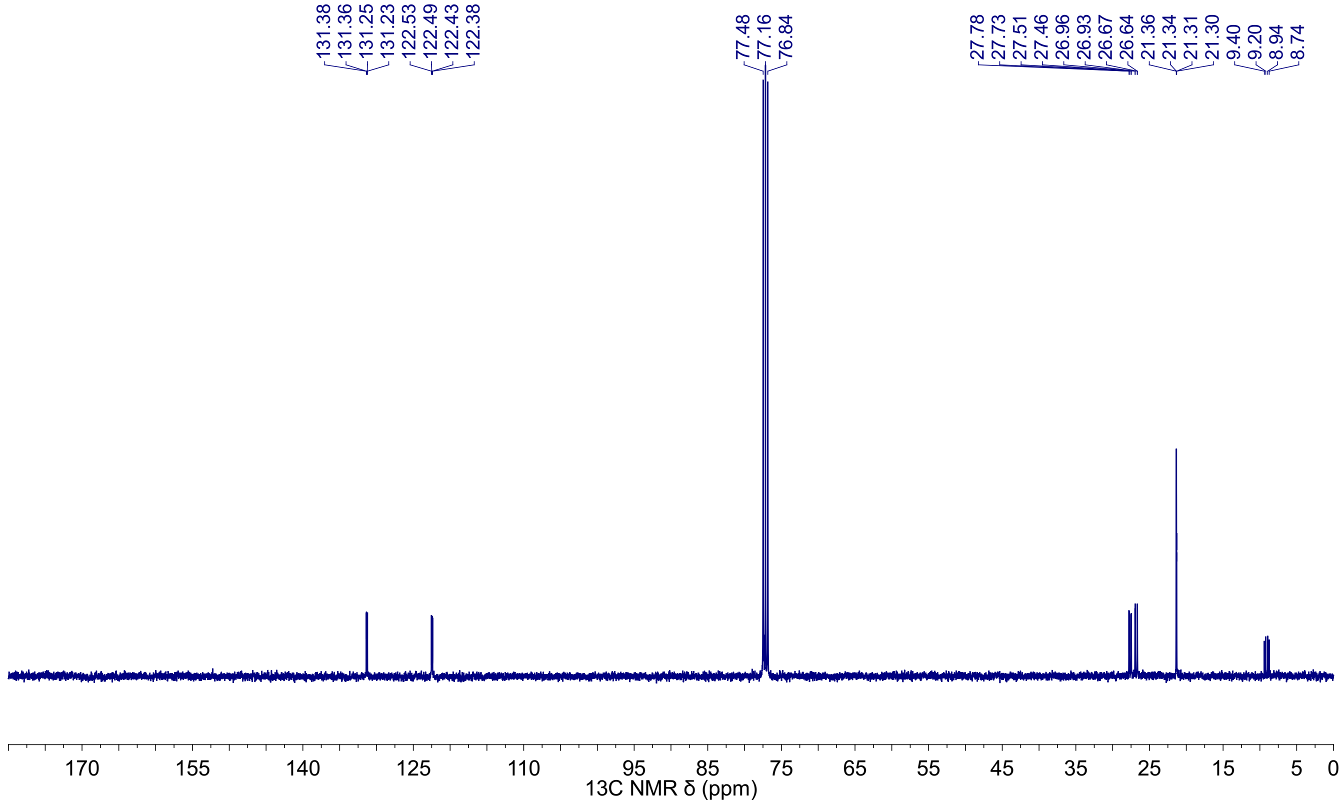

Figure S2 ^{13}C NMR spectrum of [Me–P_2(dmb)$_2$]I in $CDCl_3$ at 25 °C

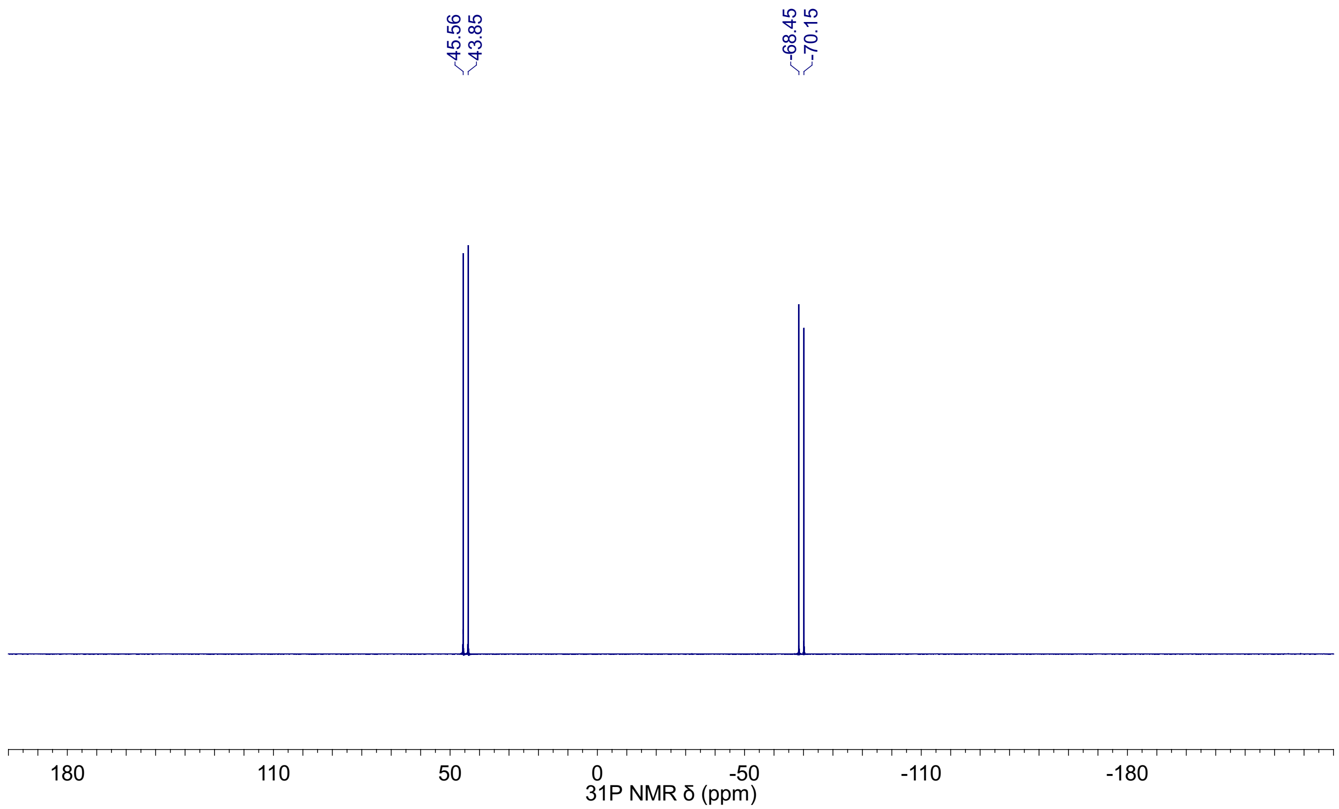

Figure S3 $^{31}P\{^{1}H\}$ NMR spectrum of [Me–P_2(dmb)$_2$]I in $CDCl_3$ at 25 °C

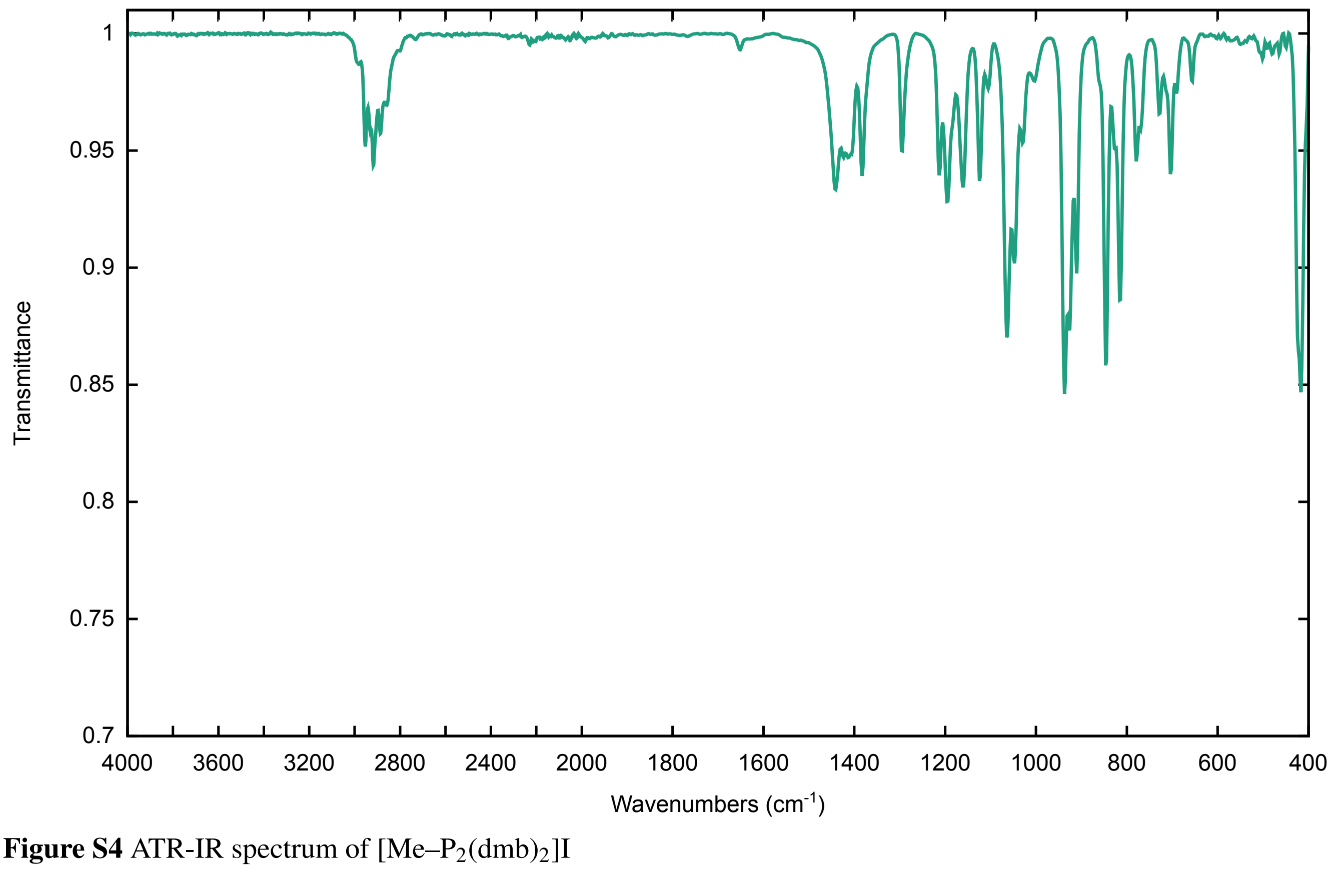

Figure S4 ATR-IR spectrum of [Me–P_2(dmb)$_2$]I

4 [Bn–P_2(dmb)$_2$]Br (3)

4.1 Synthesis of [Bn–P_2(dmb)$_2$]Br (3)

Inside the glovebox, P_2dmb$_2$ (1.195 g, 5.28 mmol, 1 equiv) was dissolved in 30 mL of dichloromethane in a 200 mL round bottom flask containing a stir bar. To this homogeneous solution, benzyl bromide (0.75 mL, 1.08 g, 6.34 mmol, 1.2 equiv) was added by syringe. The reaction flask was capped with a septum and the reaction mixture was allowed to stir at room temperature. Even after 5 min, a visible amount of white precipitate had formed. The mixture was allowed to stir for 2 hours, after which it was put under vacuum to remove volatiles. The solids were triturated with Et_2O. In order to remove the remaining excess benzyl bromide, the solid was slurried in 15 mL pentane, filtered, then washed with 2 x 7 mL pentane. After drying the solids, 2.019 g of white powder were obtained (5.08 mmol, 96% yield).

4.2 Characterization of [Bn–P_2(dmb)$_2$]Br (3)

^{1}H NMR (400 MHz, $CDCl_3$, 25 °C) δ: 7.60 – 7.46 (m, 2H), 7.39 – 7.27 (m, 3H), 4.38 (dd, J = 14.5, 4.5 Hz, 2H), 3.45 (t, J = 14.9 Hz, 2H), 3.27 (t, J = 14.8 Hz, 2H), 2.87 – 2.49 (m, 4H), 1.75 (d, J = 5.5 Hz, 6H), 1.71 (s, 6H) ppm.

^{13}C NMR (101 MHz, $CDCl_3$, 25 °C) δ: 130.85 (d, J = 2.6 Hz), 130.77 (dd, J = 7.0, 2.6 Hz), 129.37 (d, J = 3.3 Hz), 129.03 (d, J = 9.1 Hz), 128.39 (d, J = 3.9 Hz), 122.25 (dd, J = 10.1, 4.4 Hz), 29.04 (dd, J = 37.5, 16.2 Hz), 27.44 (dd, J = 27.1, 5.3 Hz), 24.74 (dd, J = 27.6, 2.7 Hz), 21.47 (d, J = 3.3 Hz), 21.08 (dd, J = 4.8, 1.1 Hz) ppm.

^{31}P{^{1}H} NMR (162 MHz, $CDCl_3$, 25 °C) δ: 50.84 (d, J = 292.5 Hz), −72.27 (d, J = 292.5 Hz) ppm.

ATR-IR: 2913, 1494, 1439, 1065, 796, 698, 480, 423 cm^{-1}.

Elemental analysis [%] found (calculated for $C_{19}H_{27}P_2Br$): C 57.18 (57.44), H 6.61 (6.85), N $<$ 0.02 (0).

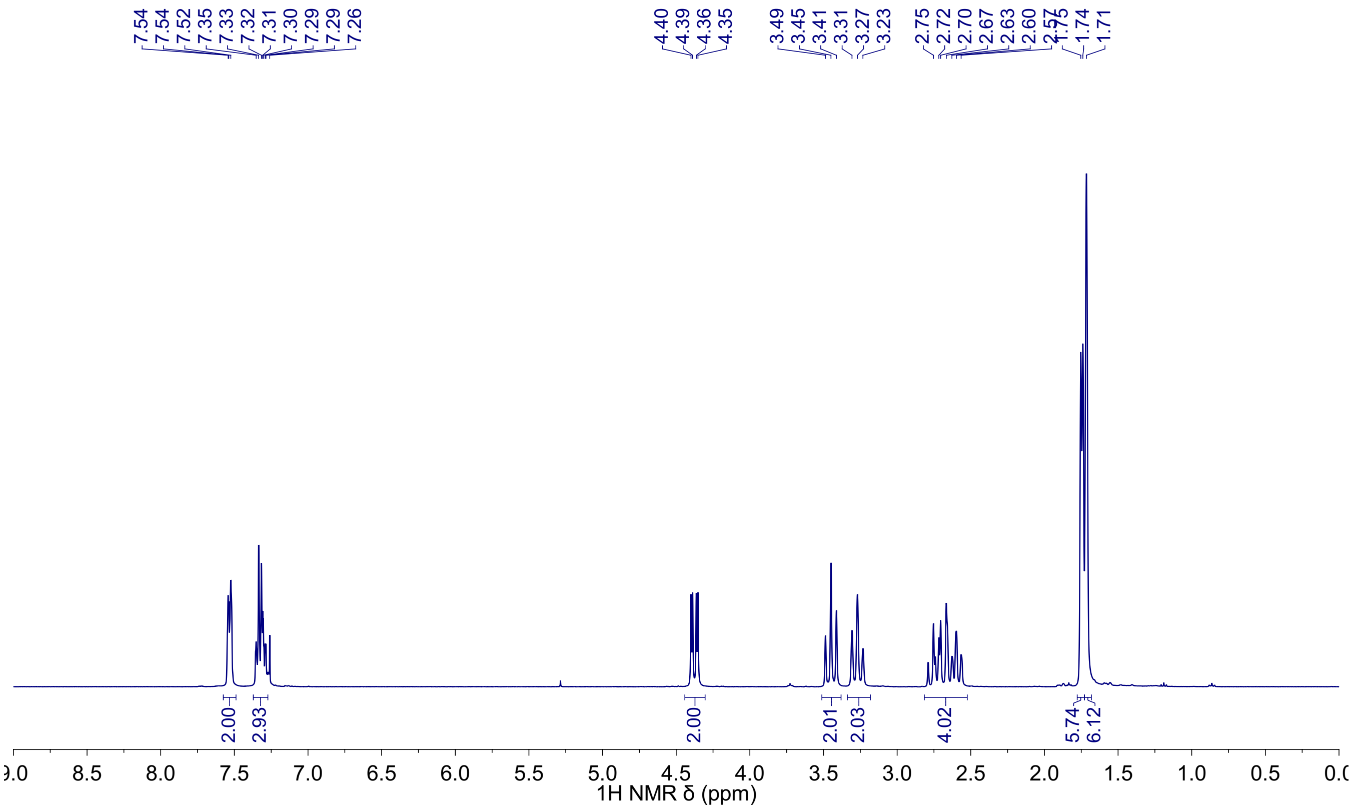

Figure S5 ^{1}H NMR spectrum of [Bn–P_2(dmb)$_2$]Br in $CDCl_3$ at 25 °C

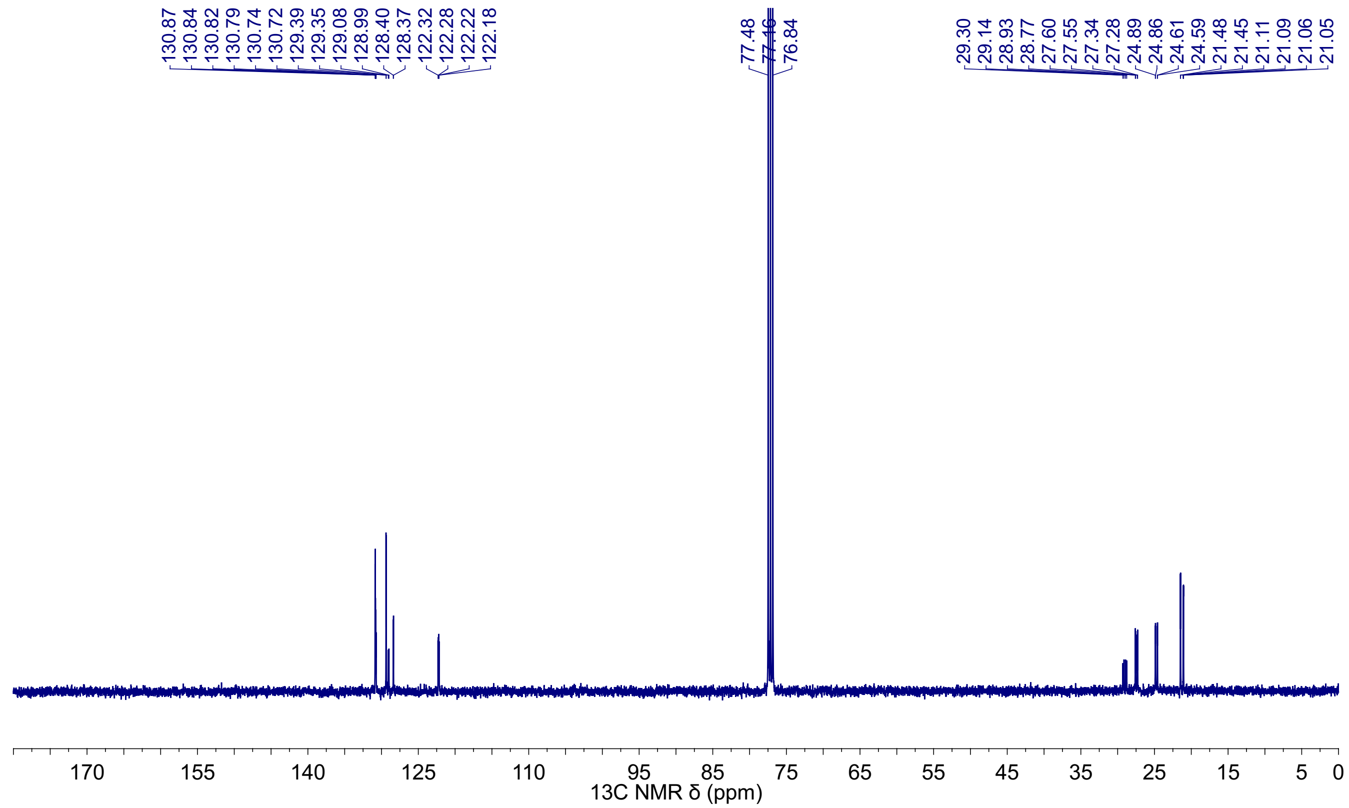

Figure S6 ^{13}C NMR spectrum of [Bn–P_2(dmb)$_2$]Br in $CDCl_3$ at 25 °C

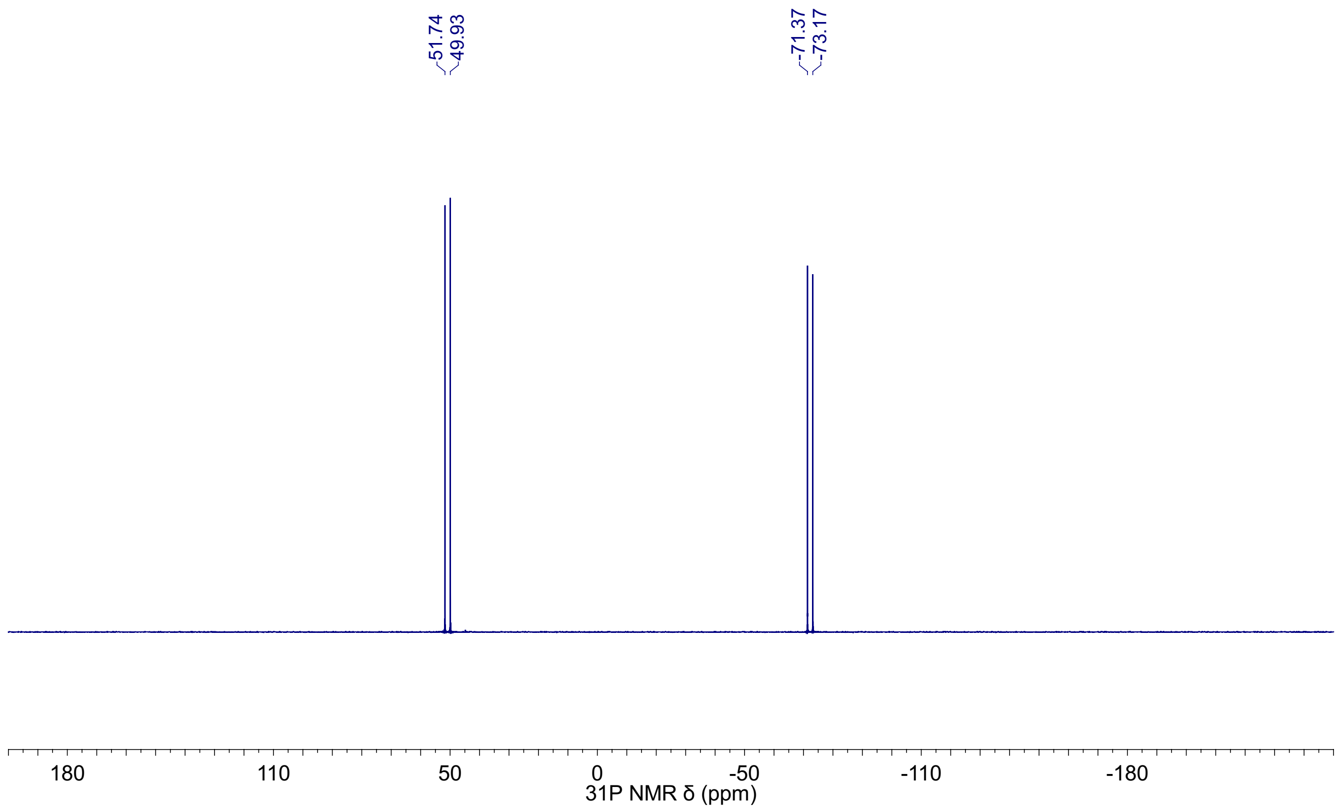

Figure S7 $^{31}P\{^{1}H\}$ NMR spectrum of [Bn–P_2(dmb)$_2$]Br in $CDCl_3$ at 25 °C

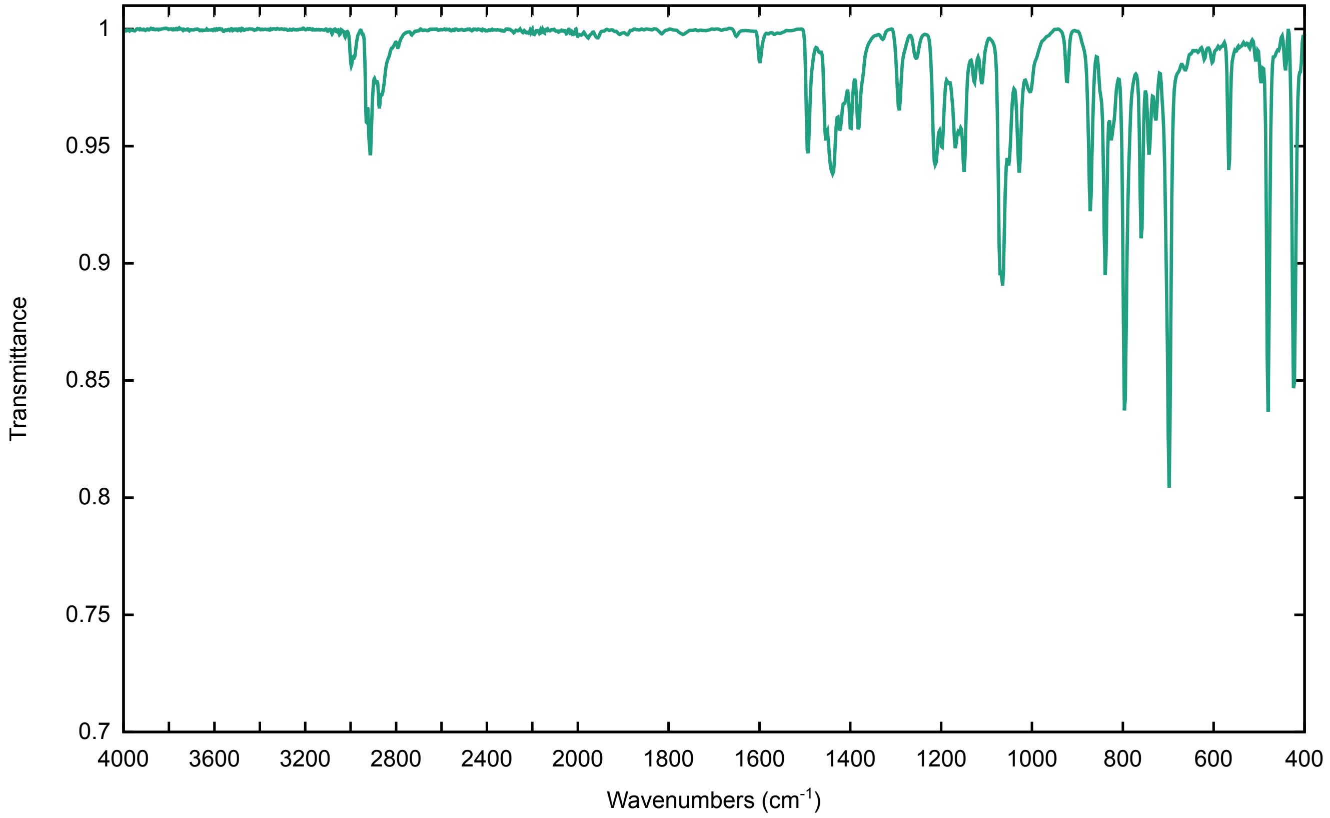

Figure S8 ATR-IR spectrum of [Bn–P_2(dmb)$_2$]Br

5 [^{i}Bu–P_2(dmb)$_2$]Br (4)

5.1 Synthesis of [^{i}Bu–P_2(dmb)$_2$]Br (4)

Inside the glovebox, P_2dmb$_2$ (807 mg, 3.57 mmol, 1 equiv) was dissolved in 15 mL of toluene and transferred to a 100 mL Schelnk tube. A stir bar was added, the tube was sealed and brought outside the glovebox. The tube was then connected to the Schlenk line. Under positive pressure of nitrogen, isobutyl bromide (4 mL, 5.06 g, 36.78 mmol, ca. 10 equiv) was added by syringe. The tube was sealed and heated in an oil bath at 100 °C for 20 hours. After allowing it to cool down to room temperature, the tube was connected to the Schlenk line and then set under vacuum. After removing all volatiles, the tube was sealed and brought back into the glovebox. The solids were dissolved in ca. 10 mL DCM and transferred to a vial. The solvent was removed under vacuum, then the residue triturated with Et_2O (3 x 7 mL). 1.250 g of white powder were obtained (3.44 mmol, 96% yield).

5.2 Characterization of [^{i}Bu–P_2(dmb)$_2$]Br (4)

^{1}H NMR (400 MHz, $CDCl_3$, 25 °C) δ: 3.63 (t, J = 15.1 Hz, 2H), 3.41 (t, J = 14.5 Hz, 2H), 2.87 – 2.59 (m, 6H), 2.23 – 2.05 (m, 1H), 1.89 (s, 6H), 1.81 (d, J = 5.7 Hz, 6H), 1.12 (d, J = 6.6 Hz, 6H) ppm.
^{13}C NMR (101 MHz, $CDCl_3$, 25 °C) δ: 130.84 (dd, J = 12.2, 2.1 Hz), 122.82 (dd, J = 10.4, 4.5 Hz), 31.39 (dd, J = 38.0, 15.0 Hz), 27.59 (dd, J = 26.9, 5.5 Hz), 26.03 (dd, J = 27.6, 2.7 Hz), 25.35 (dd, J = 4.9, 2.5 Hz), 24.53 (dd, J = 9.3, 3.0 Hz), 21.43 (dd, J = 3.5, 0.8 Hz), 21.33 (dd, J = 4.6, 1.3 Hz) ppm.
^{31}P{^{1}H} NMR (162 MHz, $CDCl_3$, 25 °C) δ: 48.02 (d, J = 285.5 Hz), −70.89 (d, J = 285.6 Hz) ppm.
ATR-IR: 2911, 2868, 1446, 1383, 1209, 1167, 1076, 1060, 839, 789, 440, 419 cm^{-1}.
Elemental analysis [%] found (calculated for $C_{16}H_{29}P_2Br$): C 52.77 (52.90), H 7.85 (8.05), N $<$ 0.02 (0).

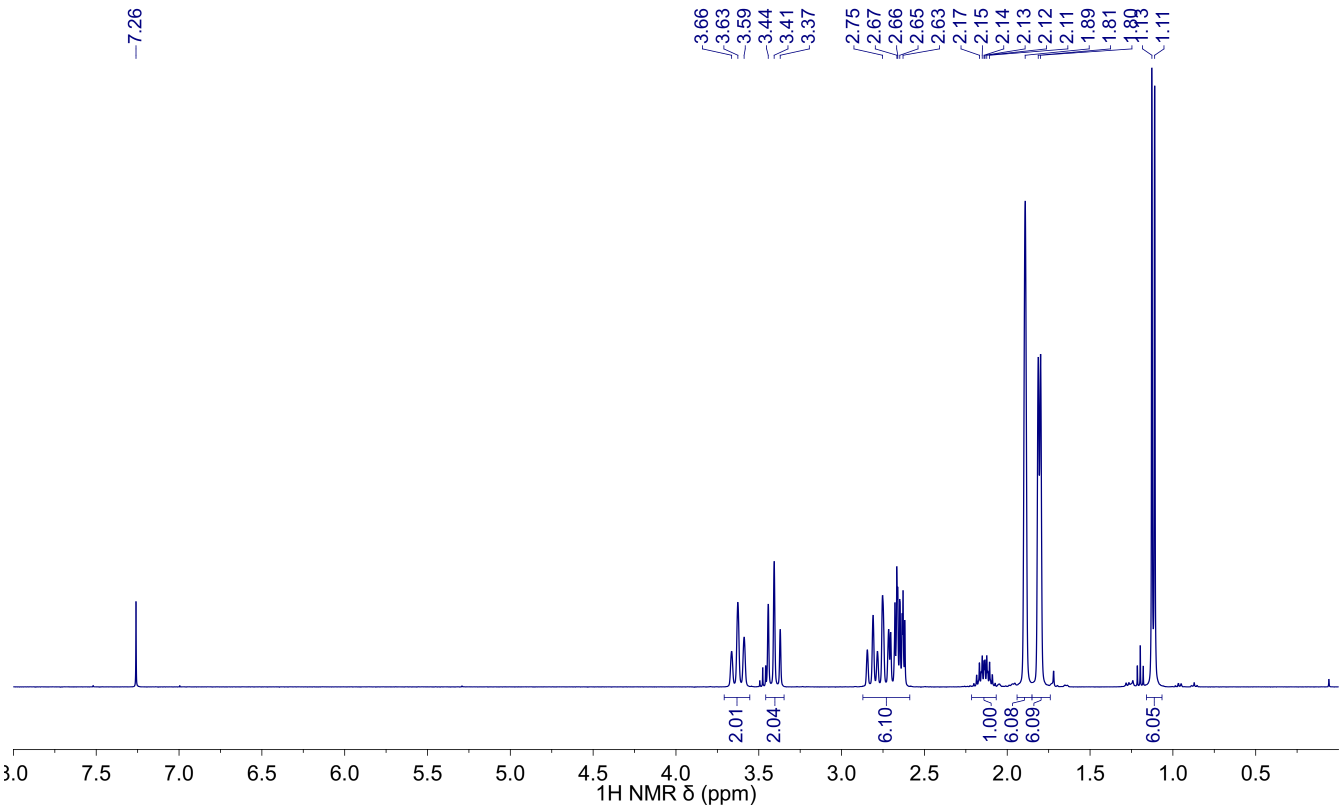

Figure S9 ^{1}H NMR spectrum of [^{i}Bu–P_2(dmb)$_2$]Br in $CDCl_3$ at 25 °C

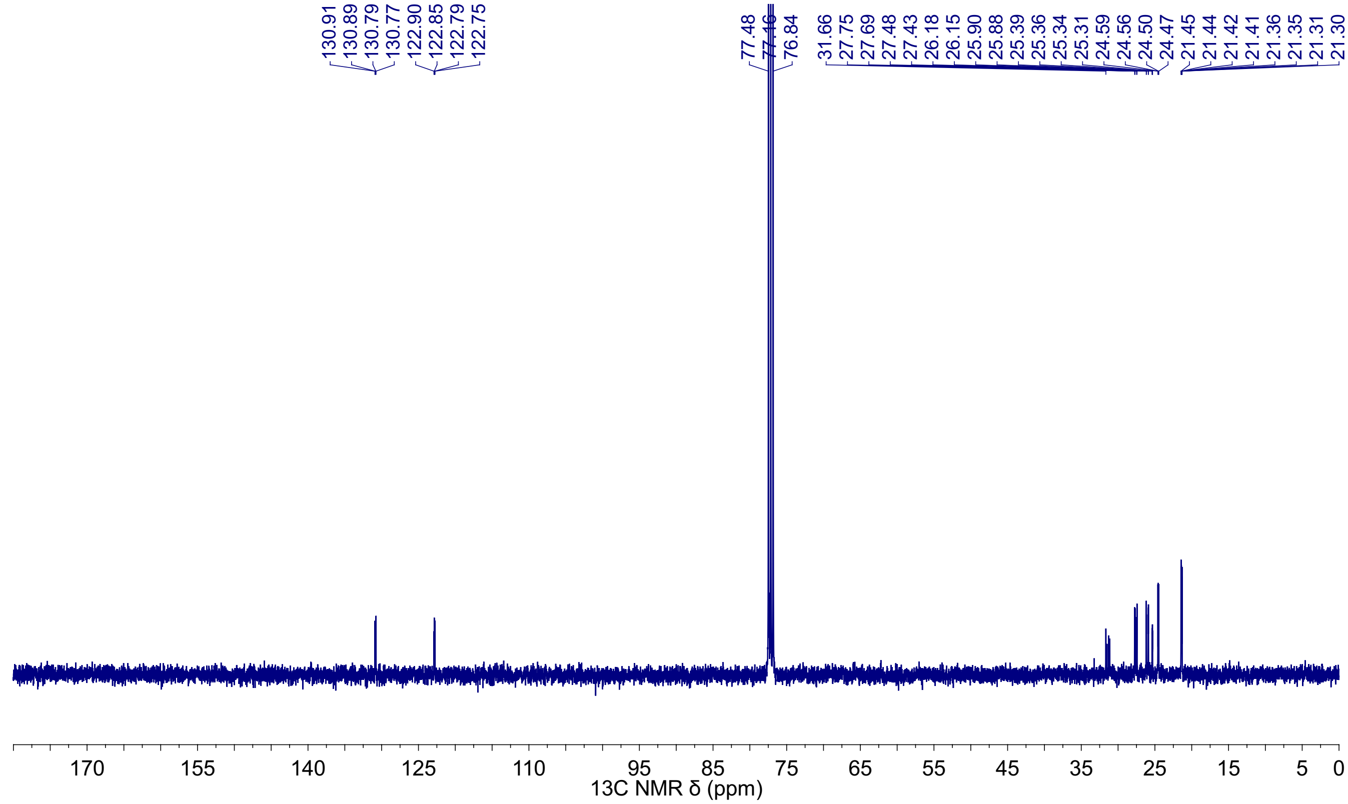

Figure S10 ^{13}C NMR spectrum of [^{i}Bu–P_2(dmb)$_2$]Br in $CDCl_3$ at 25 °C

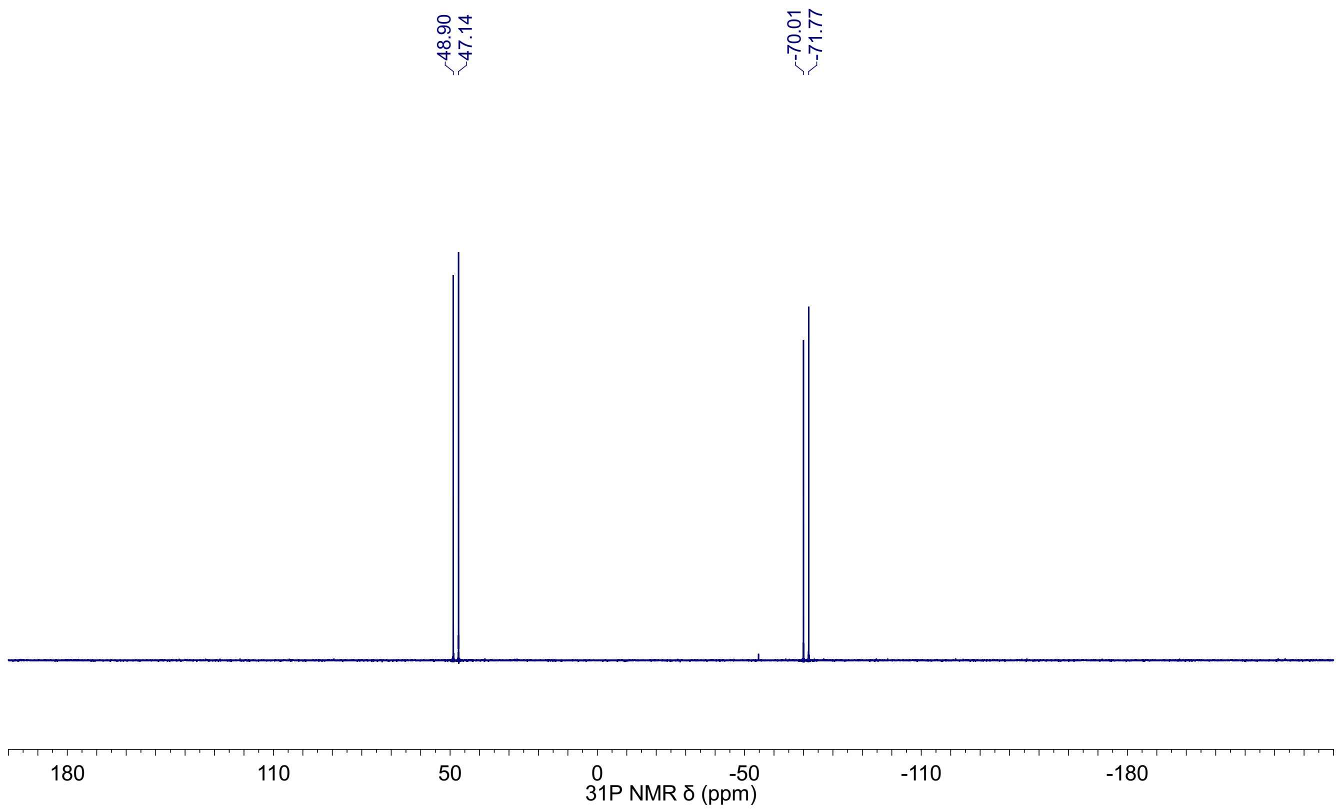

Figure S11 $^{31}P\{^{1}H\}$ NMR spectrum of [^{i}Bu–P_2(dmb)$_2$]Br in $CDCl_3$ at 25 °C

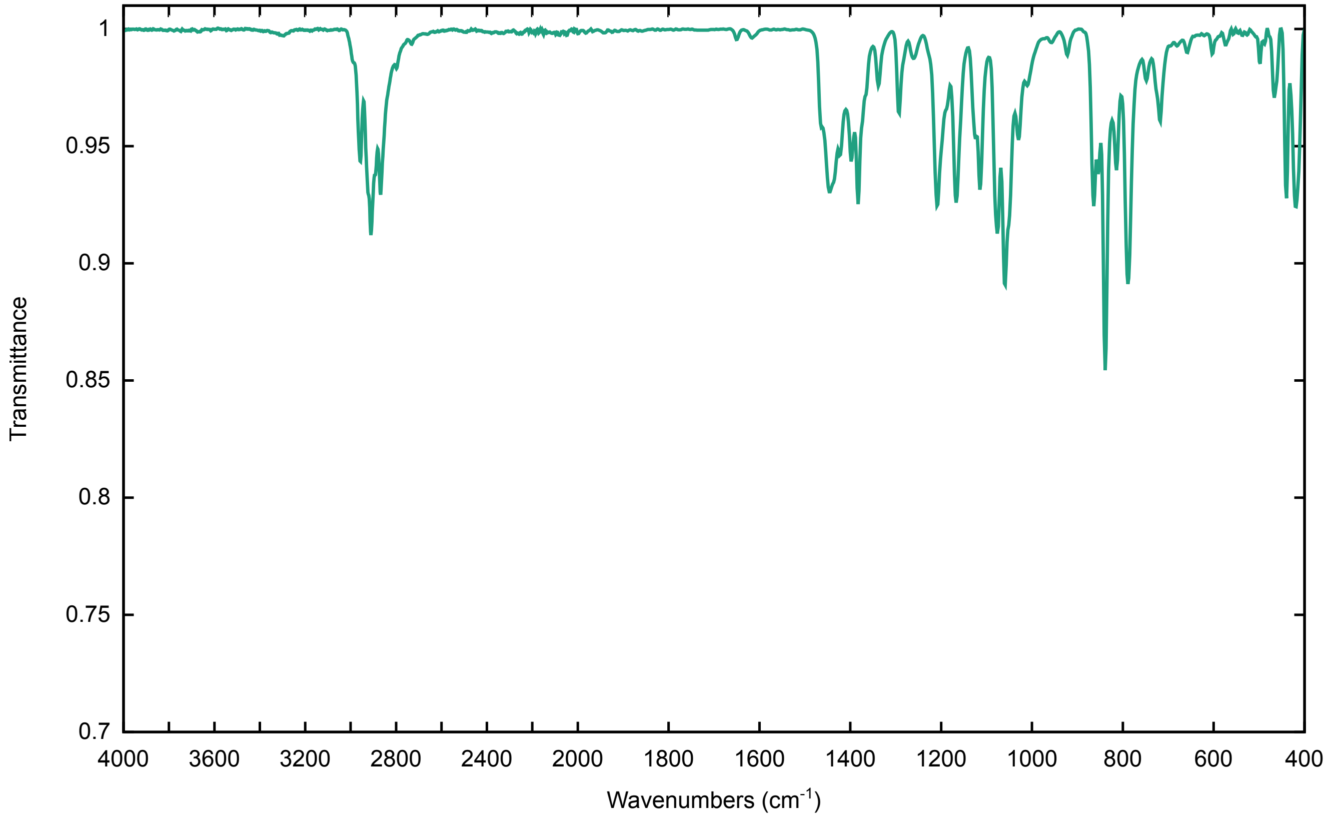

Figure S12 ATR-IR spectrum of [^{i}Bu–P_2(dmb)$_2$]Br

6 Me_2–DPC (5)

6.1 Synthesis of Me_2–DPC (5)

Inside the glovebox, [Me–P_2(dmb)$_2$]I (920 mg, 2.5 mmol, 1 equiv) was slurried in 60 mL of THF in a 200 mL round bottom flask containing a stir bar. To this slurry, methyllithium (2 mL of 1.6 M solution in Et_2O, 3.2 mmol, 1.3 equiv) was added dropwise by syringe. After the addition, the solution became homogeneous and yellow. The mixture was allowed to stir at room temperature for 20 min, after which it was placed under vacuum in order to remove THF. The residue was slurried in 100 mL Et_2O, then filtered through an alumina pad. The flask and alumina were rinsed with an additional 40 mL ether. The clear, colorless, homogeneous filtrate was dried under vacuum and tritured with pentane to obtain 601 mg of off-white powder (2.34 mmol, 94% yield). The product obtained through this synthetic route did not pass elemental analysis and had some impurities in the baseline of its $^{31}P\{^1H\}$ NMR spectrum.

Alternative synthesis: Inside the glovebox, [Me–P_2(dmb)$_2$]I (84 mg, 0.23 mmol, 1 equiv) was slurried in 16 mL of Et_2O in a 20 mL vial containing a stir bar. To this slurry, methyllithium (150 μL of 1.6 M solution in Et_2O, 0.24 mmol, 1.04 equiv) was added dropwise by syringe. Within 5 minutes of the addition, the solution became colorless and homogeneous. The mixture was allowed to stir at room temperature for 4 hours, after which it was filtered through an alumina pad. The alumina was rinsed with an additional 2x20 mL ether. The clear, colorless, homogeneous filtrate was dried under vacuum, then ca. 10mL Et_2O were used to transfer the material into a vial. After drying under vacuum and triturating with pentane, 60 mg of white powder were obtained (0.23 mmol, quantitative yield).

6.2 Characterization of Me_2–DPC (5)

^1H NMR (400 MHz, C_6D_6, 25 °C) δ: 2.84 (dd, J = 12.7, 6.5 Hz, 4H), 1.86 (s, 12H), 1.84 (m, 4H), 0.84 (d, J = 4.5 Hz, 3H) ppm.

^{13}C NMR (101 MHz, C_6D_6, 25 °C) δ: 125.54 (t, J = 5.4 Hz), 39.83 (dd, J = 12.2, 4.6 Hz), 20.36 – 19.25 (m), 13.24 (dt, J = 5.8, 2.6 Hz) ppm. $^{31}P\{^1H\}$ NMR (162 MHz, C_6D_6, 25 °C) δ: −60.94 ppm.

ATR-IR: 2941, 2923, 2890, 2861, 1420, 1381, 1284, 1041, 883, 843, 696 cm^{-1}.

Elemental analysis [%] found (calculated for $C_{14}H_{26}P_2$): C 65.36 (65.61), H 10.14 (10.23), N $<$ 0.02 (0).

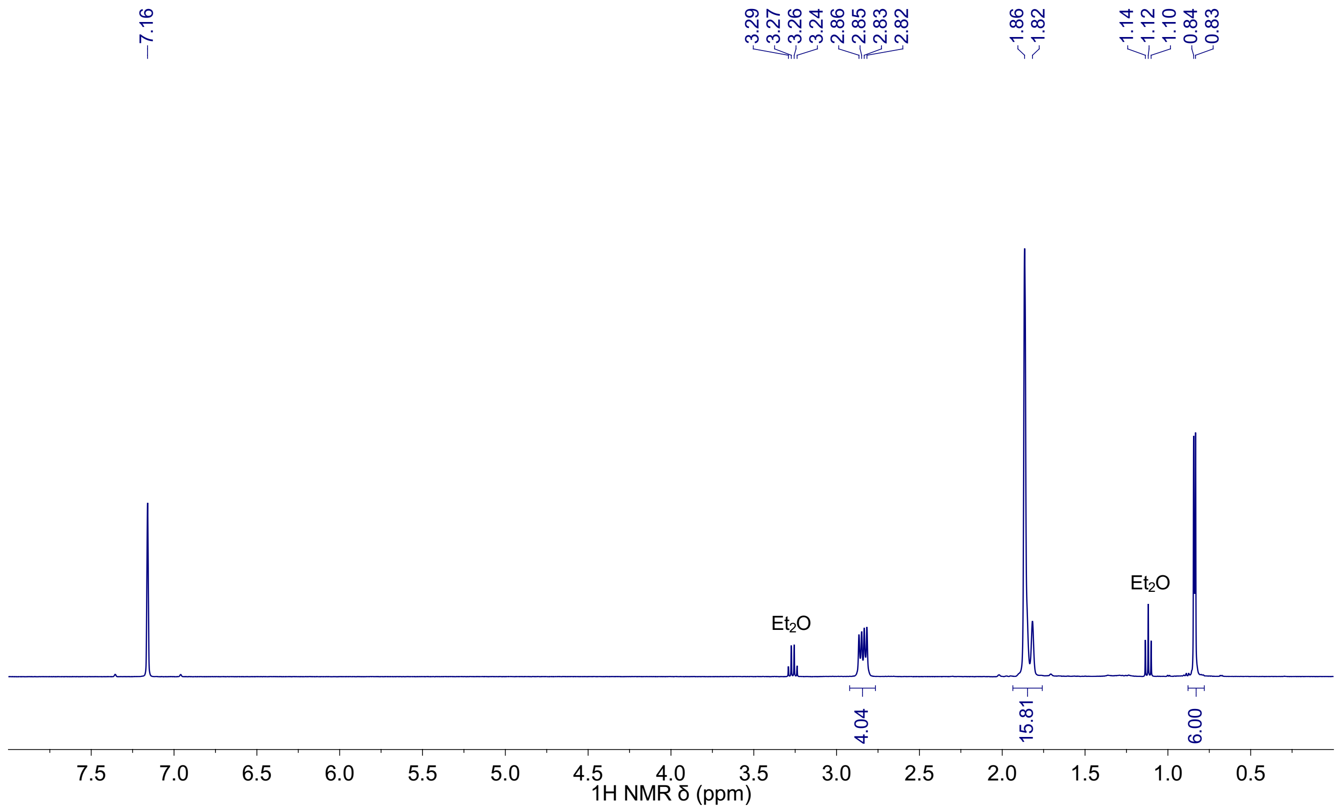

Figure S13 ^{1}H NMR spectrum of Me_2–DPC in C_6D_6 at 25 °C

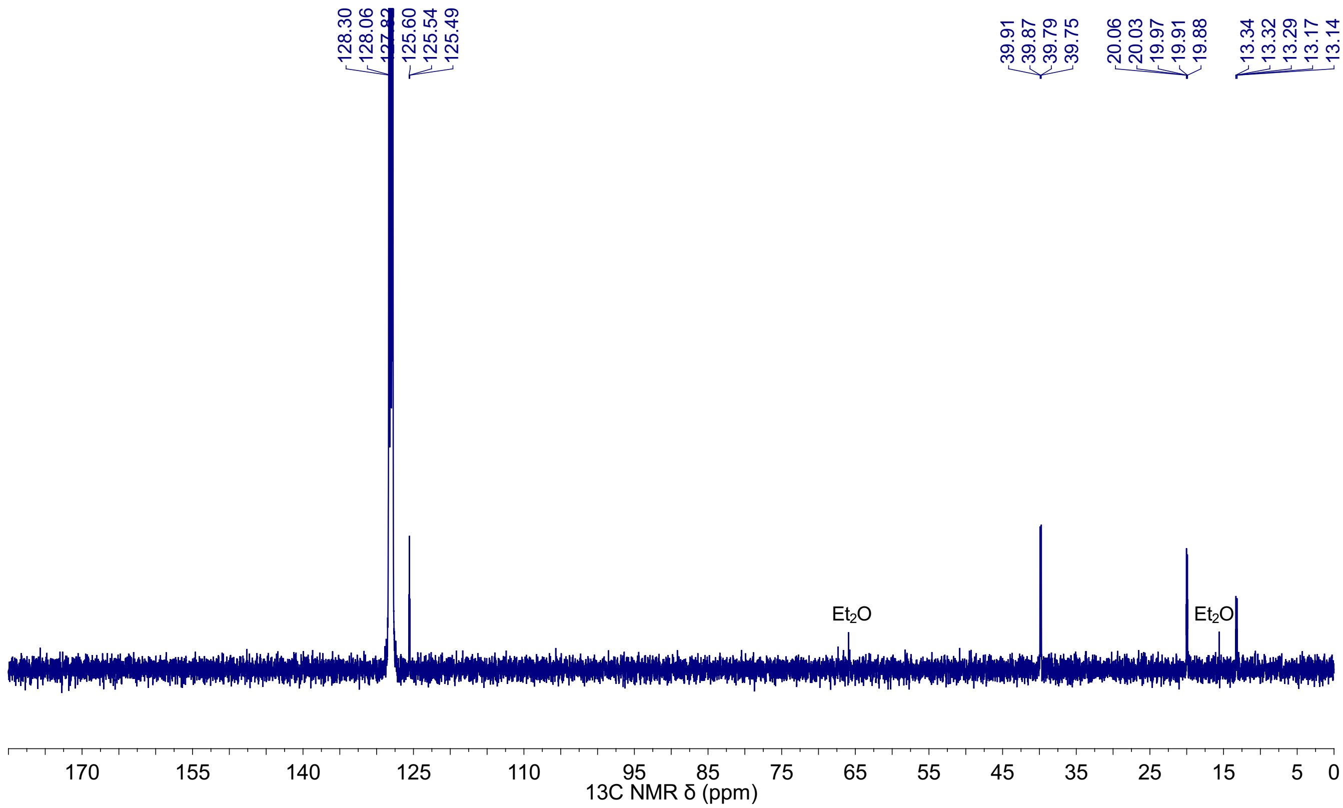

Figure S14 ^{13}C NMR spectrum of Me_2–DPC in C_6D_6 at 25 °C

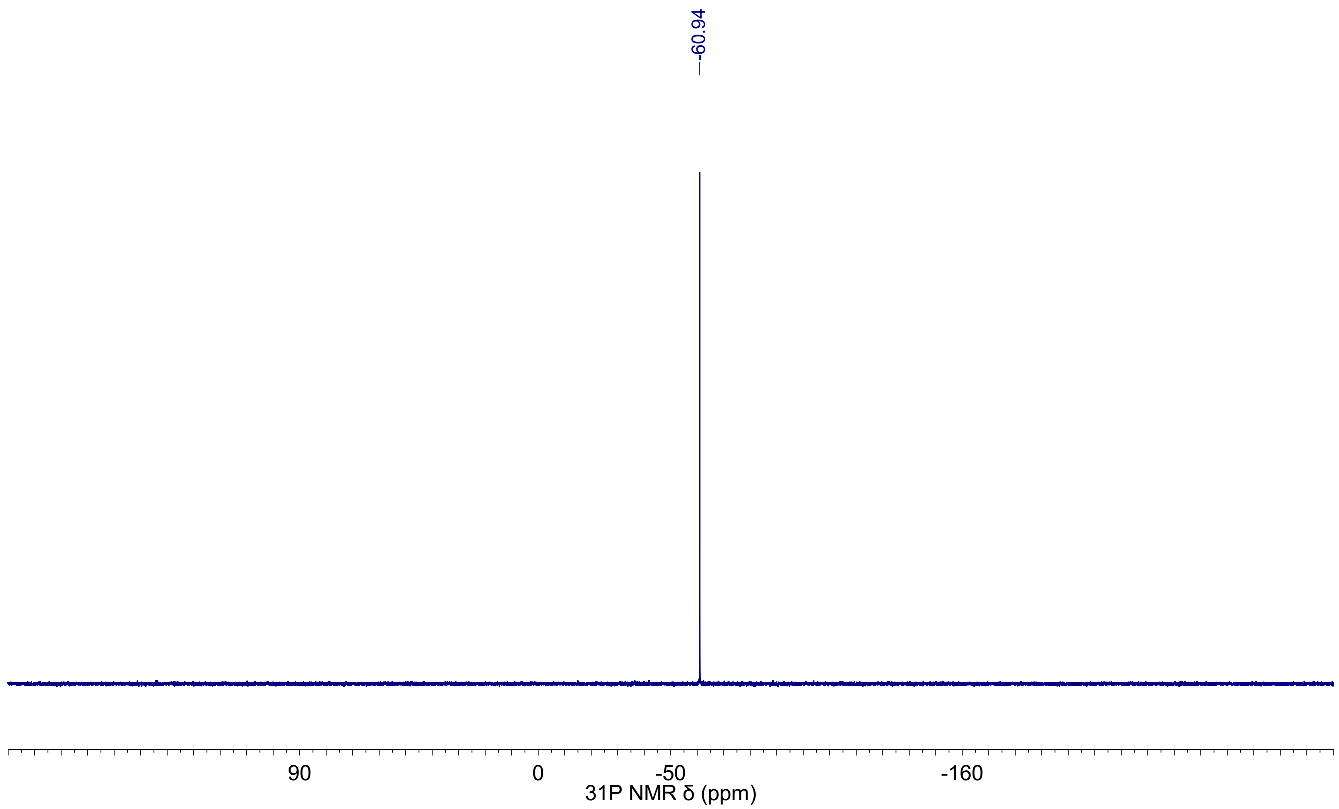

Figure S15 $^{31}P\{^{1}H\}$ NMR spectrum of Me_2–DPC in C_6D_6 at 25 °C

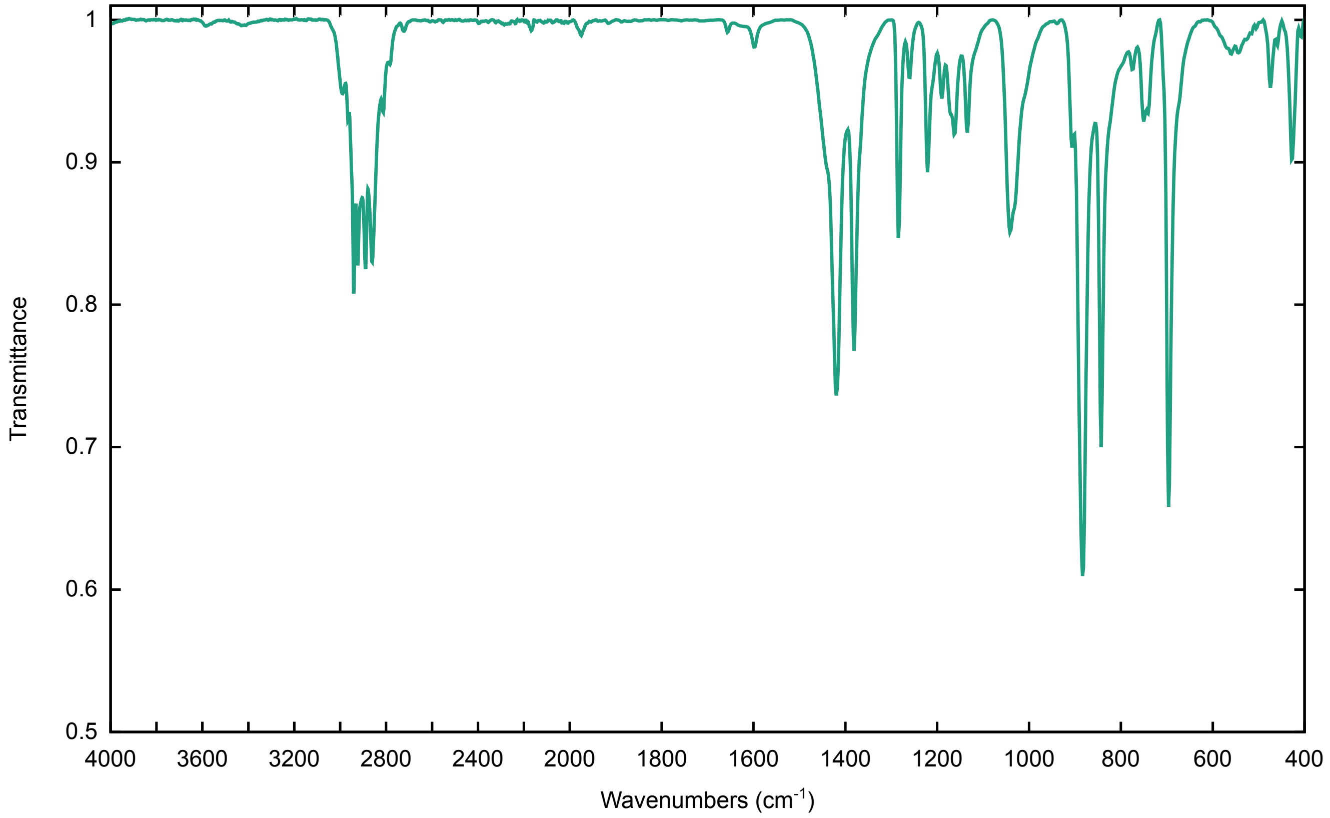

Figure S16 ATR-IR spectrum of Me_2–DPC

7 Me,Cy–DPC (6)

7.1 Synthesis of Me,Cy–DPC (6)

Inside the glovebox, [Me–P_2(dmb)$_2$]I (920 mg, 2.5 mmol, 1 equiv) was slurried in 60 mL of THF in a 200 mL round bottom flask containing a stir bar. To this slurry, cyclohexylmagnesium chloride (4 mL of 1 M solution in 2-Me-THF, 4 mmol, 1.6 equiv) was added dropwise by syringe. After stirring overnight at room temperature, the reaction mixture was still heterogeneous. An aliquot was taken and analyzed by $^{31}P\{^1H\}$ NMR to confirm conversion to the desired product. After a total reaction time of 16 hours, the mixture was placed under vacuum in order to remove THF. The residue was slurried in 100 mL Et_2O, then filtered through an alumina pad. The flask and alumina were rinsed with an additional 40 mL ether. The clear homogeneous filtrate was dried under vacuum, then ca. 10mL ether were used to transfer the material into a vial. After drying under vacuum and triturating with pentane, 729 mg of white powder were obtained (2.25 mmol, 90% yield).

7.2 Characterization of Me,Cy–DPC (6)

^1H NMR (400 MHz, C_6D_6, 25 °C) δ: 3.00 (dd, J = 12.6, 6.4 Hz, 2H), 2.90 (dd, J = 13.9, 7.2 Hz, 2H), 1.90 (m, 4H), 1.89 (s, 12H), 1.81 – 1.60 (m, 5H), 1.28 – 1.09 (m, 6H), 0.88 (d, J = 4.7 Hz, 3H) ppm.

^{13}C NMR (101 MHz, C_6D_6, 25 °C) δ: 125.54 (dt, J = 6.8, 5.6 Hz), 39.96 (dd, J = 15.0, 1.4 Hz), 37.27 (d, J = 12.6 Hz), 34.16 (dd, J = 18.9, 1.5 Hz), 29.72 (d, J = 12.3 Hz), 27.53 (d, J = 9.3 Hz), 26.94 (d, J = 0.5 Hz), 20.17 (d, J = 4.8 Hz), 20.03 (d, J = 4.9 Hz), 13.39 (d, J = 15.0 Hz) ppm.

$^{31}P\{^1H\}$ NMR (162 MHz, C_6D_6, 25 °C) δ: −38.94 (d, J = 5.1 Hz), −60.78 (d, J = 5.5 Hz) ppm.

ATR-IR: 2923, 2849, 1448, 1424, 1378, 882, 846, 696 cm^{-1}.

Elemental analysis [%] found (calculated for $C_{19}H_{34}P_2$): C 70.20 (70.34), H 10.51 (10.56), N $<$ 0.02 (0).

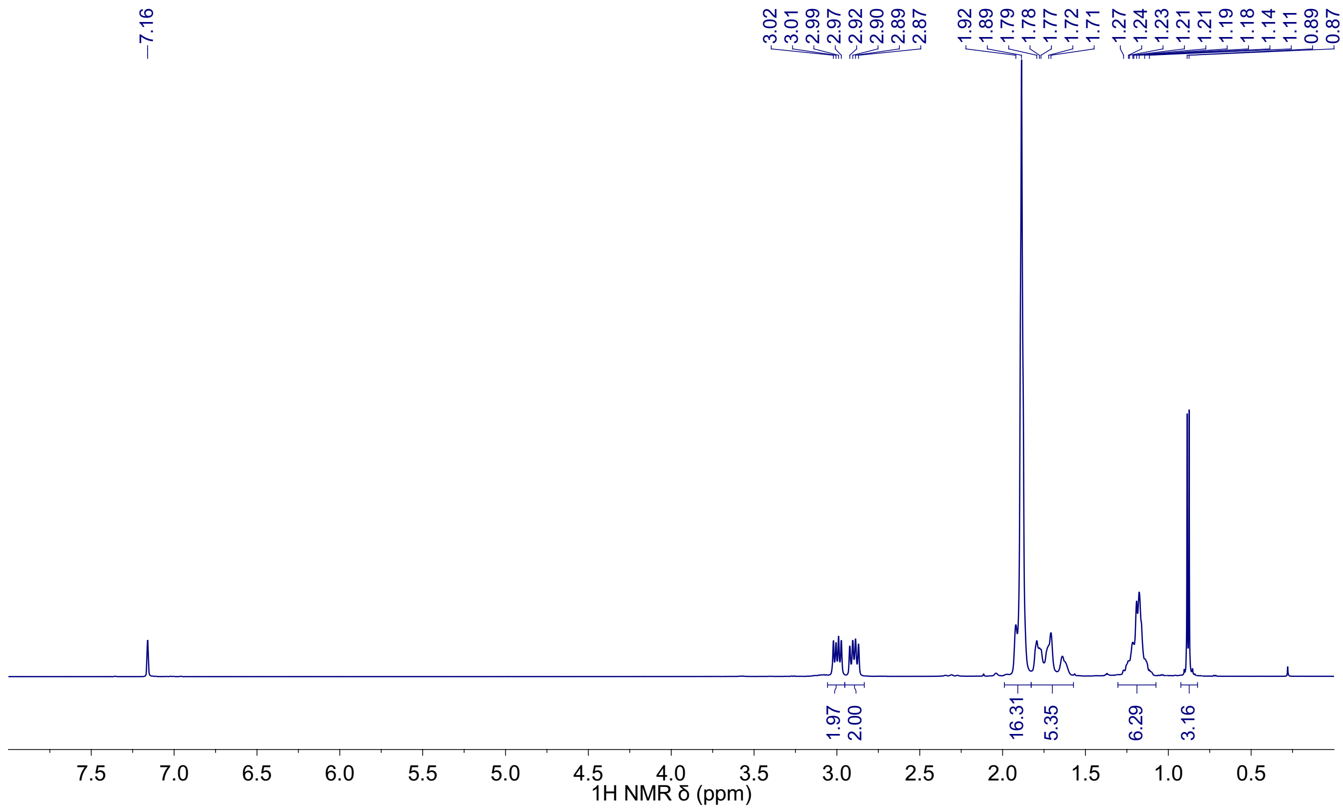

Figure S17 ^{1}H NMR spectrum of Me,Cy–DPC in C_6D_6 at 25 °C

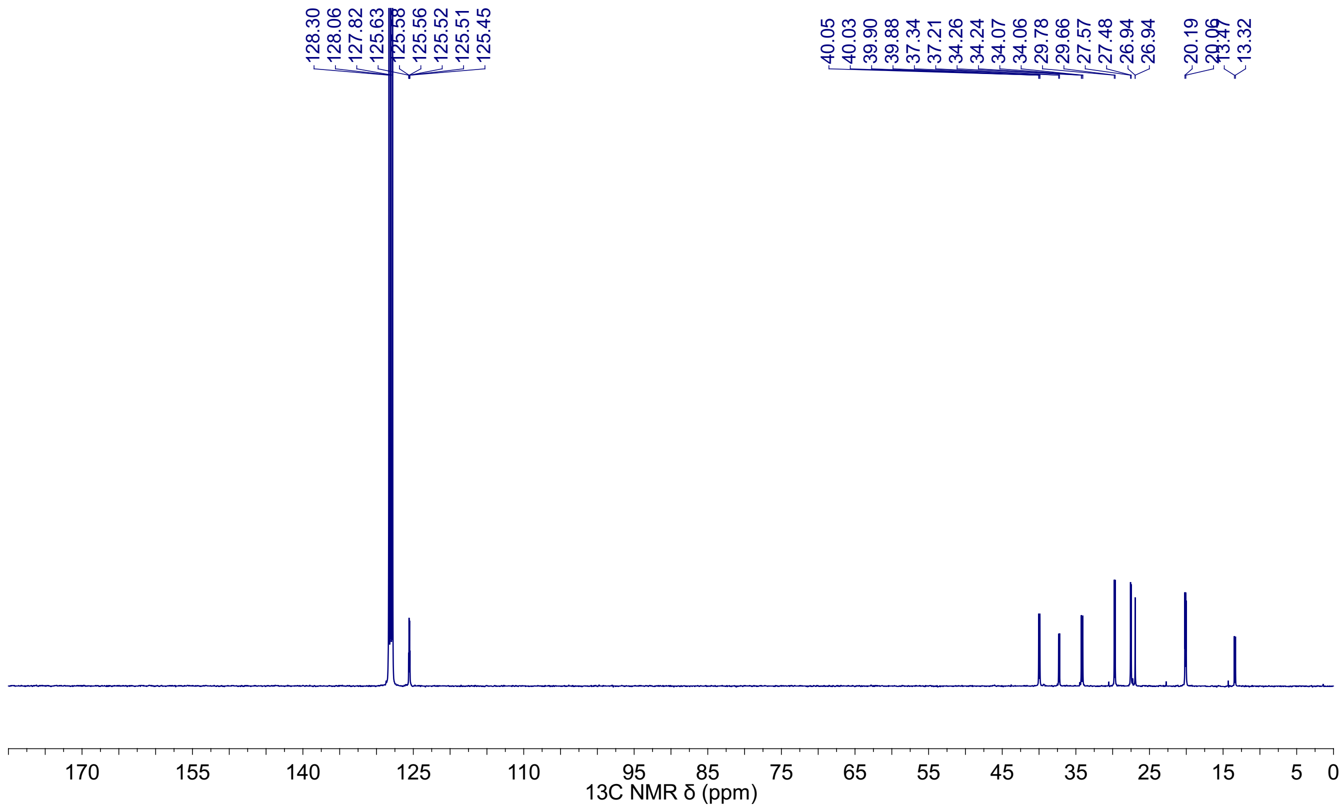

Figure S18 ^{13}C NMR spectrum of Me,Cy–DPC in C_6D_6 at 25 °C

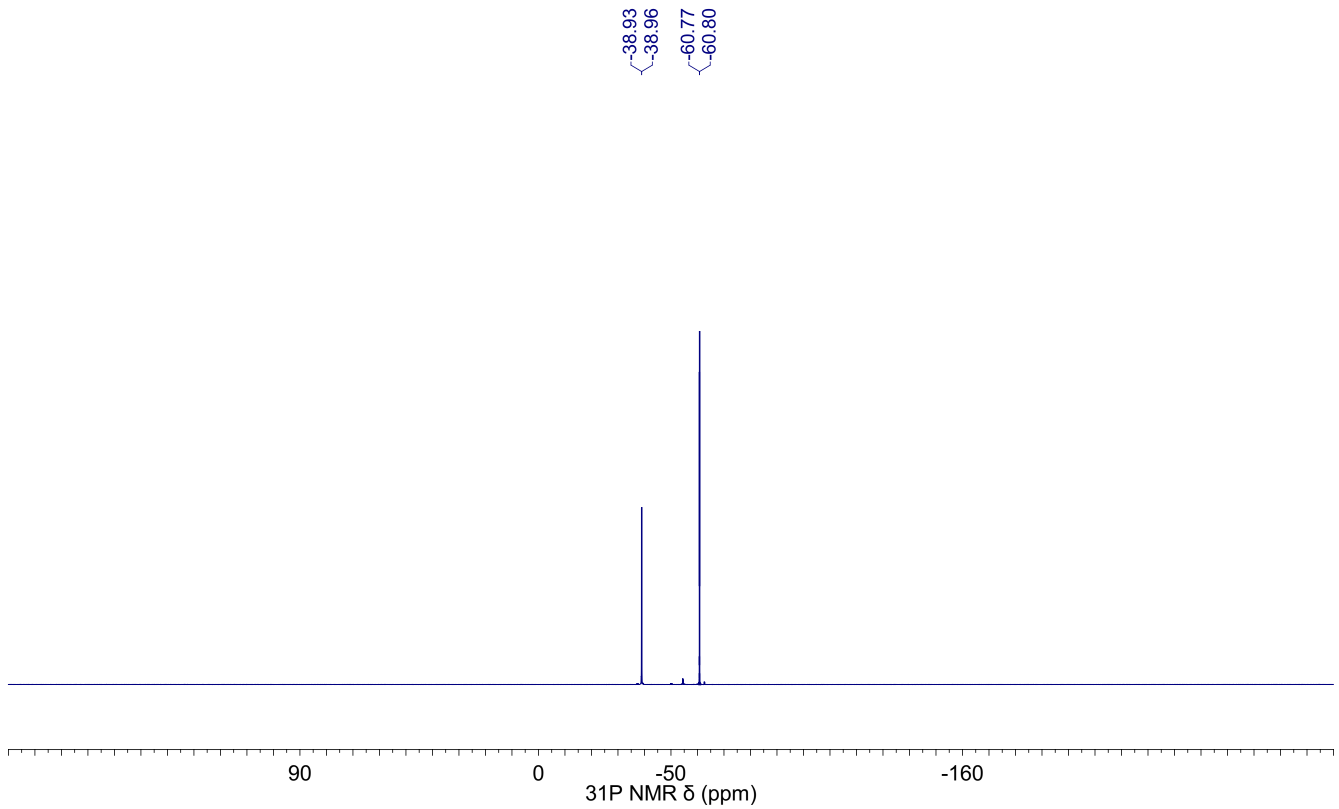

Figure S19 $^{31}P\{^{1}H\}$ NMR spectrum of Me,Cy–DPC in C_6D_6 at 25 °C

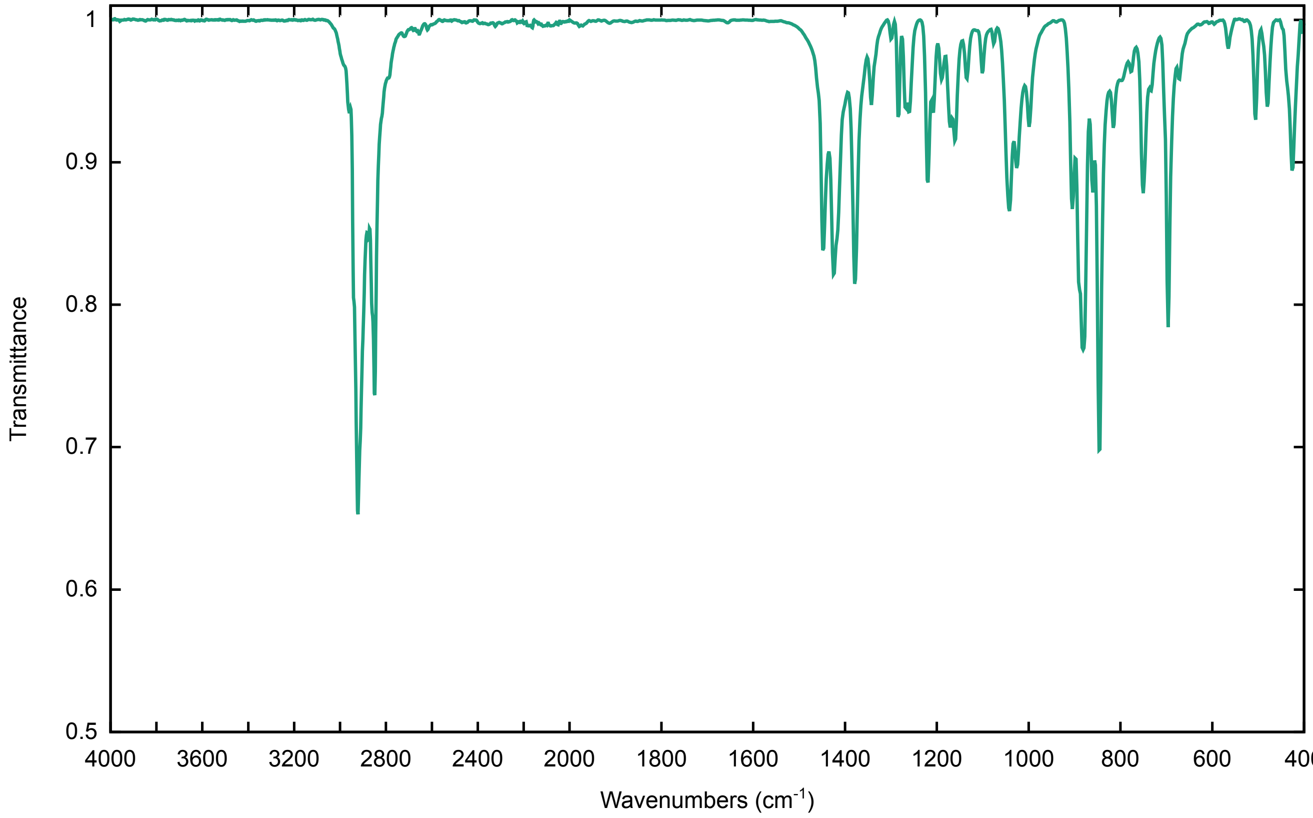

Figure S20 ATR-IR spectrum of Me,Cy–DPC

8 ^{i}Bu,Cy–DPC (7)

8.1 Synthesis of ^{i}Bu,Cy–DPC (7)

Inside the glovebox, [^{i}Bu–P_2(dmb)$_2$]Br (1.090 g, 3.0 mmol, 1 equiv) was slurried in 40 mL of THF in a 200 mL round bottom flask containing a stir bar. To this slurry, cyclohexylmagnesium chloride (3.3 mL of 1 M solution in 2-Me-THF, 3.3 mmol, 1.1 equiv) was added dropwise by syringe. Most solids had dissolved after the addition was over. The reaction flask was capped with a septum and the reaction mixture was allowed to stir at room temperature. After 1 hour, the homogeneous solution was placed under vacuum in order to remove THF. The residue was slurried in 100 mL Et_2O, then filtered through an alumina pad. The flask and alumina were rinsed with an additional 40 mL ether. The filtrate was dried under vacuum, then ca. 10 mL pentane was used to transfer the material to a vial. After drying the solution under vacuum, 1.014 g of white powder were obtained (2.77 mmol, 92% yield).

8.2 Characterization of ^{i}Bu,Cy–DPC (7)

^{1}H NMR (400 MHz, C_6D_6, 25 °C) δ: 3.03 (dd, J = 12.8, 6.4 Hz, 2H), 2.92 (dd, J = 13.0, 6.7 Hz, 2H), 1.94 (d, J = 13.3 Hz, 4H), 1.90 (s, 12H), 1.86 – 1.53 (m, 6H), 1.32 – 1.12 (m, 8H), 1.05 (d, J = 6.6 Hz, 6H) ppm.

^{13}C NMR (101 MHz, C_6D_6, 25 °C) δ: 125.53 (dd, J = 8.0, 3.3 Hz), 40.29 (d, J = 14.3 Hz), 38.40 (dd, J = 16.4, 1.3 Hz), 37.37 (d, J = 12.6 Hz), 34.18 (dd, J = 18.8, 1.3 Hz), 29.75 (d, J = 12.3 Hz), 27.54 (d, J = 9.3 Hz), 26.95 (s), 26.71 (d, J = 13.8 Hz), 24.59 (d, J = 9.1 Hz), 20.20 (d, J = 3.3 Hz), 20.07 (d, J = 3.4 Hz) ppm.

^{31}P{^{1}H} NMR (162 MHz, C_6D_6, 25 °C) δ: −38.87 (d, J = 4.9 Hz), −54.58 (d, J = 5.3 Hz) ppm.

ATR-IR: 2947, 2921, 2849, 1445, 1380, 856, 849 cm^{-1}.

Elemental analysis [%] found (calculated for $C_{22}H_{40}P_2$): C 71.83 (72.10), H 10.83 (11.00), N $<$ 0.02 (0).

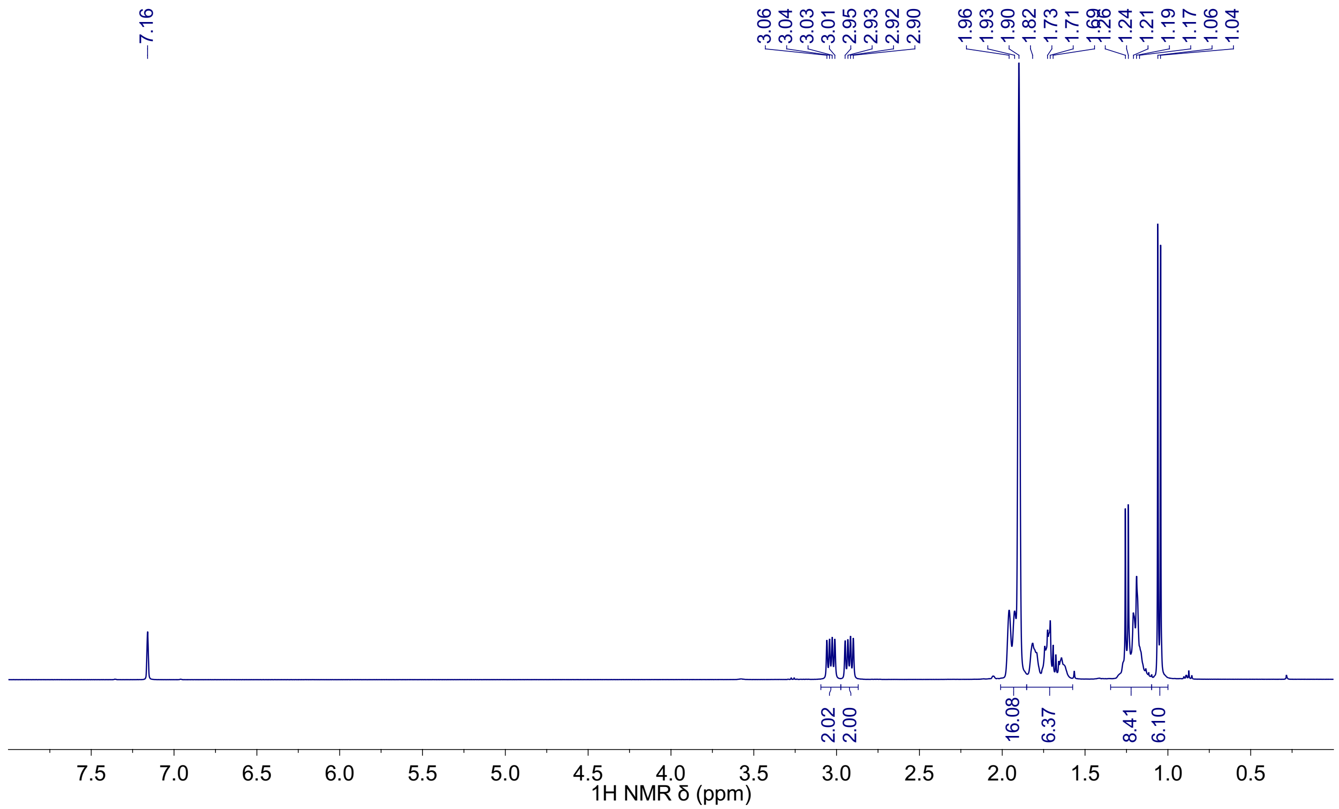

Figure S21 ^{1}H NMR spectrum of ^{i}Bu,Cy–DPC in C_6D_6 at 25 °C

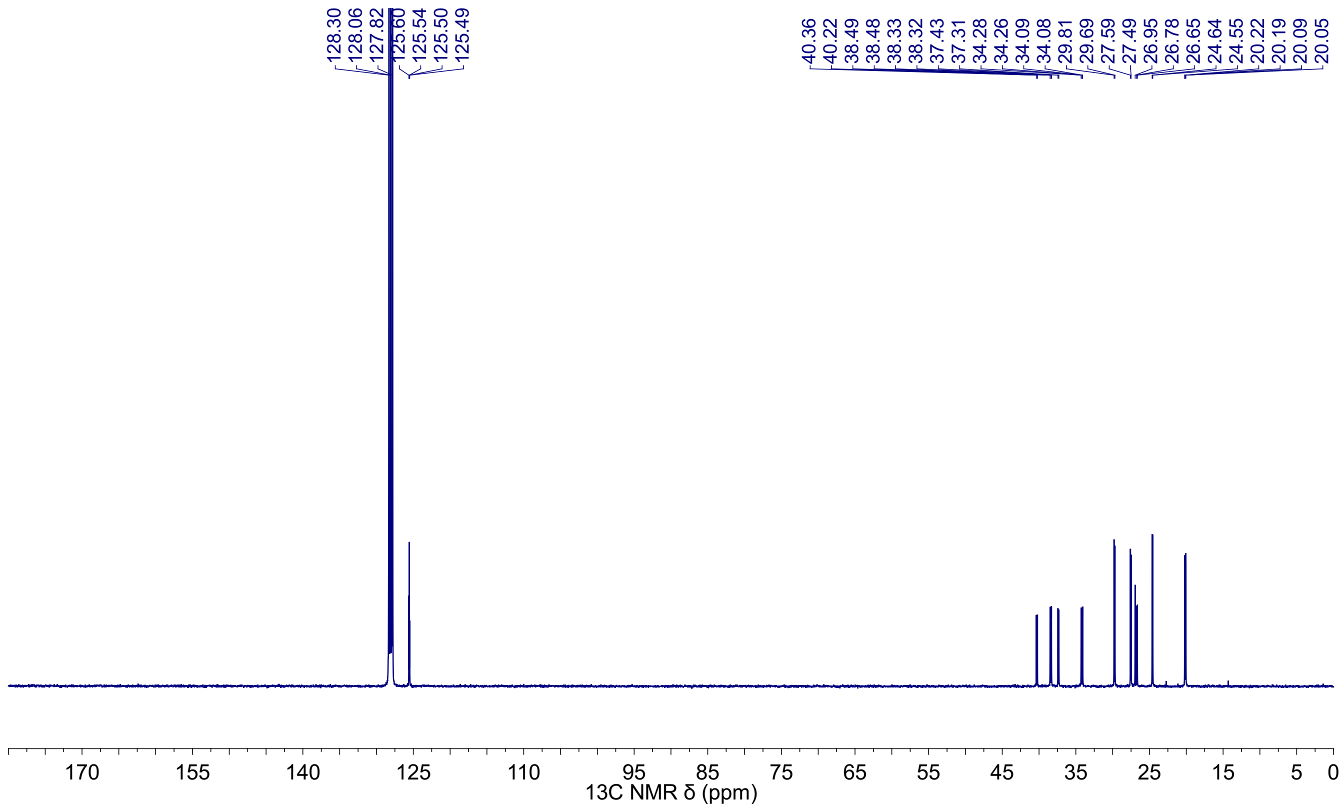

Figure S22 ^{13}C NMR spectrum of ^{i}Bu,Cy–DPC in C_6D_6 at 25 °C

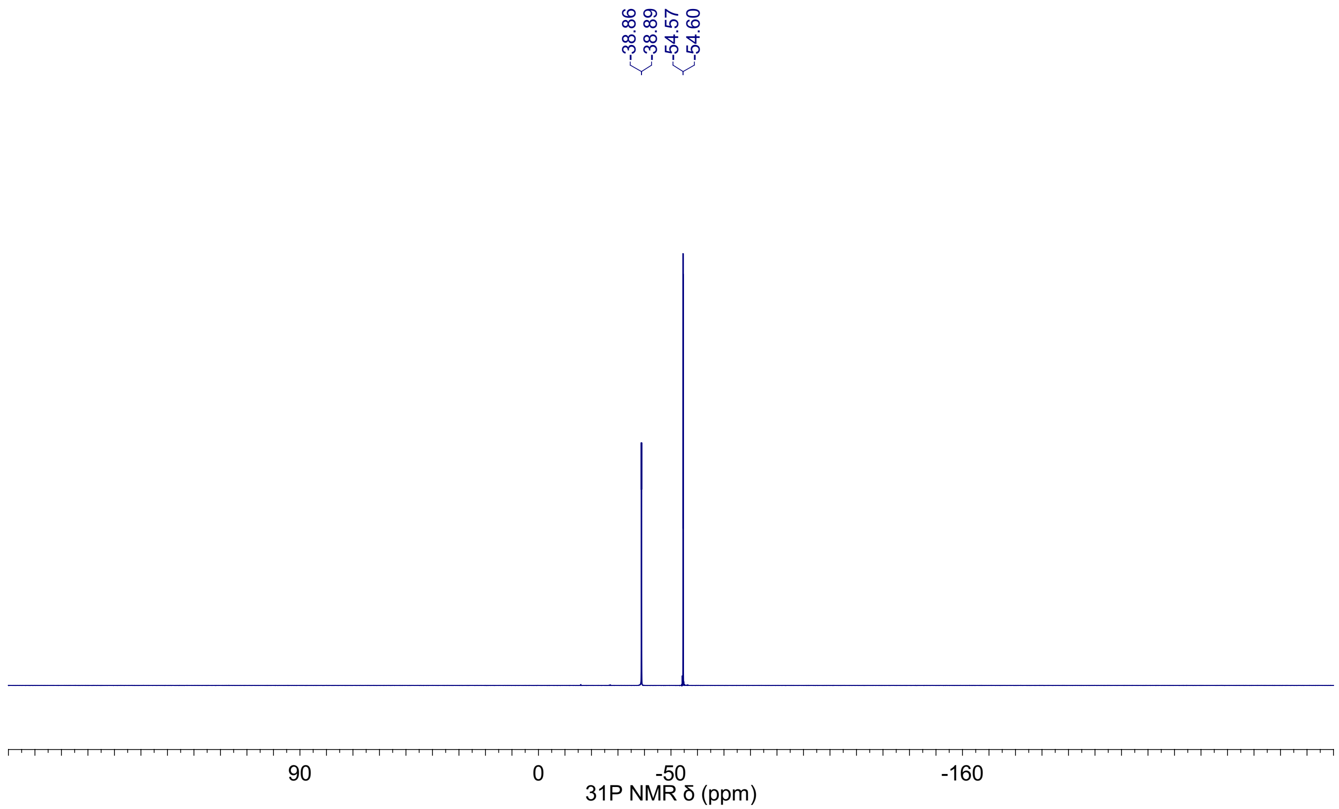

Figure S23 $^{31}P\{^{1}H\}$ NMR spectrum of ^{i}Bu,Cy–DPC in C_6D_6 at 25 °C

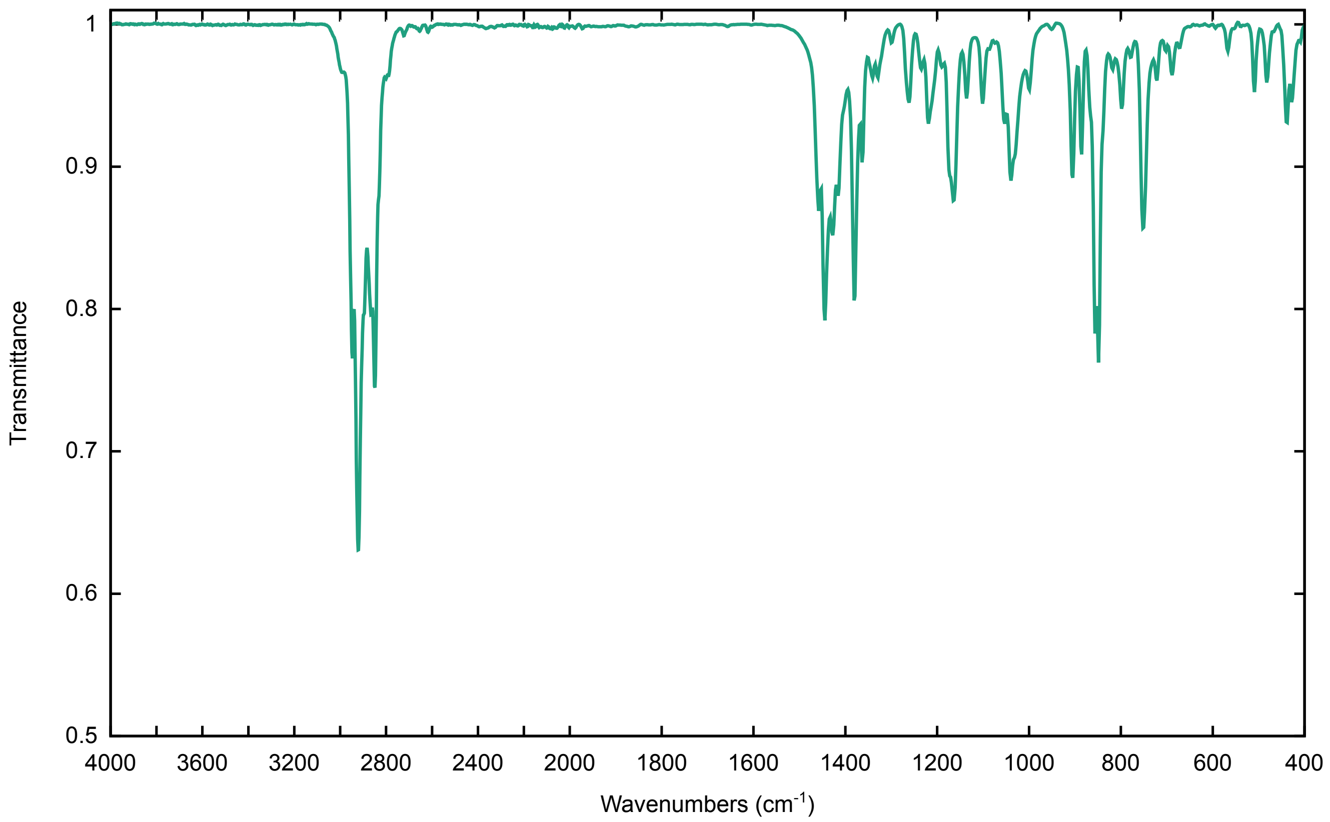

Figure S24 ATR-IR spectrum of ^{i}Bu,Cy–DPC

9 Bn,Cy–DPC (8)

9.1 Synthesis of Bn,Cy–DPC (8)

Inside the glovebox, [Bn–P_2(dmb)$_2$]Br (1.00 g, 2.52 mmol, 1 equiv) was slurried in 40 mL of THF in a 200 mL round bottom flask containing a stir bar. To this slurry, cyclohexylmagnesium chloride (2.8 mL of 1 M solution in 2-Me-THF, 2.8 mmol, 1.1 equiv) was added dropwise by syringe. The reaction flask was capped with a septum and the reaction mixture was allowed to stir at room temperature. After ca. 30 min, most solids had dissolved. After 1.5 hours, the homogeneous solution was placed under vacuum in order to remove THF. The residue was slurried in 100 mL Et_2O, then filtered through an alumina pad. The flask and alumina were rinsed with an additional 40 mL ether. The clear, homogeneous filtrate was dried under vacuum, then ca. 10 mL pentane was used to transfer the material to a vial. After drying the solution under vacuum, an oily residue was obtained. This was stored overnight in the freezer, after which it solidified and could be broken up into a powder using a spatula. The solids were further dried under vacuum to obtain 939 mg of off-white powder (2.34 mmol, 93% yield).

9.2 Characterization of Bn,Cy–DPC (8)

^{1}H NMR (400 MHz, C_6D_6, 25 °C) δ: 7.23 – 7.12 (m, 4H), 7.10 – 7.00 (m, 1H), 3.08 – 2.81 (m, 4H), 2.63 (d, J = 2.7 Hz, 2H), 2.06 – 1.83 (m, 10H), 1.83 – 1.52 (m, 11H), 1.28 – 1.08 (m, 6H) ppm.

^{13}C NMR (101 MHz, C_6D_6, 25 °C) δ: 138.71 (d, J = 4.6 Hz), 129.57 (d, J = 5.4 Hz), 128.65 (d, J = 0.9 Hz), 126.05 (d, J = 2.0 Hz), 125.88 (t, J = 5.6 Hz), 125.10 (t, J = 5.5 Hz), 37.32 (d, J = 12.7 Hz), 37.06 – 36.61 (m), 34.04 (dd, J = 19.0, 1.3 Hz), 29.70 (d, J = 12.1 Hz), 27.56 (d, J = 9.3 Hz), 26.96 (s), 20.07 (d, J = 13.0 Hz) ppm.

^{31}P{^{1}H} NMR (162 MHz, C_6D_6, 25 °C) δ: −38.92 (d, J = 5.0 Hz), −44.71 (d, J = 5.4 Hz) ppm.

ATR-IR: 2917, 2848, 1446, 856, 771, 698, 477 cm^{-1}.

Elemental analysis [%] found (calculated for $C_{25}H_{38}P_2$): C 74.88 (74.97), H 9.52 (9.56), N $<$ 0.02 (0).

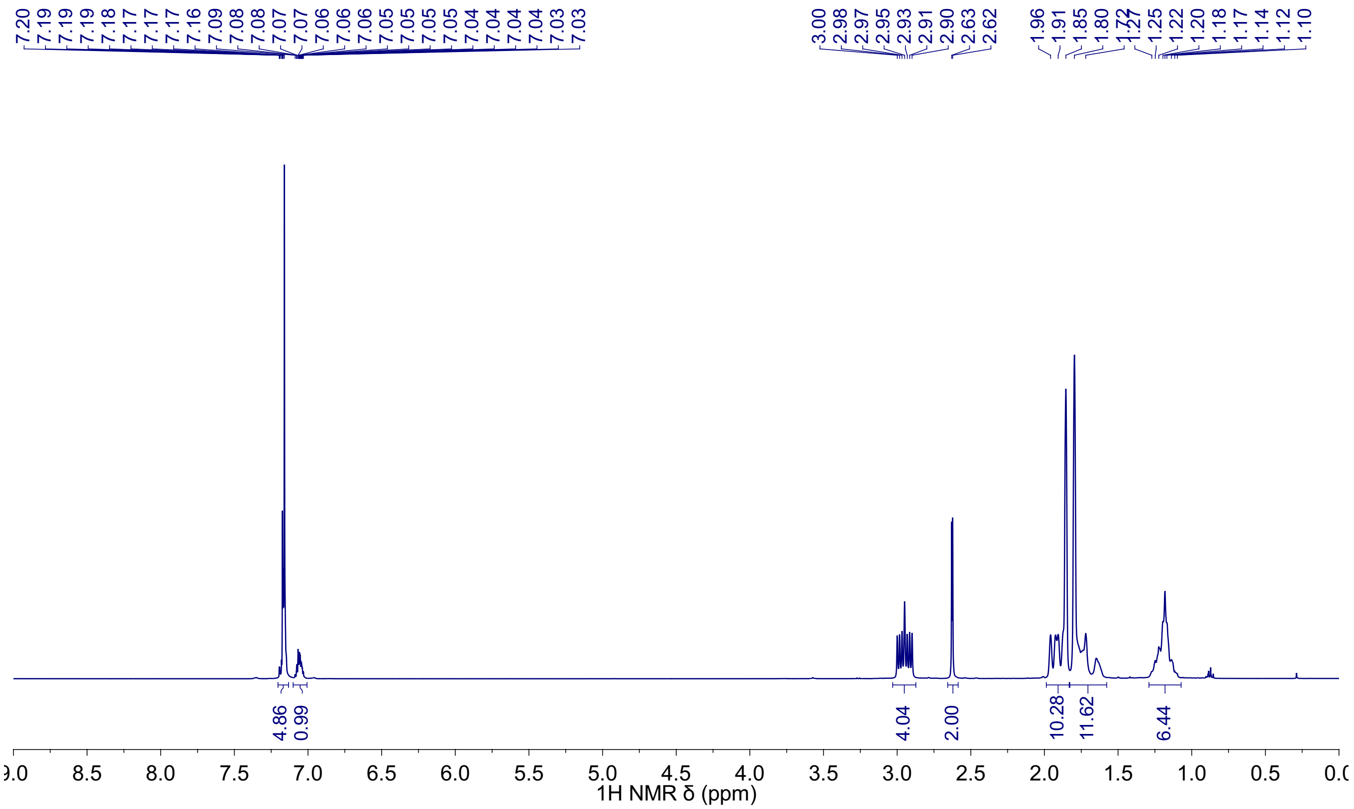

Figure S25 ^{1}H NMR spectrum of Bn,Cy–DPC in C_6D_6 at 25 °C

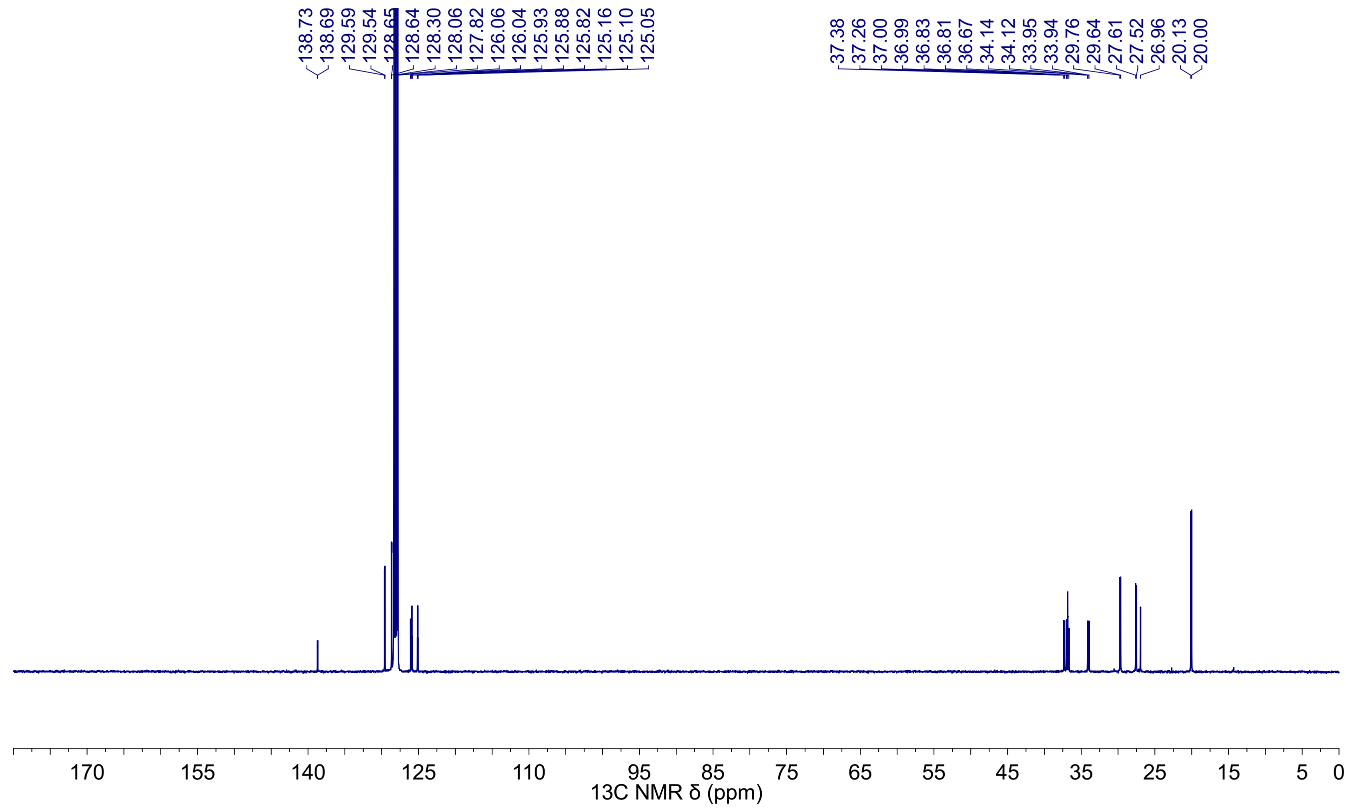

Figure S26 ^{13}C NMR spectrum of Bn,Cy–DPC in C_6D_6 at 25 °C

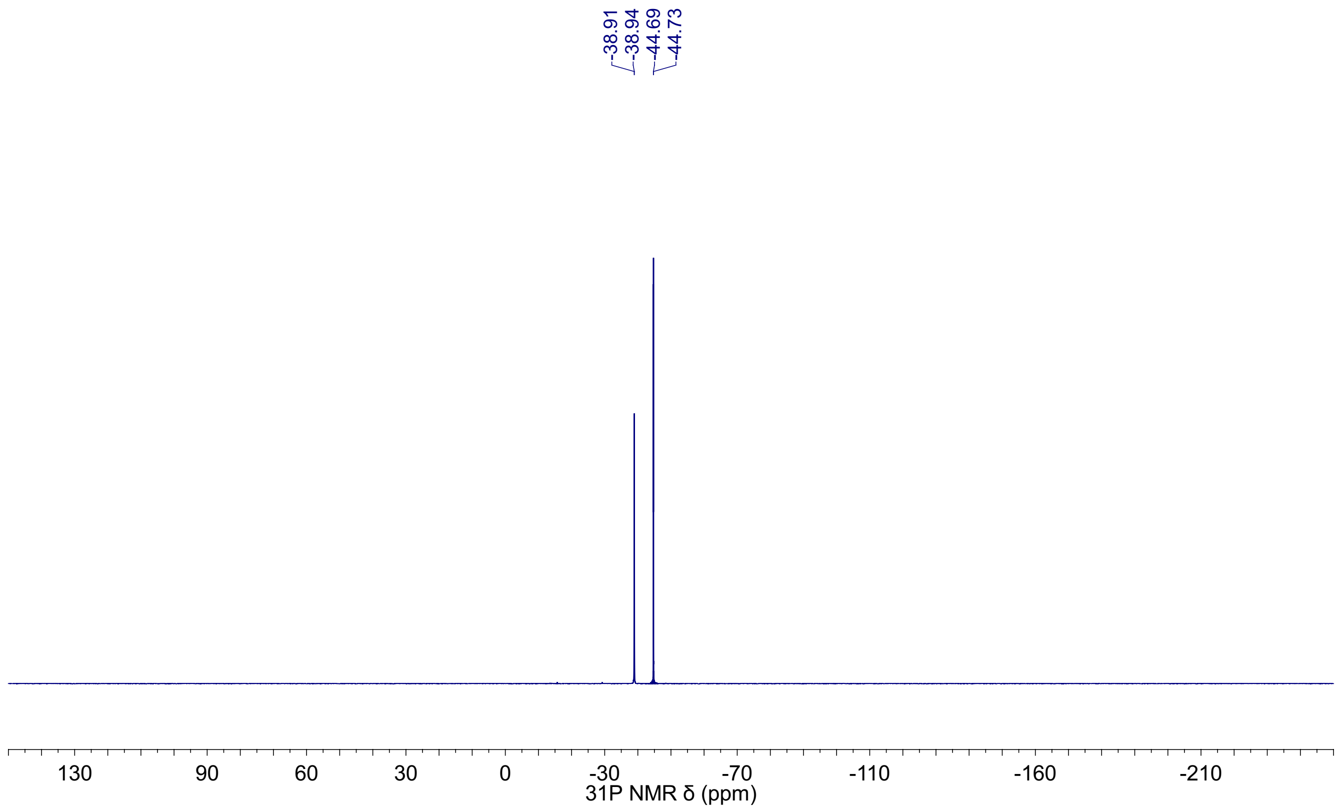

Figure S27 $^{31}P\{^{1}H\}$ NMR spectrum of Bn,Cy–DPC in C_6D_6 at 25 °C

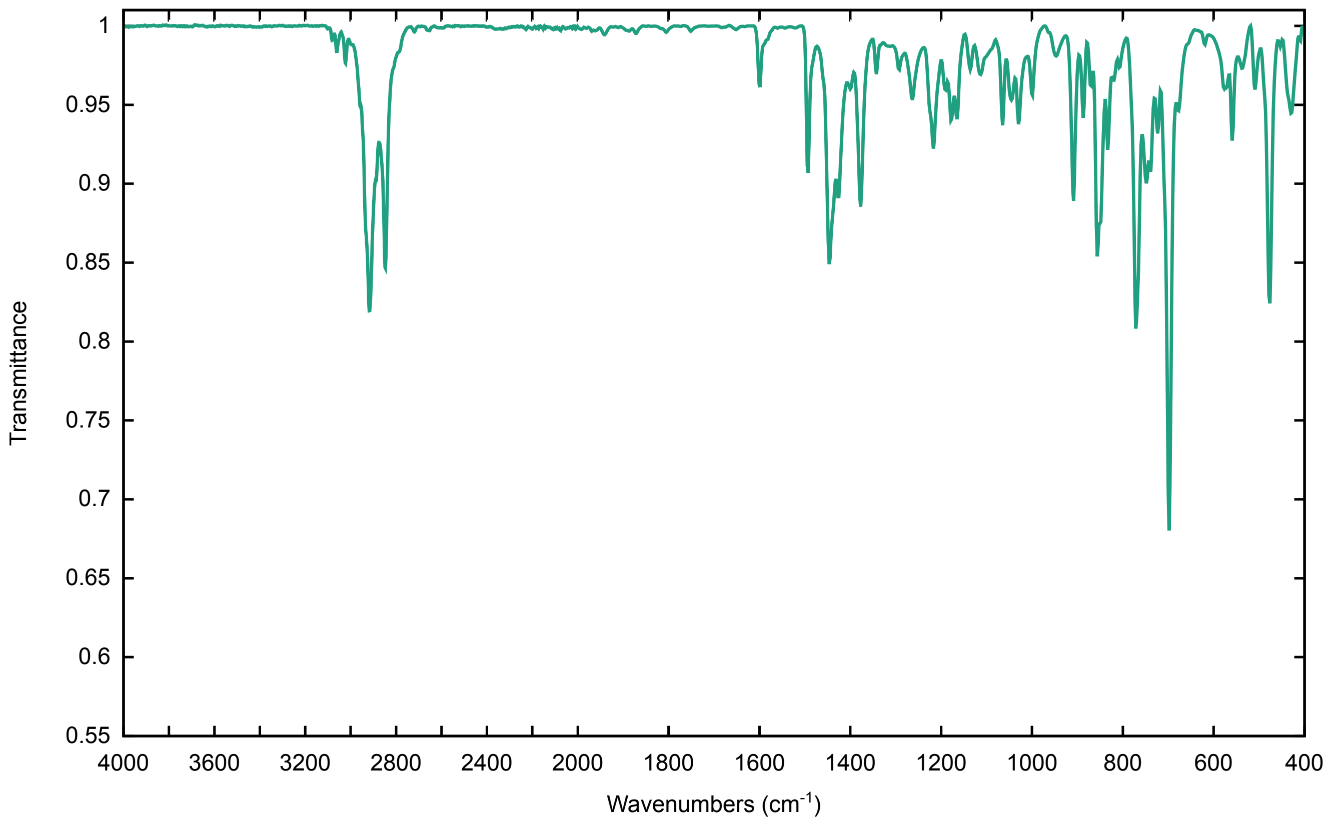

Figure S28 ATR-IR spectrum of Bn,Cy–DPC

10 Bn,Ph–DPC (9)

10.1 Synthesis of Bn,Ph–DPC (9)

Inside the glovebox, [Bn–P_2(dmb)$_2$]Br (495 mg, 1.25 mmol, 1 equiv) was slurried in 30 mL of THF in a 200 mL round bottom flask containing a stir bar. To this slurry, phenylmagnesium bromide (1.4 mL of 1 M solution in THF, 1.4 mmol, 1.1 equiv) was added dropwise by syringe. The reaction mixture became homogeneous after a few minutes. After stirring for 1 hour at room temperature, the homogeneous solution was placed under vacuum in order to remove THF. The residue was slurried in 60 mL Et_2O, then filtered through an alumina pad. The flask and alumina were rinsed with an additional 20 mL ether. The clear homogeneous filtrate was dried under vacuum, then 10mL ether were used to transfer the material into a vial. After drying under vacuum and triturating with pentane, 467 mg of white powder were obtained (1.18 mmol, 94% yield).

10.2 Characterization of Bn,Ph–DPC (9)

^{1}H NMR (400 MHz, C_6D_6, 25 °C) δ: 7.54 – 7.47 (m, 2H), 7.22 – 7.08 (m, 7H), 7.08 – 7.02 (m, 1H), 3.38 (dd, J = 13.2, 6.6 Hz, 2H), 2.88 (dd, J = 13.6, 6.3 Hz, 2H), 2.54 (d, J = 2.6 Hz, 2H), 2.12 (d, J = 13.1 Hz, 2H), 1.88 (m, 2H), 1.87 (d, J = 1.4 Hz, 6H), 1.75 (s, 6H) ppm.

^{13}C NMR (101 MHz, C_6D_6, 25 °C) δ: 141.71 (d, J = 16.4 Hz), 138.60 (d, J = 4.5 Hz), 132.18 (d, J = 18.7 Hz), 129.59 (d, J = 5.3 Hz), 128.74 (d, J = 6.2 Hz), 128.63 (d, J = 0.9 Hz), 128.53 (s), 126.05 (m), 125.24 (t, J = 6.0 Hz), 37.86 (dd, J = 16.1, 1.3 Hz), 36.81 (dd, J = 18.0, 1.5 Hz), 36.47 (d, J = 16.7 Hz), 19.98 (t, J = 12.5 Hz) ppm.

^{31}P{^{1}H} NMR (162 MHz, C_6D_6, 25 °C) δ: −45.53 (d, J = 5.9 Hz), −46.14 (d, J = 6.0 Hz) ppm.

ATR-IR: 2902, 2857, 1431, 1382, 854, 766, 746, 723, 699, 692, 480, 440 cm^{-1}.

Elemental analysis [%] found (calculated for $C_{25}H_{32}P_2$): C 75.69 (76.12), H 8.20 (8.18), N $<$ 0.02 (0).

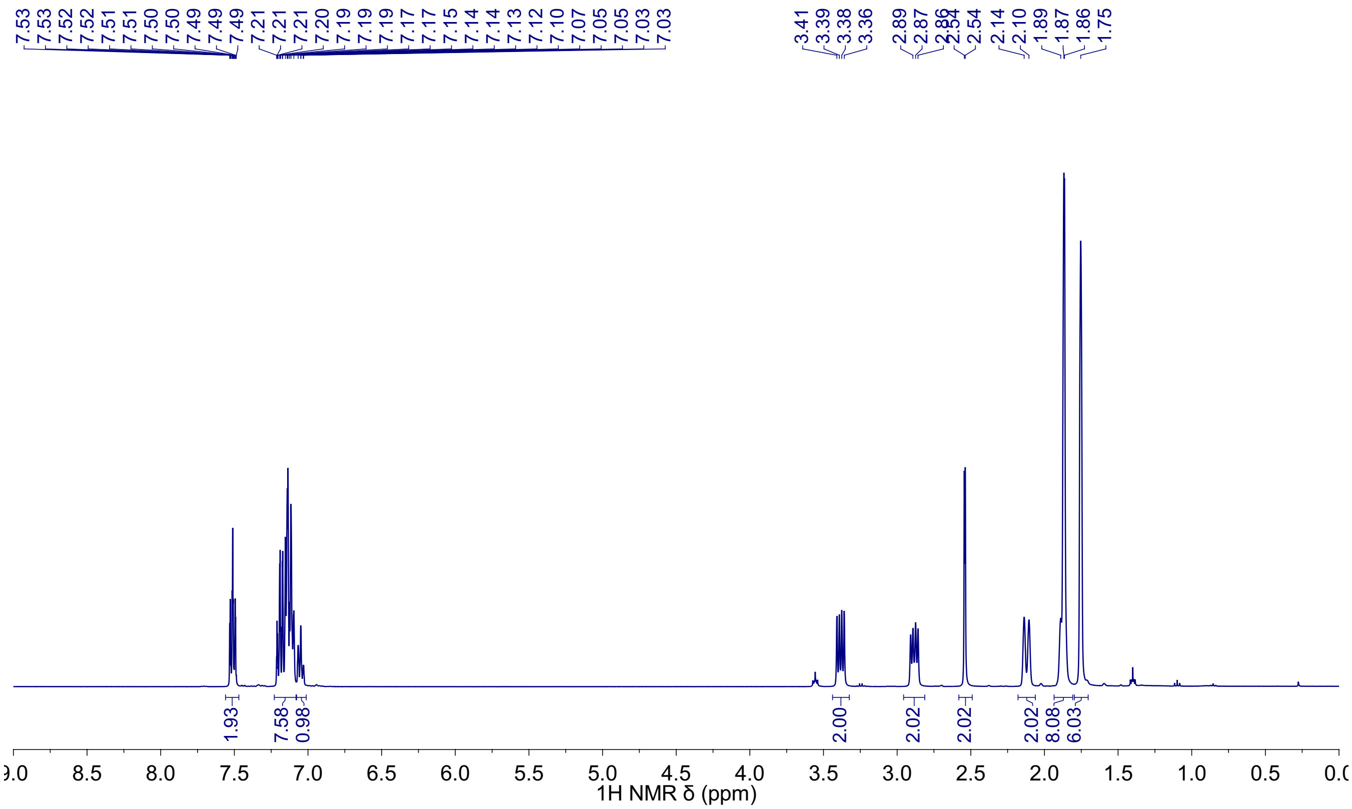

Figure S29 ^{1}H NMR spectrum of Bn,Ph–DPC in C_6D_6 at 25 °C

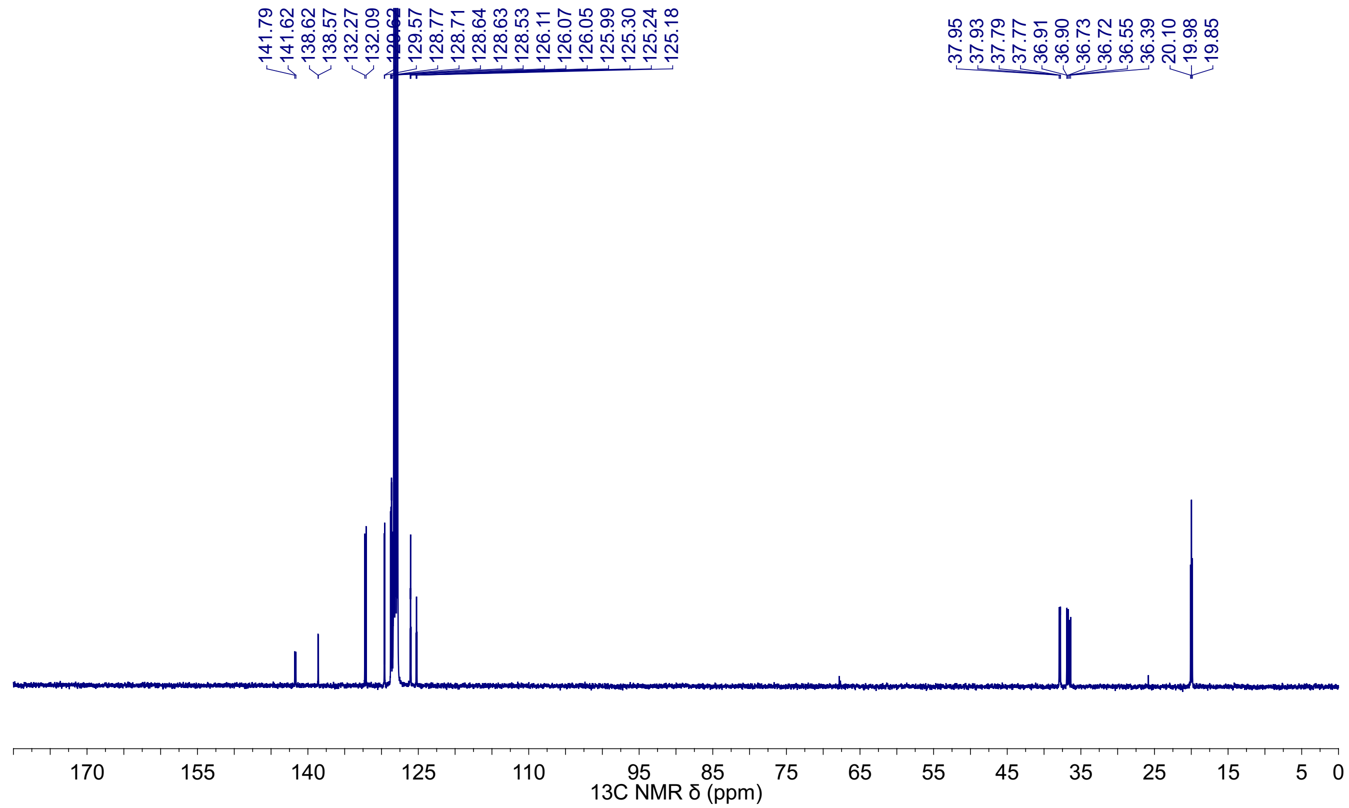

Figure S30 ^{13}C NMR spectrum of Bn,Ph–DPC in C_6D_6 at 25 °C

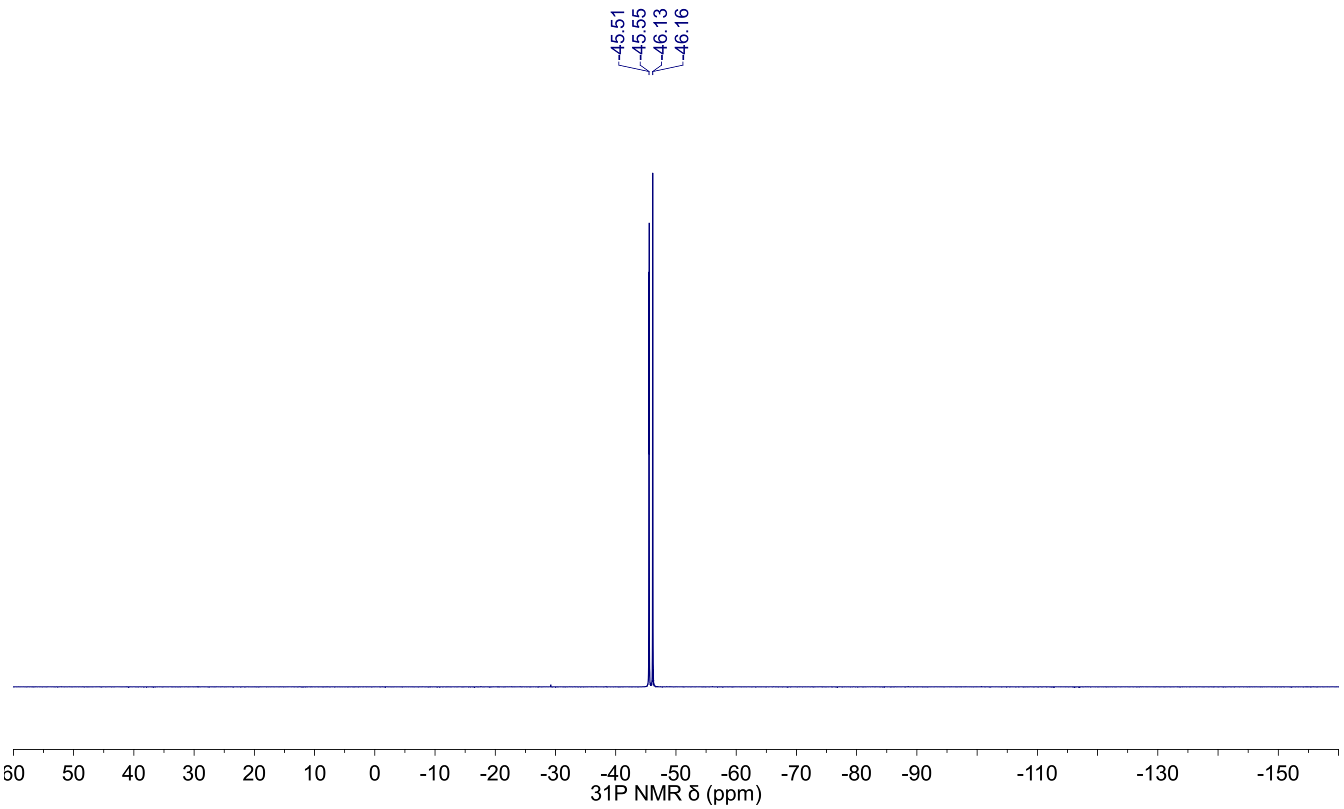

Figure S31 $^{31}P\{^1H\}$ NMR spectrum of Bn,Ph–DPC in C_6D_6 at 25 °C

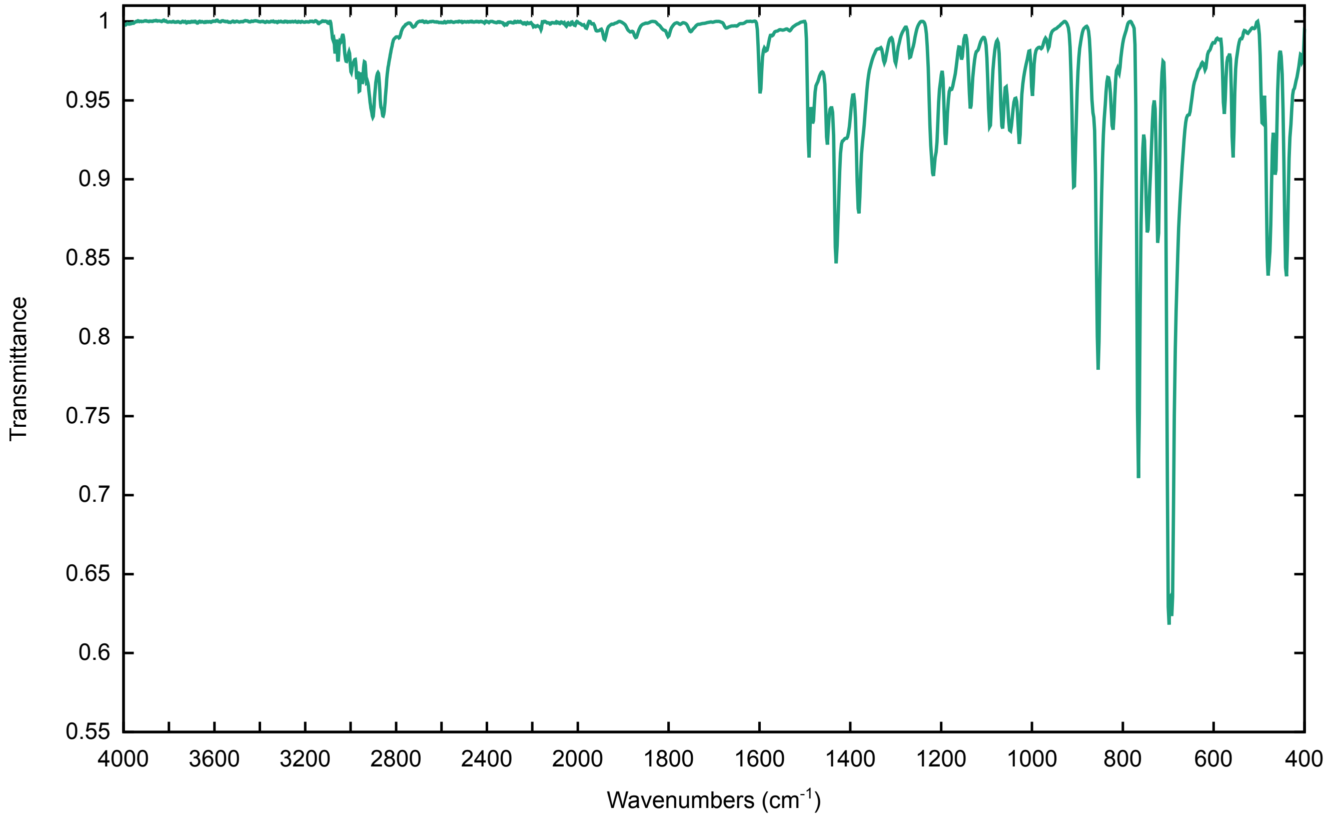

Figure S32 ATR-IR spectrum of Bn,Ph–DPC

11 Bn,Mes–DPC (10)

11.1 Synthesis of Bn,Mes–DPC (10)

Inside the glovebox, [Bn–P_2(dmb)$_2$]Br (483 mg, 1.21 mmol, 1 equiv) was slurried in 30 mL of THF in a 200 mL round bottom flask containing a stir bar. To this slurry, 2-mesitylmagnesium bromide (1.4 mL of 1 M solution in THF, 1.4 mmol, 1.15 equiv) was added dropwise by syringe. After stirring for 2 hours at room temperature, the now homogeneous solution was placed under vacuum in order to remove THF. The residue was slurried in 70 mL Et_2O, then filtered through an alumina pad. The flask and alumina were rinsed with an additional 20 mL ether. The clear homogeneous filtrate was dried under vacuum, then 10mL ether were used to transfer the material into a vial. After drying under vacuum and analyzing by $^{31}P\{^1H\}$ NMR, several impurities were apparent. The synthesis was repeated, and the two product crops combined. This crude solid was washed with 2 x 5mL pentane in an attempt to remove some of the impurities seen in the baseline of the $^{31}P\{^1H\}$ NMR. After drying the remaining powder, 486 mg of white powder were obtained (1.11 mmol, 46% yield). The compound was still not spectroscopically pure.

11.2 Characterization of Bn,Mes–DPC (10)

^{1}H NMR (400 MHz, C_6D_6, 25 °C) δ: 7.16 (m, 2H), 7.07 (m, 3H), 6.80 (s, 2H), 3.94 (dd, J = 13.5, 4.7 Hz, 2H), 2.97 (dd, J = 13.3, 6.2 Hz, 2H), 2.65 (s, 6H), 2.60 (d, J = 3.4 Hz, 2H), 2.12 (s, 3H), 1.93 (d, J = 12.6 Hz, 4H), 1.86 (s, 6H), 1.78 (d, J = 3.5 Hz, 6H) ppm.

^{13}C NMR (101 MHz, C_6D_6, 25 °C) δ: 144.24 (d, J = 15.9 Hz), 138.76 (d, J = 1.0 Hz), 138.36 (d, J = 3.6 Hz), 133.53 (d, J = 25.2 Hz), 130.18 (d, J = 3.7 Hz), 129.63 (d, J = 4.9 Hz), 128.56 (s), 127.07 (dd, J = 11.0, 6.1 Hz), 126.06 (d, J = 2.0 Hz), 125.94 – 125.75 (m), 37.33 (dd, J = 18.4, 2.1 Hz), 36.68 (d, J = 16.9 Hz), 34.58 (dd, J = 17.3, 1.4 Hz), 24.17 (d, J = 19.9 Hz), 20.95 (s), 20.17 (d, J = 12.4 Hz), 19.90 (d, J = 14.6 Hz) ppm.

$^{31}P\{^1H\}$ NMR (162 MHz, C_6D_6, 25 °C) δ: −45.57 (d, J = 9.4 Hz), −47.50 (d, J = 9.4 Hz) ppm.

ATR-IR: 2975, 2877, 1491, 1452, 1429, 1377, 1213, 1027, 856, 844, 771, 704, 613, 478 cm^{-1}.

Elemental analysis [%] found (calculated for $C_{28}H_{38}P_2$): C 76.19 (77.04), H 8.95 (8.77), N $<$ 0.02 (0).

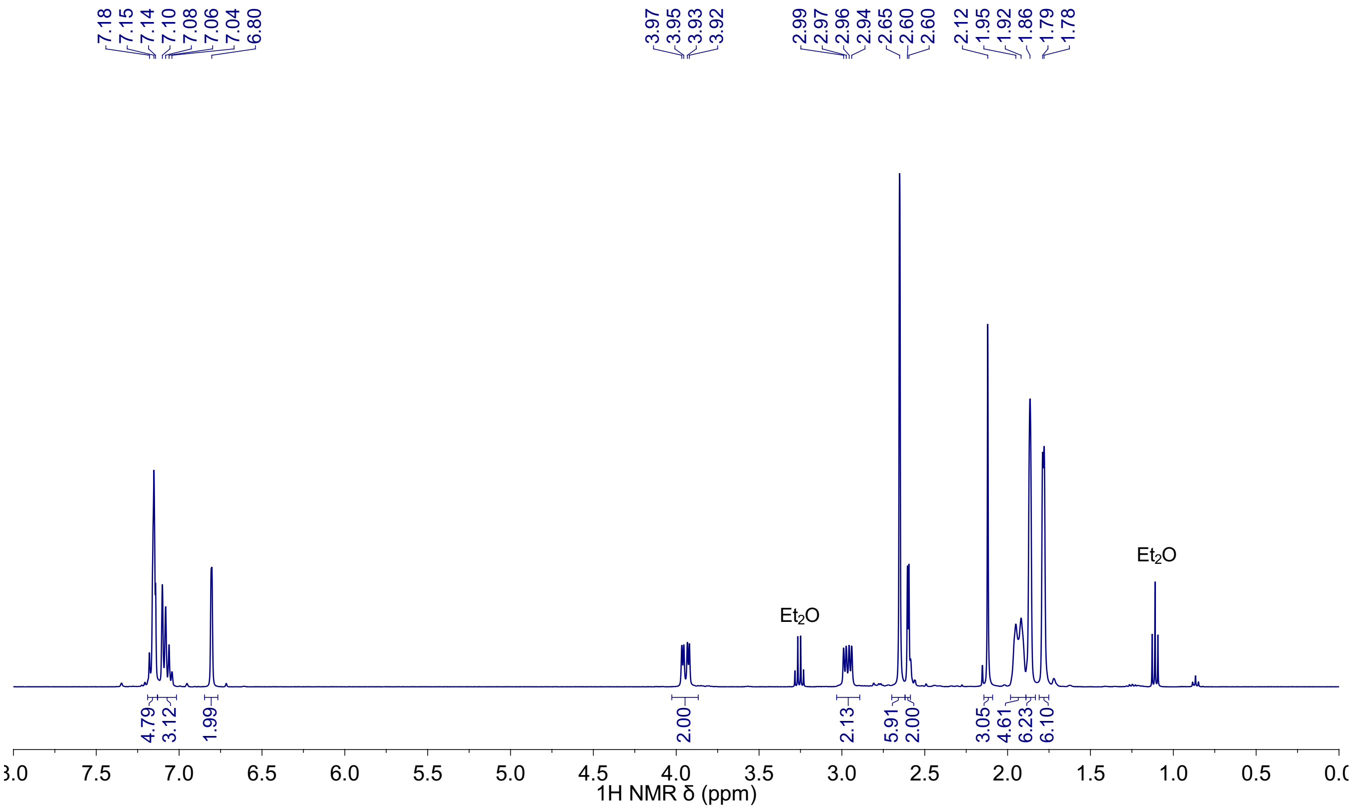

Figure S33 ^{1}H NMR spectrum of Bn,Mes–DPC in C_6D_6 at 25 °C

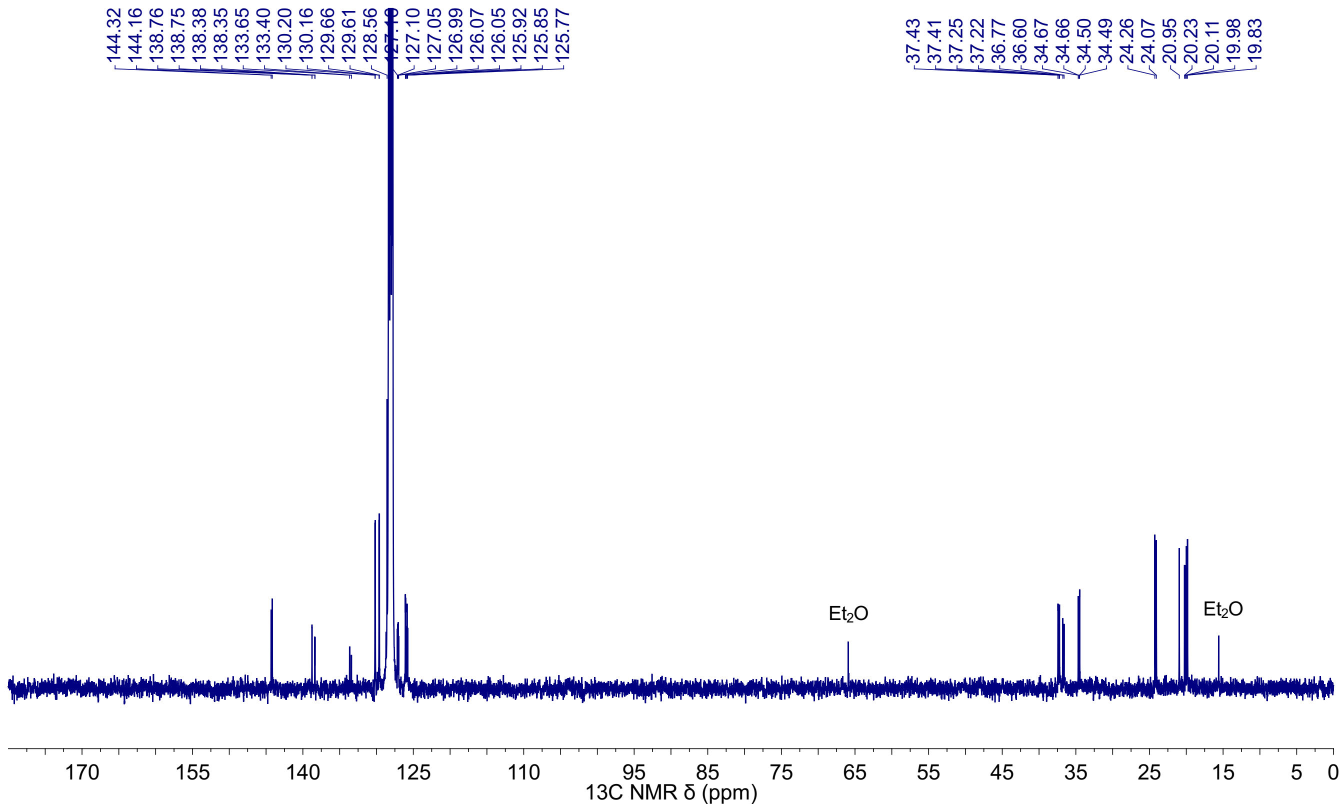

Figure S34 ^{13}C NMR spectrum of Bn,Mes–DPC in C_6D_6 at 25 °C

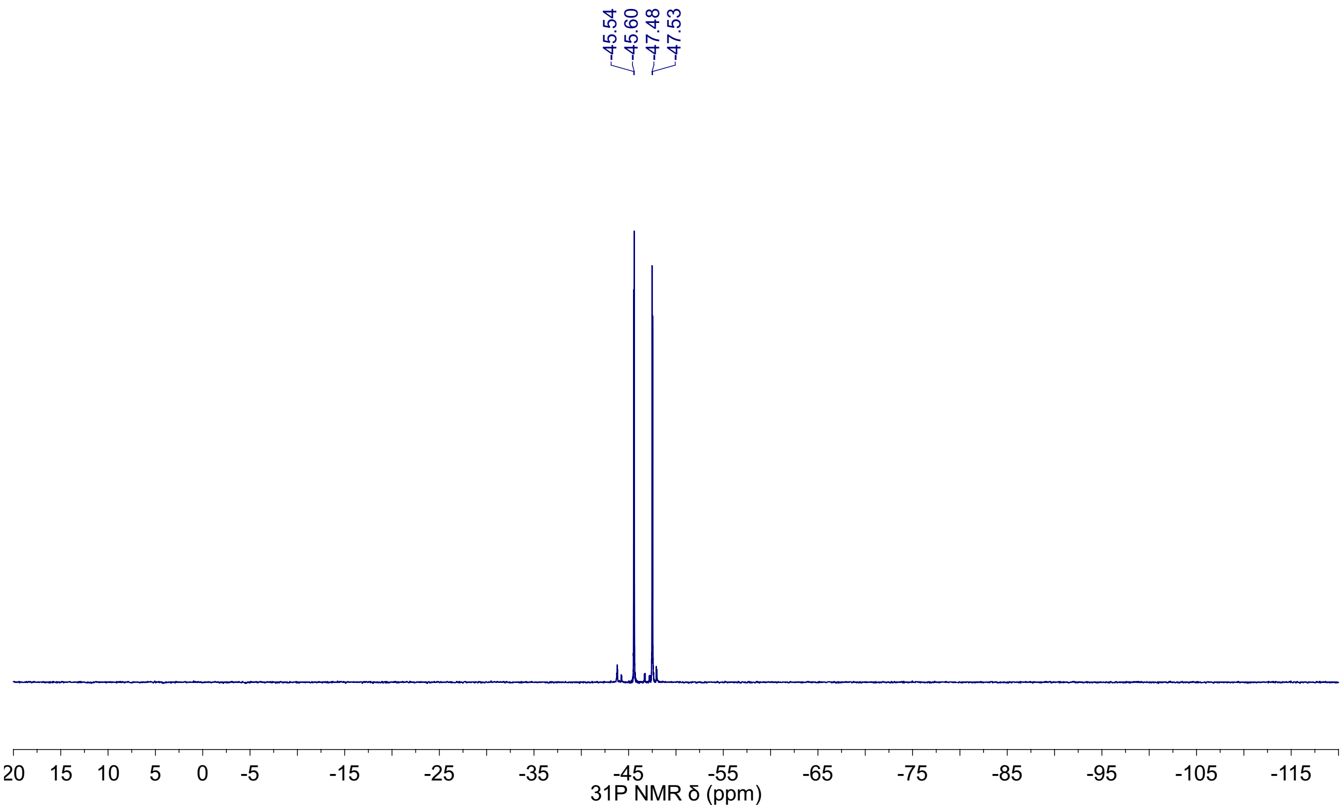

Figure S35 $^{31}P\{^1H\}$ NMR spectrum of Bn,Mes–DPC in C_6D_6 at 25 °C

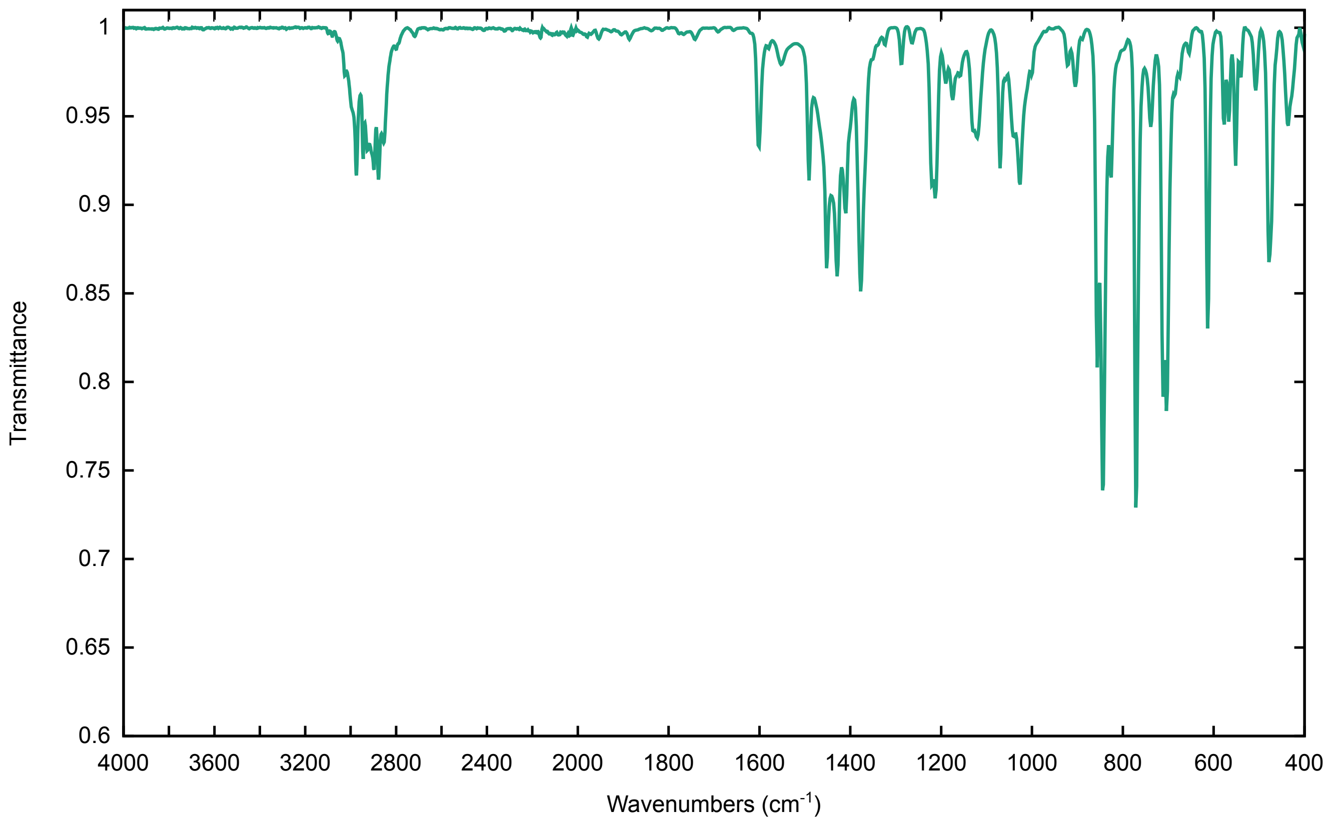

Figure S36 ATR-IR spectrum of Bn,Mes–DPC

12 $Cl_2P_2(dmb)_2$ (11)

12.1 Synthesis of $Cl_2P_2(dmb)_2$ (11)

Inside the glovebox, P_2dmb_2 (950 mg, 4.2 mmol, 1.05 equiv) was dissolved in 30 mL of dichloromethane in a 200 mL round bottom flask containing a stir bar. To this homogeneous solution, a solution of hexachloroethane (945 mg, 4.0 mmol, 1 equiv) in 20 mL dichloromethane was added dropwise at room temperature. The homogeneous reaction mixture was allowed to stir at room temperature for 15 min, after which it was placed under vacuum to remove volatiles. The sticky residue was triturated with 2 x 10 mL Et_2O. The solid was then slurried in 15 mL Et_2O, filtered, then washed with 2 x 8 mL Et_2O. After drying the solids, 1.25 g of pale yellow powder were obtained (4.2 mmol, quantitative yield - extra mass presumably due to a small amount of by-product(s) - see NMR data).

12.2 Characterization of $Cl_2P_2(dmb)_2$ (11)

^{1}H NMR (400 MHz, $CDCl_3$, 25 °C) δ: 4.87 (br s, 4H), 2.95 (br s, 4H), 1.84 (br s, 12H) ppm.

^{13}C NMR (101 MHz, $CDCl_3$, 25 °C) δ: 128.03 (br s), 32.56 (br s), 21.10 (s) ppm.

^{31}P{^{1}H} NMR (162 MHz, $CDCl_3$, 25 °C) δ: 144.05 (br s), −47.12 (br s) ppm.

^{1}H NMR (400 MHz, $CDCl_3$, −40 °C) δ: 5.30 (d, could not accurately determine J due to overlap with CH_2Cl_2, 2H), 4.13 (dd, J = 26.2, 12.6 Hz, 2H), 3.20 (dd, J = 18.1, 16.6 Hz, 2H), 2.53 (ddd, J = 40.7, 22.6, 13.4 Hz, 2H), 1.81 (d with unresolved coupling, 6H), 1.79 (s, 6H) ppm.

^{31}P{^{1}H} NMR (162 MHz, $CDCl_3$, −40 °C) δ: 143.40 (d, J = 291 Hz), −48.72 (d, J = 291 Hz) ppm.

^{31}P{^{1}H} NMR (202 MHz, $CDCl_3$, 0 °C) δ: 145.01 (d, J = 295 Hz), −47.06 (d, J = 295 Hz) ppm.

^{1}H NMR of $Cl_2P_2(dmb)_2$ + $GaCl_3$ (400 MHz, $CDCl_3$, 25 °C) δ: 3.65 – 3.43 (m, 4H), 3.06 – 2.66 (m, 4H), 1.97 (s, 6H), 1.92 (d, J = 8.1 Hz, 6H) ppm.

^{31}P{^{1}H} NMR of $Cl_2P_2(dmb)_2$ + $GaCl_3$ (162 MHz, $CDCl_3$, 25 °C) δ: 120.40 (d, J = 322.4 Hz), −48.93 (d, J = 322.4 Hz) ppm.

ATR-IR: 2928, 2847, 1441, 1382, 1157, 1121, 1055, 905, 857, 835, 772, 718, 573, 543, 424 cm^{-1}.

Elemental analysis [%] found (calculated for $C_{12}H_{20}Cl_2P_2$): C 46.15 (48.51), H 6.30 (6.78), N $<$ 0.02 (0).

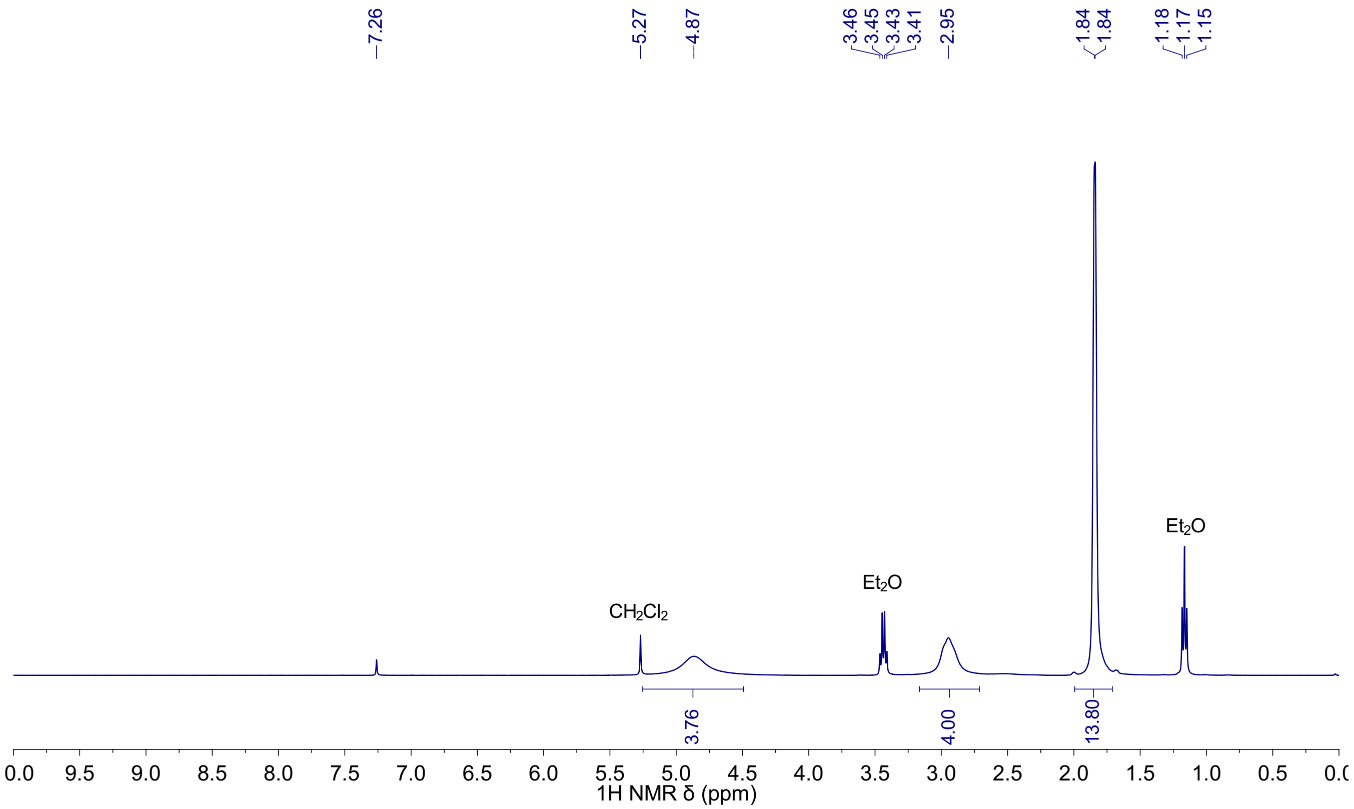

Figure S37 ^{1}H NMR spectrum of $Cl_2P_2(dmb)_2$ in $CDCl_3$ at 25 °C

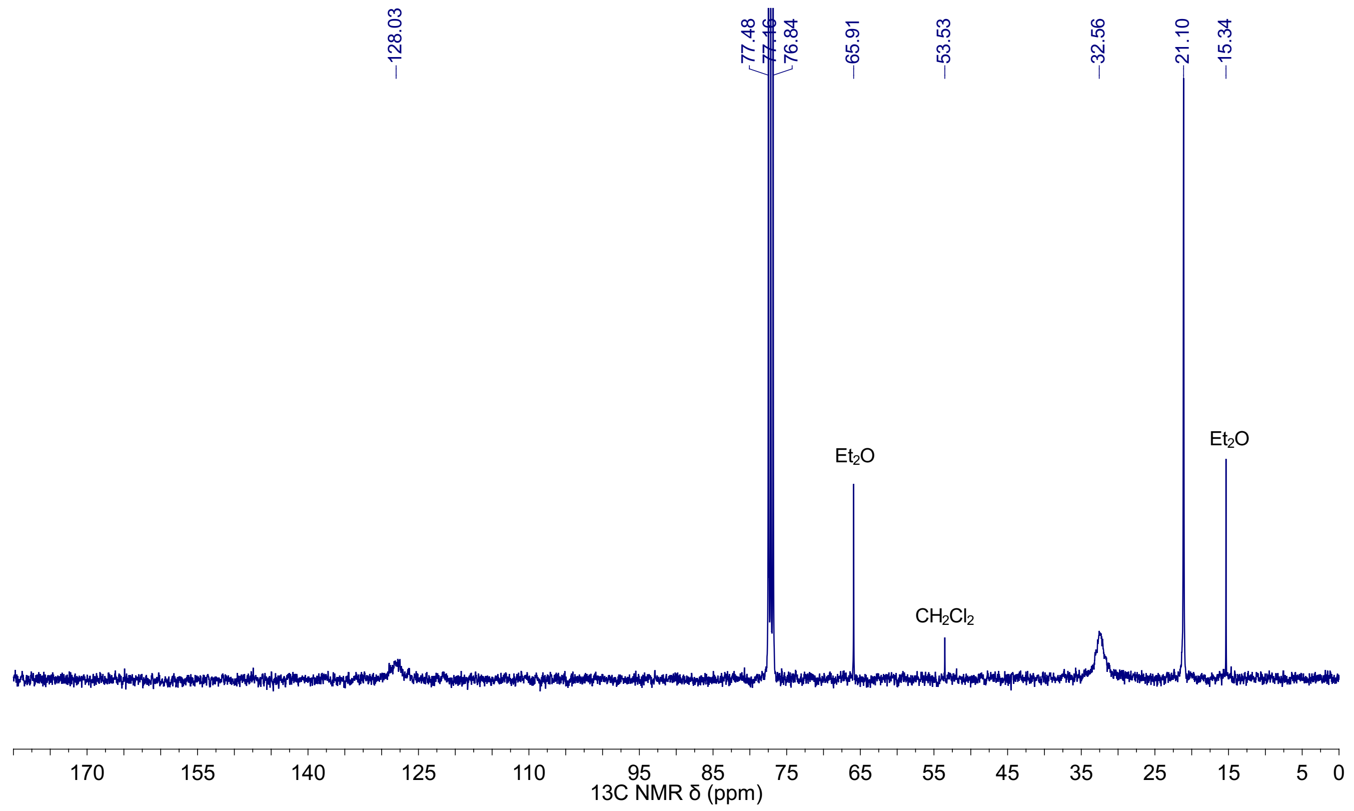

Figure S38 ^{13}C NMR spectrum of $Cl_2P_2(dmb)_2$ in $CDCl_3$ at 25 °C

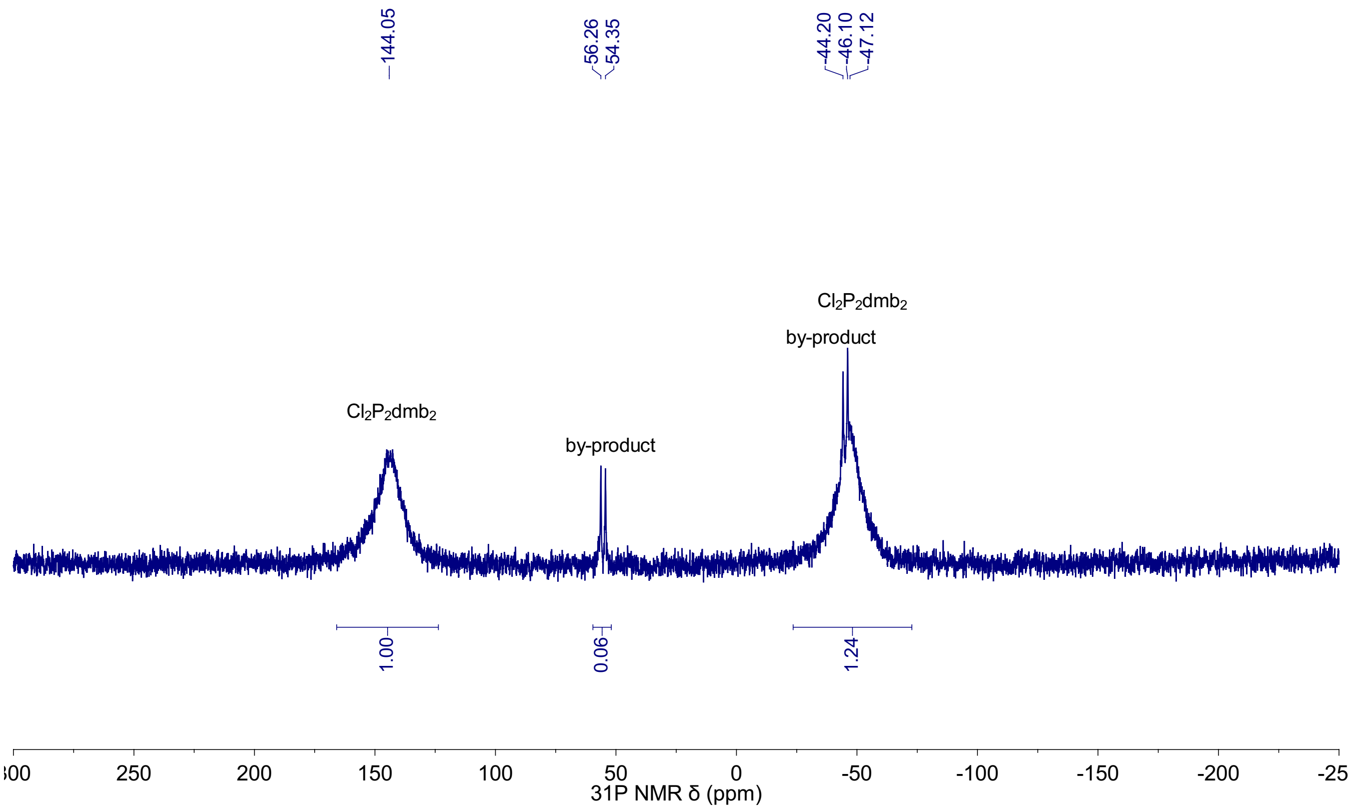

Figure S39 $^{31}P\{^1H\}$ NMR spectrum of $Cl_2P_2(dmb)_2$ in $CDCl_3$ at 25 °C

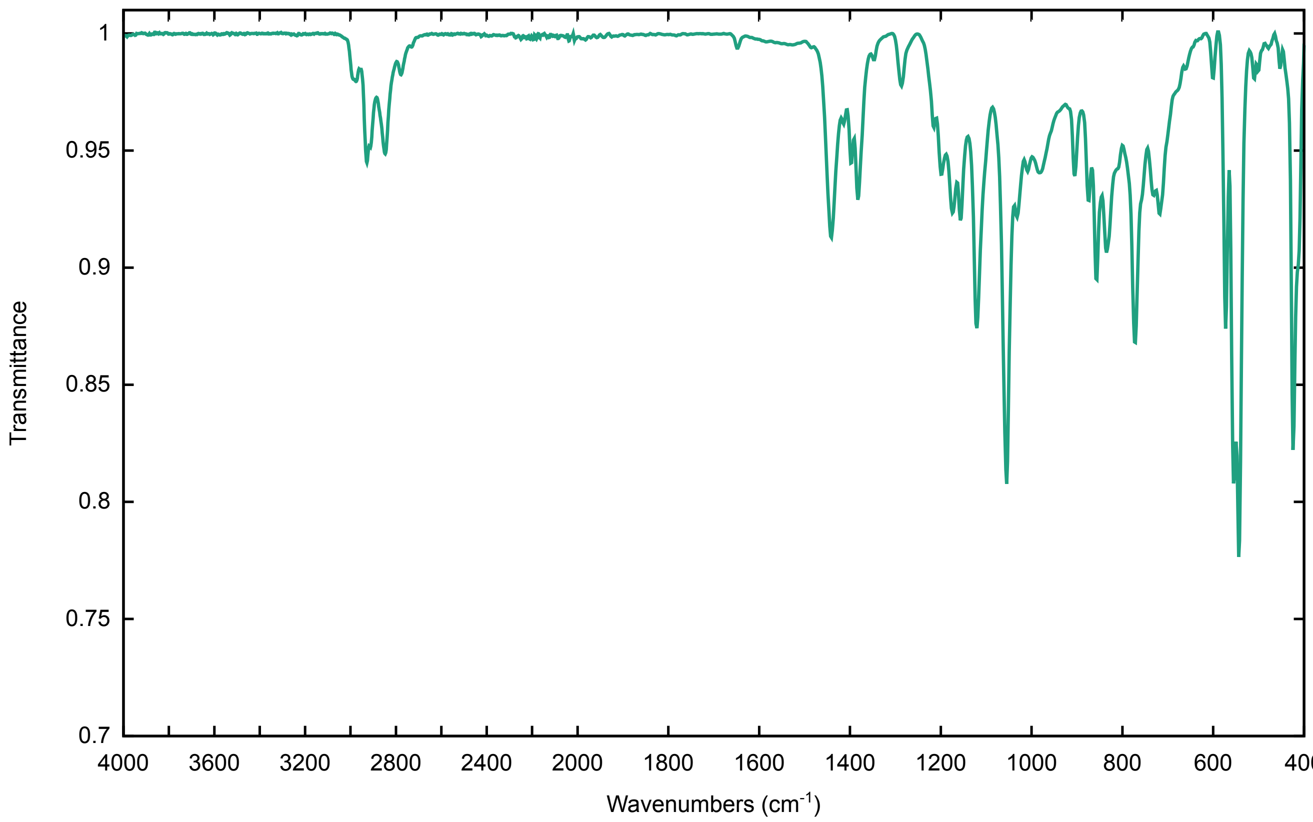

Figure S40 ATR-IR spectrum of $Cl_2P_2(dmb)_2$

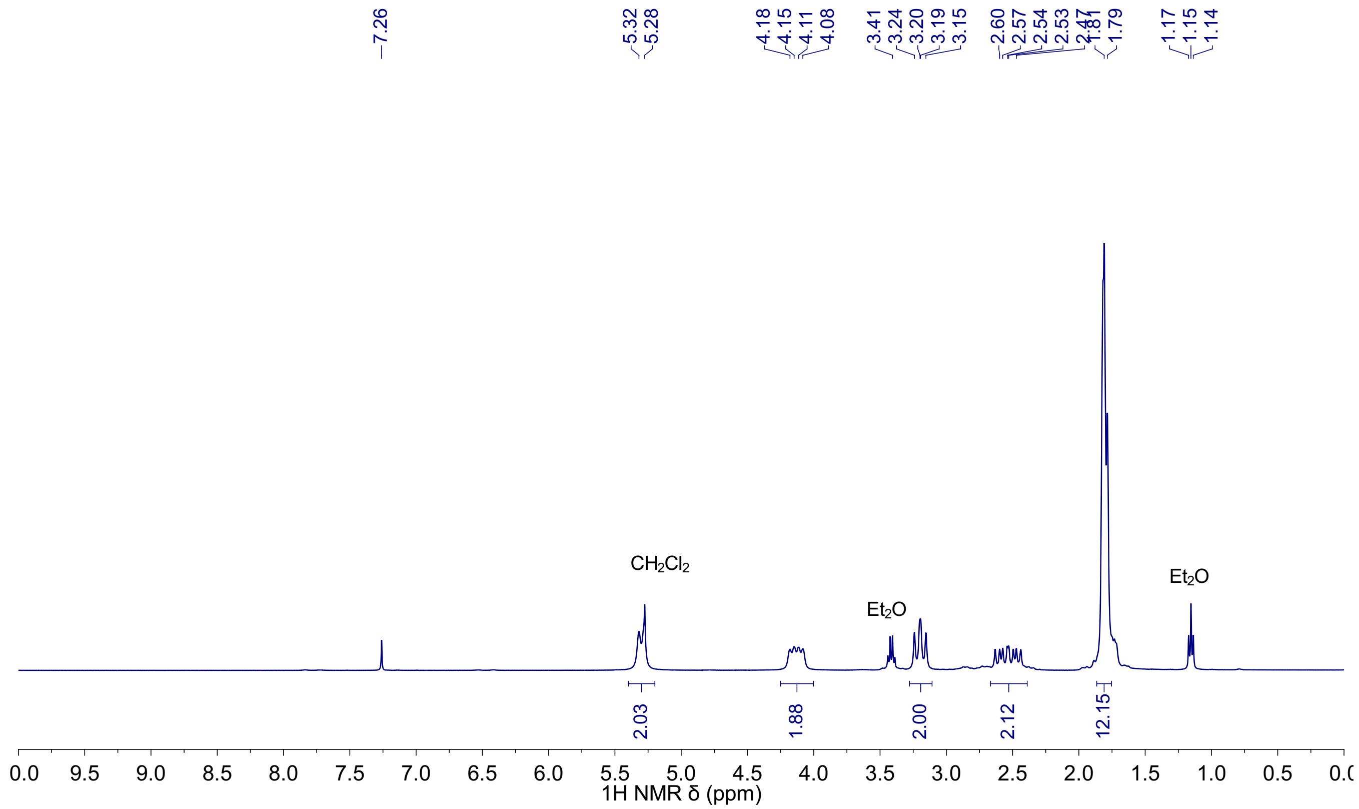

Figure S41 ^{1}H NMR spectrum of $Cl_2P_2(dmb)_2$ in $CDCl_3$ at −40 °C

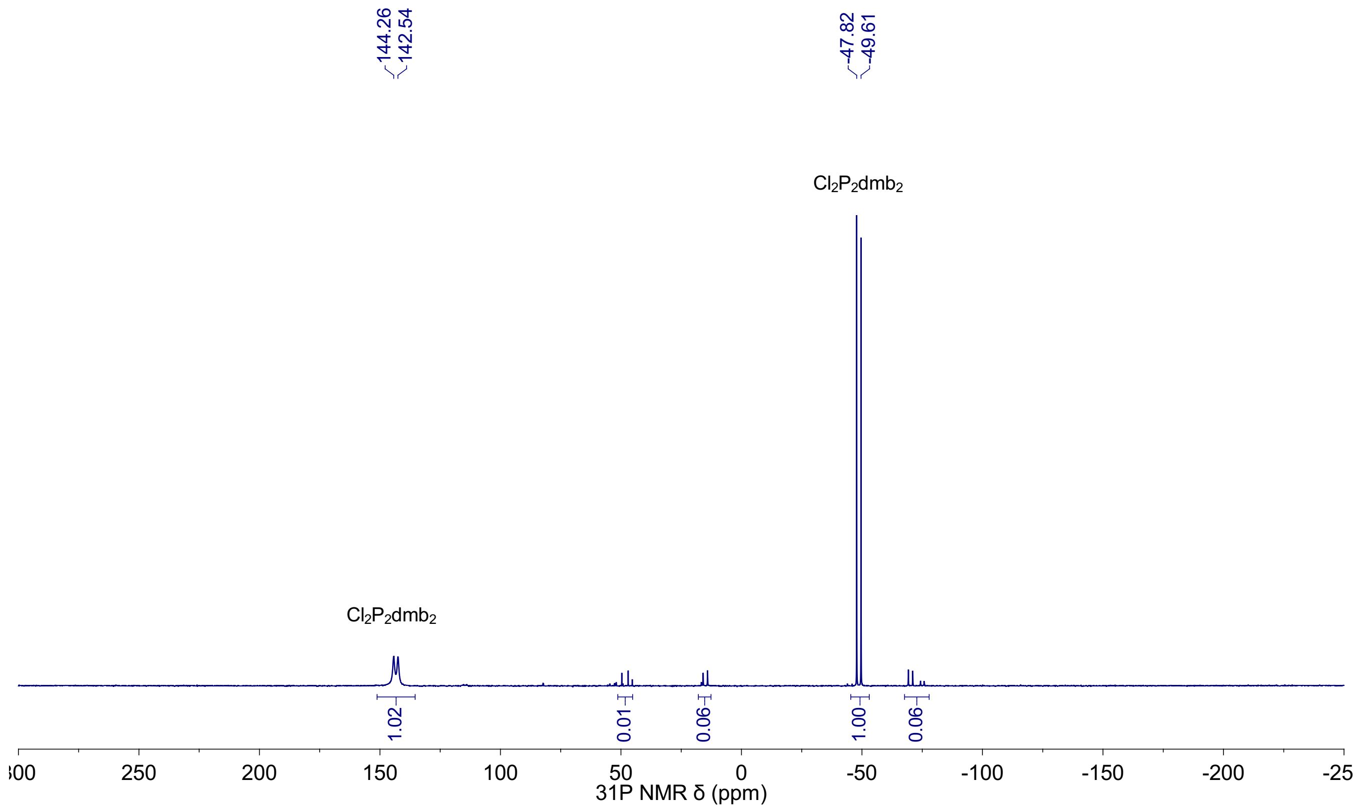

Figure S42 $^{31}P\{^{1}H\}$ NMR spectrum of $Cl_2P_2(dmb)_2$ in $CDCl_3$ at −40 °C

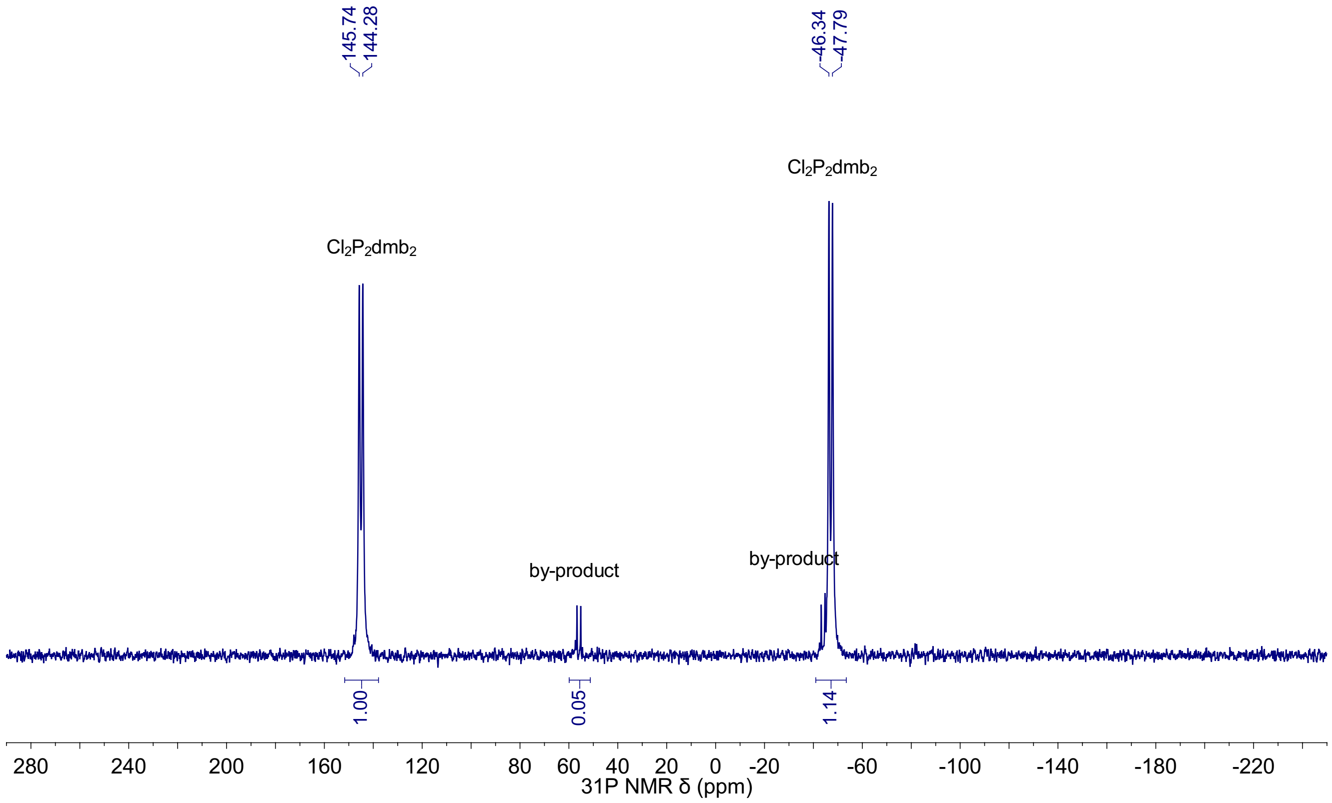

Figure S43 $^{31}P\{^1H\}$ NMR spectrum of $Cl_2P_2(dmb)_2$ in $CDCl_3$ at 0 °C

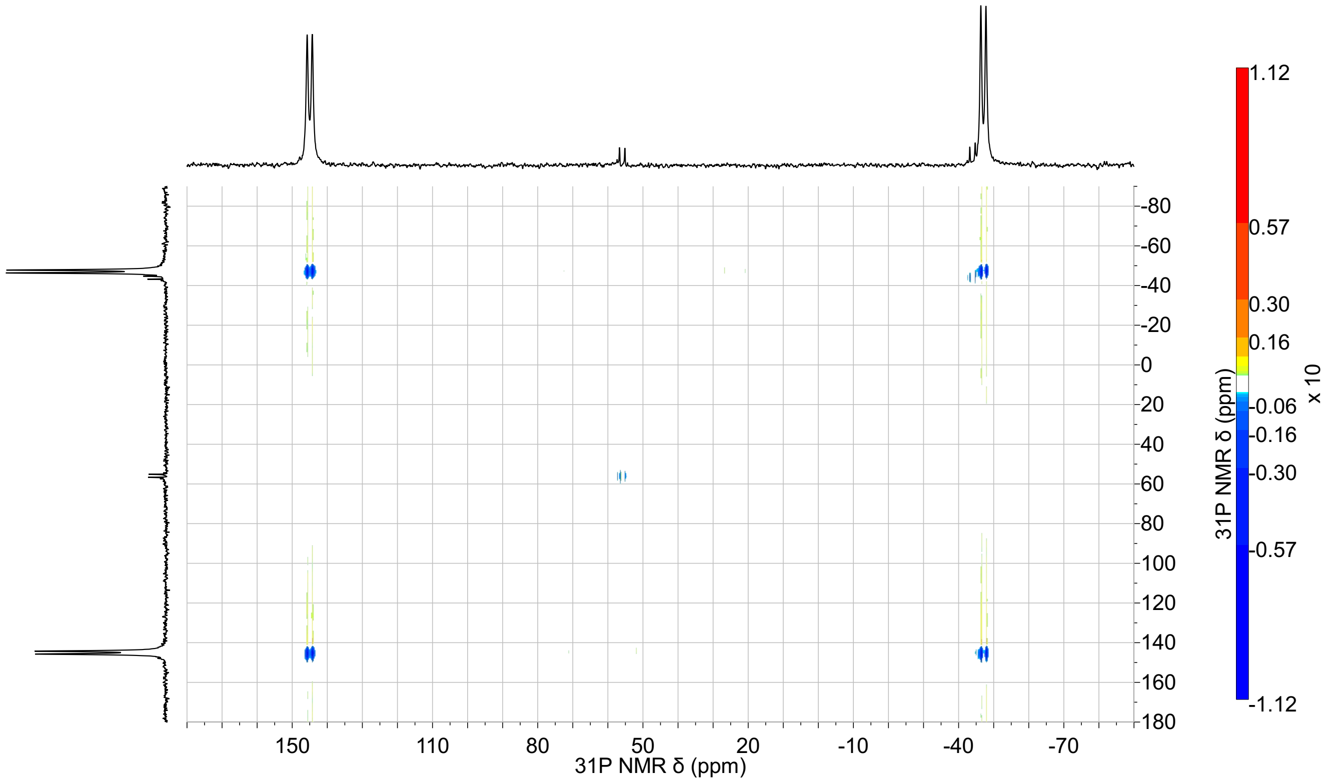

Figure S44 $^{31}P\{^1H\}$ 2D EXSY spectrum of $Cl_2P_2(dmb)_2$ in $CDCl_3$ at 0 °C

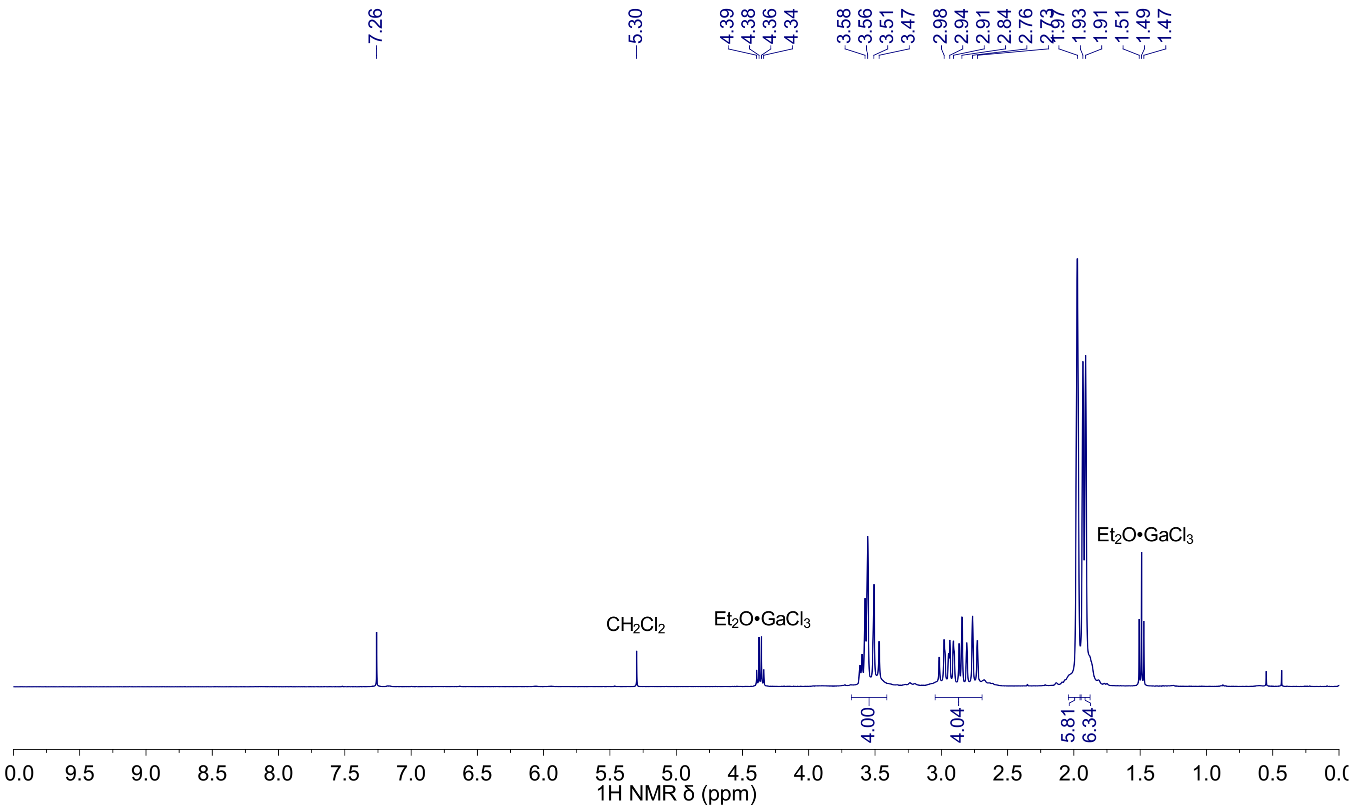

Figure S45 ^{1}H NMR spectrum of $Cl_2P_2(dmb)_2$ (1 equiv) + $GaCl_3$ (1.2 equiv) in $CDCl_3$ at 25 °C

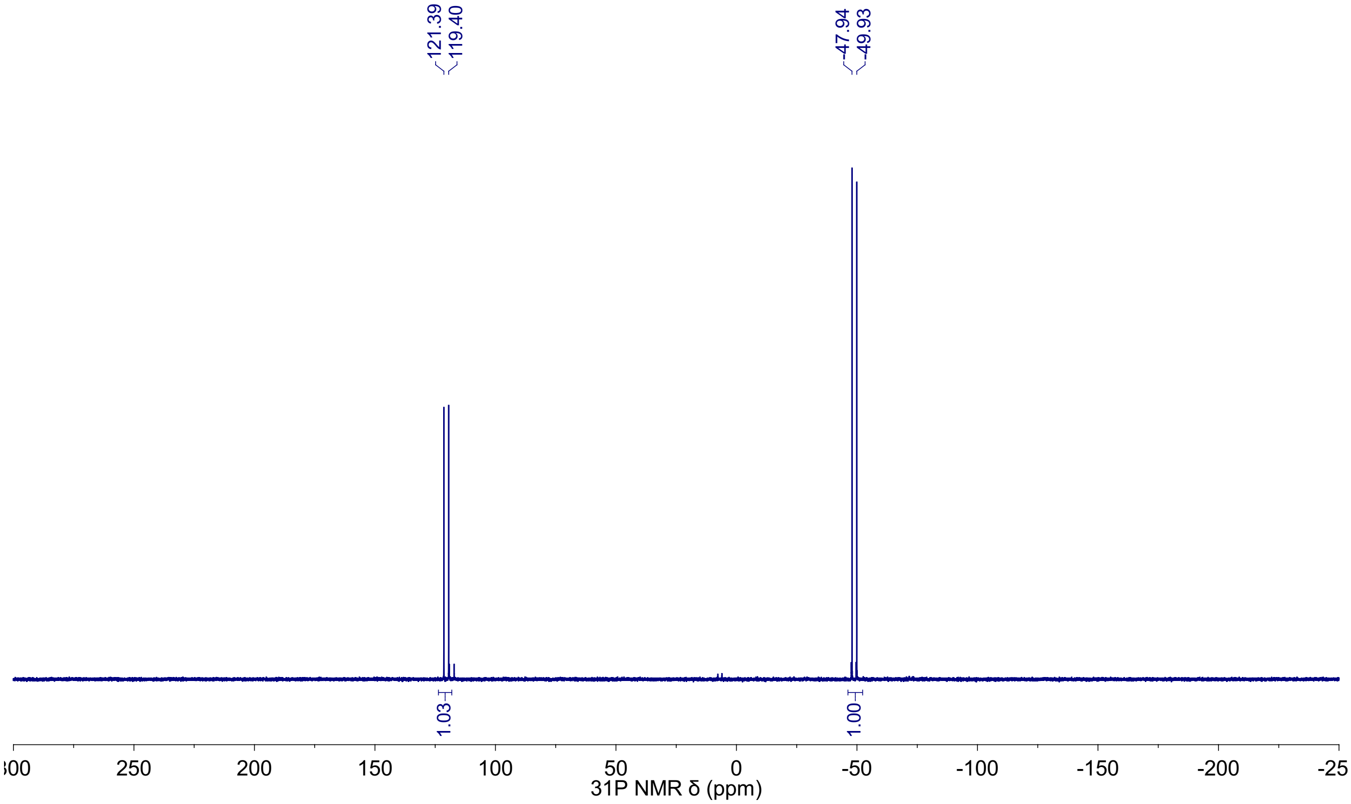

Figure S46 ^{31}P{^{1}H} NMR spectrum of $Cl_2P_2(dmb)_2$ (1 equiv) + $GaCl_3$ (1.2 equiv) in $CDCl_3$ at 25 °C

13 Cy_2–DPC (12)

13.1 Synthesis of Cy_2–DPC (12)

Inside the glovebox, $Cl_2P_2(dmb)_2$ (1.04 g, 3.5 mmol, 1 equiv) was slurried in 60 mL of THF in a 200 mL round bottom flask containing a stir bar. To this slurry, cyclohexylmagnesium chloride (7.5 mL of 1 M solution in 2-Me-THF, 7.5 mmol, 2.14 equiv) was added dropwise by syringe. After the addition was complete, most solids has dissolved and the solution had turned yellow-orange. The mixture was stirred at room temperature for 2 hours, time after which the mixture had become homogeneous and darker in color (brown). The mixture was then placed under vacuum in order to remove THF. The residue was slurried in 100 mL Et_2O, then filtered through an alumina pad. The flask and alumina were rinsed with an additional 40 mL ether. The yellow homogeneous filtrate was dried under vacuum, then ca. 10mL ether were used to transfer the material into a vial. After drying under vacuum, a sticky solid was obtained. The colored impurities were visibly oily (the sample did not look homogeneous). Hexanes (2mL) were added to the sample in order to extract the colored impurities. The slurry was stirred vigorously for 5 min, then the vial was then placed in the freezer for 1 hour. The sample was then filtered, and the solids washed with ca. 1mL hexanes. Care must be taken to use only a minimal ammount of solvent for washing as Cy_2–DPC is extremely soluble. After drying under vacuum, 646 mg of off-white powder were obtained (1.65 mmol, 47% yield).

Alternative synthesis: Inside the glovebox, $Cl_2P_2(dmb)_2$ (1.01 g, 3.41 mmol, 1 equiv) was slurried in 60 mL ether in a 200 mL round bottom flask containing a stir bar. To this slurry, cyclohexylmagnesium chloride (7.0 mL of 1 M solution in 2-Me-THF, 7.0 mmol, 2.05 equiv) was added dropwise by syringe. After the addition was complete, the reaction mixture remained heterogeneous. The mixture was stirred at room temperature for 4 hours, time after which the mixture was filtered through an alumina pad. The flask and alumina were rinsed with an additional 4 x 20 mL Et_2O. The homogeneous filtrate was dried under vacuum, then ca. 10mL ether were used to transfer the material into a vial. After drying under vacuum, the solid was triturated with pentane and further dried under vacuum to afford 992 mg of off-white powder (2.53 mmol, 74% yield).

13.2 Characterization of Cy_2–DPC (12)

^{1}H NMR (400 MHz, C_6D_6, 25 °C) δ: 3.06 (dd, J = 12.9, 5.9 Hz, 4H), 1.97 (d, J = 13.1 Hz, 4H), 1.92 (s, 12H), 1.87 – 1.59 (m, 10H), 1.33 – 1.08 (m, 12H) ppm.

^{13}C NMR (101 MHz, C_6D_6, 25 °C) δ: 125.56 (t, J = 5.6 Hz), 37.45 (d, J = 12.8 Hz), 34.26 (d, J = 20.4 Hz), 30.15 – 29.37 (m), 28.06 – 27.20 (m), 26.94 (s), 20.45 – 19.93 (m) ppm.

^{31}P{^{1}H} NMR (162 MHz, C_6D_6, 25 °C) δ: −38.68 ppm.

ATR-IR: 2921, 2849, 1447, 1376, 904, 883, 848 cm^{-1}.

Elemental analysis [%] found (calculated for $C_{24}H_{42}P_2$)(calculated for a mixture of 98% Cy_2–DPC and 2% P_2dmb_2): C 73.70 (73.43)(73.32), H 10.62 (10.78)(10.76), N $<$ 0.02 (0)(0).

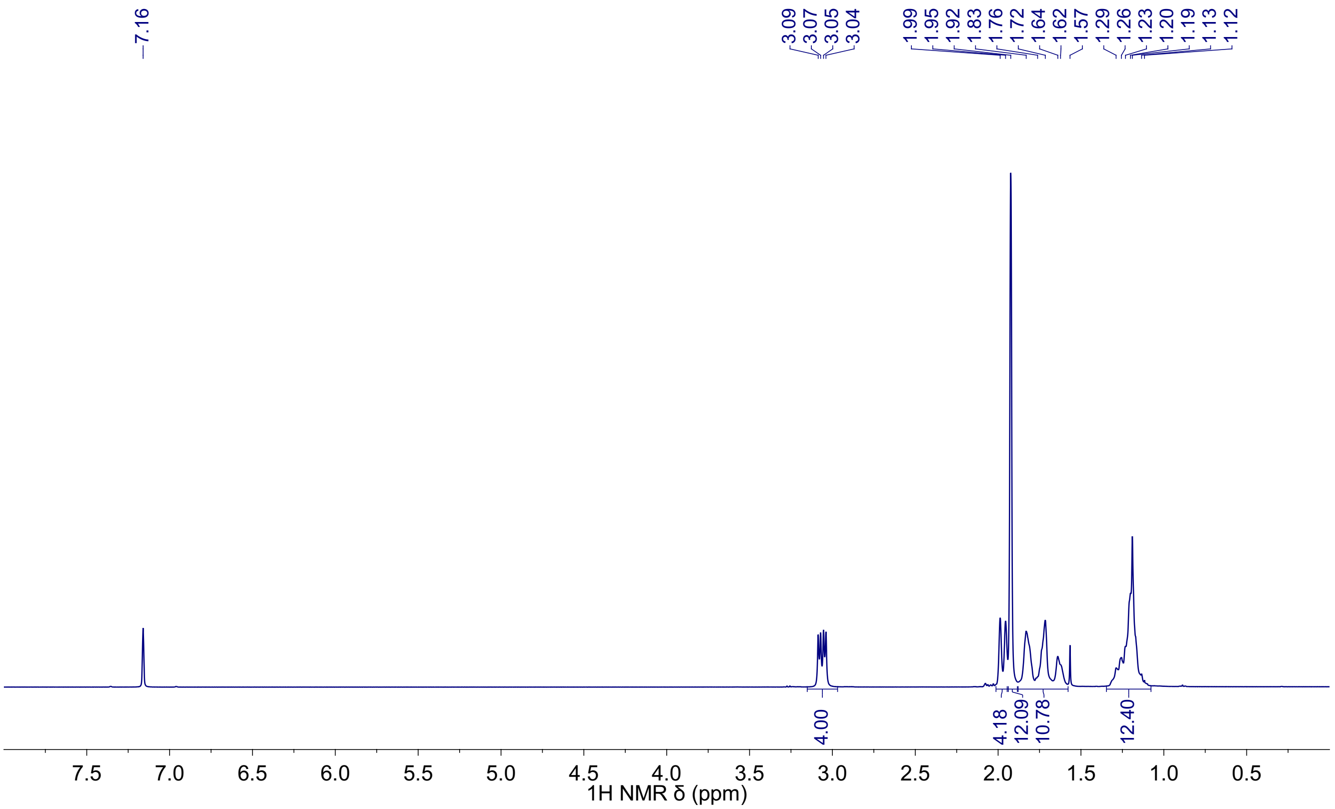

Figure S47 ^{1}H NMR spectrum of Cy_2–DPC in C_6D_6 at 25 °C

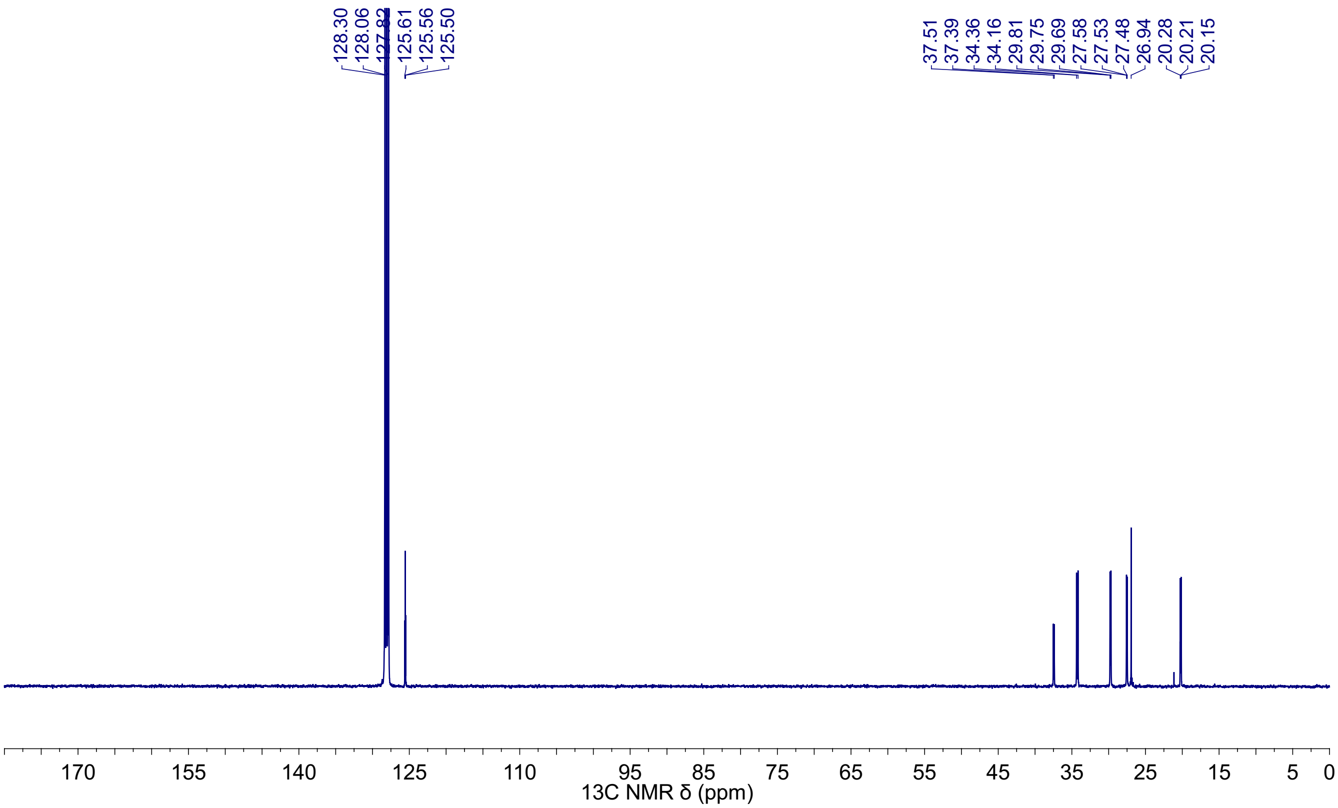

Figure S48 ^{13}C NMR spectrum of Cy_2–DPC in C_6D_6 at 25 °C

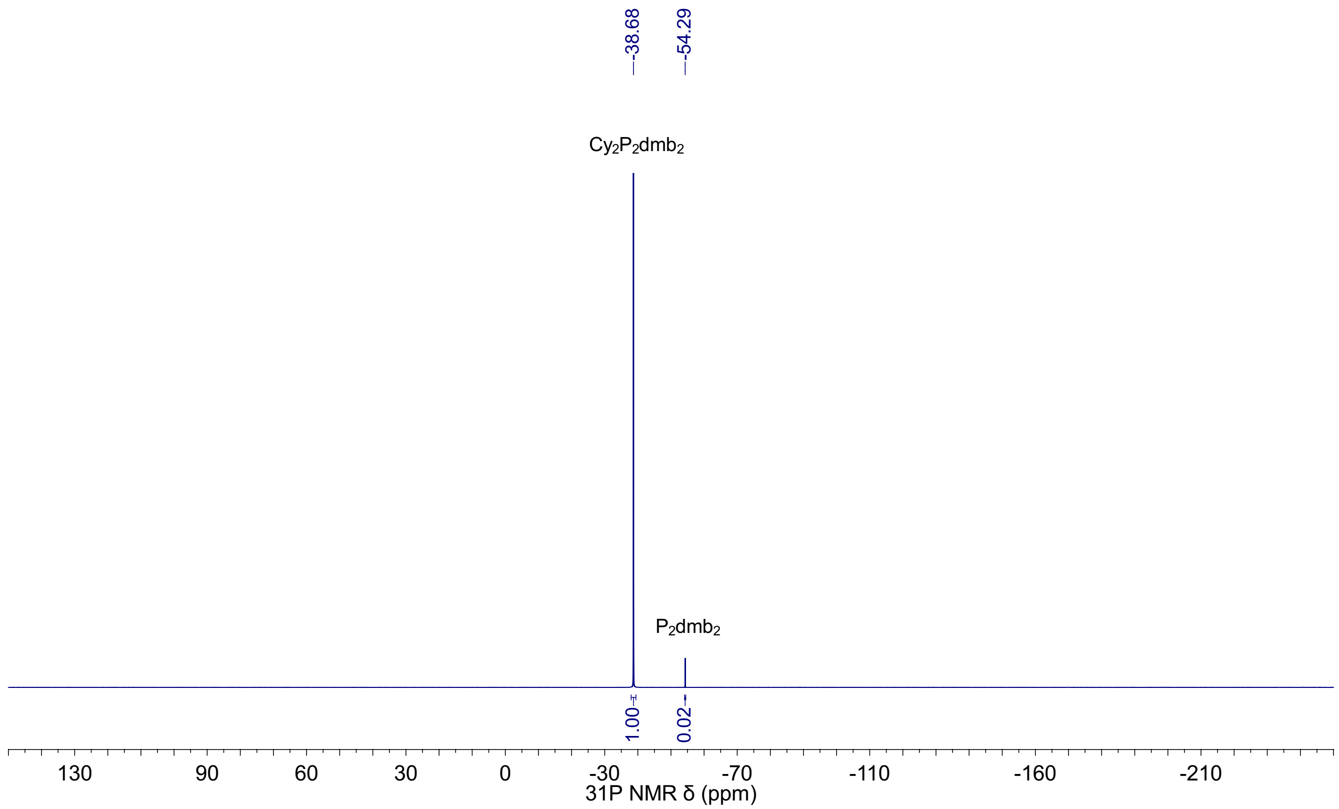

Figure S49 $^{31}P\{^{1}H\}$ NMR spectrum of Cy_2–DPC in C_6D_6 at 25 °C

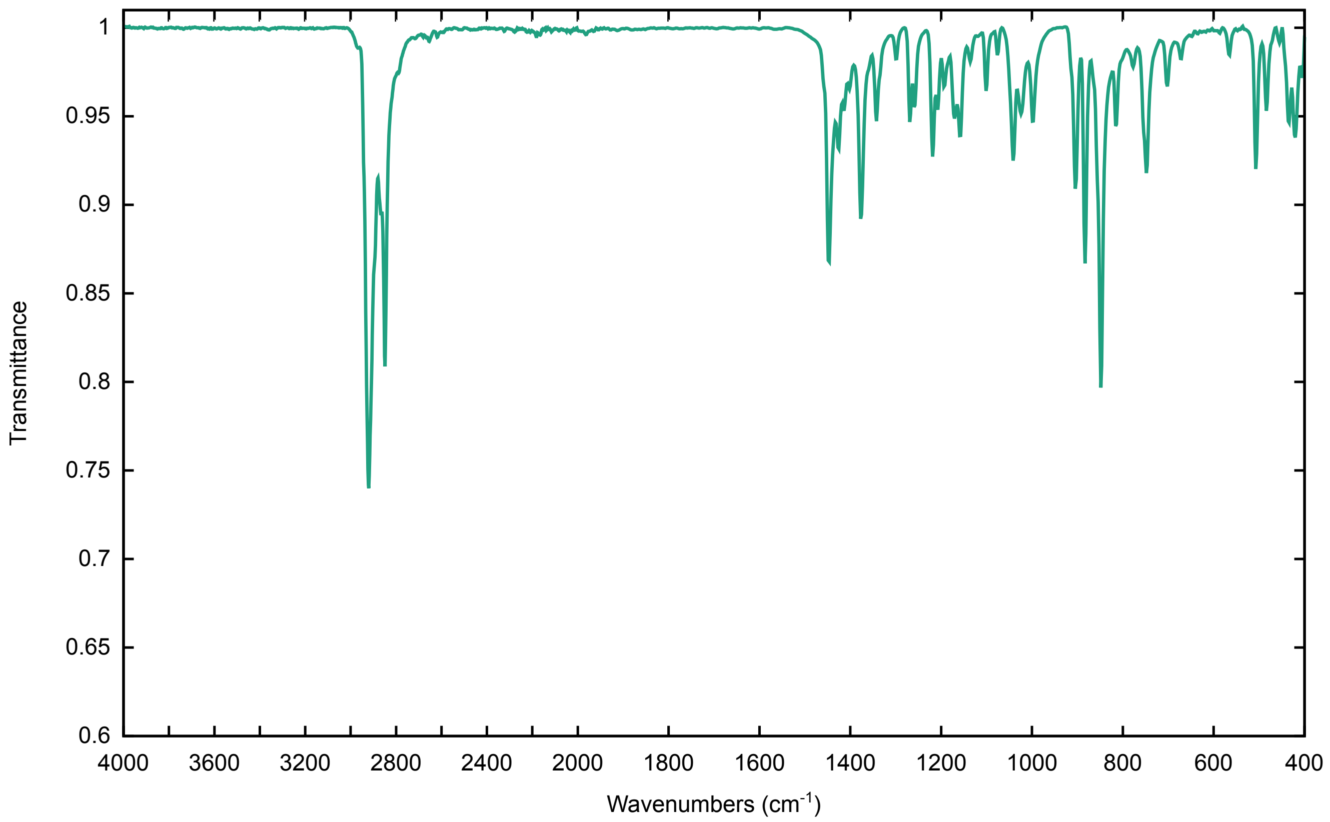

Figure S50 ATR-IR spectrum of Cy_2–DPC

14 (Cy$_2$–DPC)Ni(CH$_2$CH$_2$COO) (13)

14.1 Synthesis of (Cy$_2$–DPC)Ni(CH$_2$CH$_2$COO) (13)

Prep 1: A similar procedure to that used by Fischer et al. to prepare various nickelalactones by ligand exchange was used.[13] (TMEDA)Ni(CH$_2$CH$_2$COO) (38 mg, 0.154 mmol, 1 equiv) was slurried in 1 mL diethyl ether. The ligand, Cy$_2$–DPC (66 mg, 0.168 mg, 1.1 equiv), was dissolved in 6 mL diethyl ether and added to the (TMEDA)Ni(CH$_2$CH$_2$COO). The color of the slurry turned from greenish to yellow, but the mixture remained heterogeneous. After 5 hours of stirring at room temperature, the mixture was placed under vacuum and all volatiles were removed. Pentane (4 mL) was added and the resulting slurry was filtered. The product was washed with an additional 2 mL pentane, then dried under vacuum. A yellow powder (56 mg, 0.107 mmol, 69% yield if pure) was obtained. This product was analyzed by ^{31}P{^{1}H} NMR spectroscopy and proved to be contaminated with several impurities. Purification by crystallization was unsuccessful in obtaining a batch of spectroscopically clean material. However, crystallization by slow vapor diffusion of pentane into a toluene solution of this product yielded single crystals that allowed us to obtain the X-ray structure of **13**.

Prep 2: This procedure was adapted from the one used by Limbach et al. to prepare the nickelalactone of (R,R)-BenzP* from CO$_2$ and ethylene.[14] Inside the glovebox, Ni(COD)$_2$ (110 mg, 0.4 mmol, 1 equiv) and Cy$_2$–DPC (157 mg, 0.4 mmol, 1 equiv) were dissolved in 10 mL of THF and transferred to the glass liner of a 50 mL Parr pressure vessel. The Parr reactor was then assembled, sealed, and taken outside of the glovebox, where it was placed inside a heating mantle that was then filled with aluminum shot, and the entire setup was connected to a temperature controller. The reaction vessel was pressurized with ethylene (10 bar), then carbon dioxide (30 bar) at room temperature (total 40 bar). The temperature controller was set to 70 °C and the reaction mixture was heated overnight at this temperature. After 18 hours, the vessel was allowed to cool to room temperature for ca. 30 min, then it was placed in an ice-water bath for ca. 30 min. The Parr reactor was slowly vented, briefly purged with nitrogen, sealed, and brought back inside the glovebox. After opening the reactor, the dark reaction mixture was transferred to a vial and diluted with 10 mL THF. This solution was filtered through glass microfibre filter paper in order to remove

the black precipitate (likely nickel black). The resulting orange, homogeneous solution was placed under vacuum and concentrated to ca. 0.5 mL, then 20 mL pentane were added to precipitate the product. The mixture was then filtered, and the yellow precipitate washed with 2 x 3 mL pentane. After drying under vacuum, $(Cy_2\text{–DPC})Ni(CH_2CH_2COO)$ (97 mg, 0.19 mmol, 47% yield if pure) was obtained as a yellow powder. Although $^{31}P\{^1H\}$ NMR spectroscopy shows that $(Cy_2\text{–DPC})Ni(CH_2CH_2COO)$ is the major species, the product is not spectroscopically pure. The material obtained through this CO_2/ethylene route was used for catalytic testing as a starting nickel source (Table 1).

14.2 Characterization of $(Cy_2\text{–DPC})Ni(CH_2CH_2COO)$ (13)

^1H NMR (400 MHz, C_6D_6, 25 °C) δ: 3.7 – 0.6 (broad, unresolved signals) ppm.

$^{31}P\{^1H\}$ NMR (162 MHz, C_6D_6, 25 °C) δ: *Prep 1*: 21.55 (br d, J = 37.1 Hz), −12.85 (br d, J = 36.1 Hz) ppm; *Prep 2*: 21.50 (br d, J = 41.2 Hz), −12.88 (br d, J = 40.1 Hz) ppm.

ATR-IR: 2920, 2847, 1627 (vs, C=O), 1445, 1320 cm^{-1}.

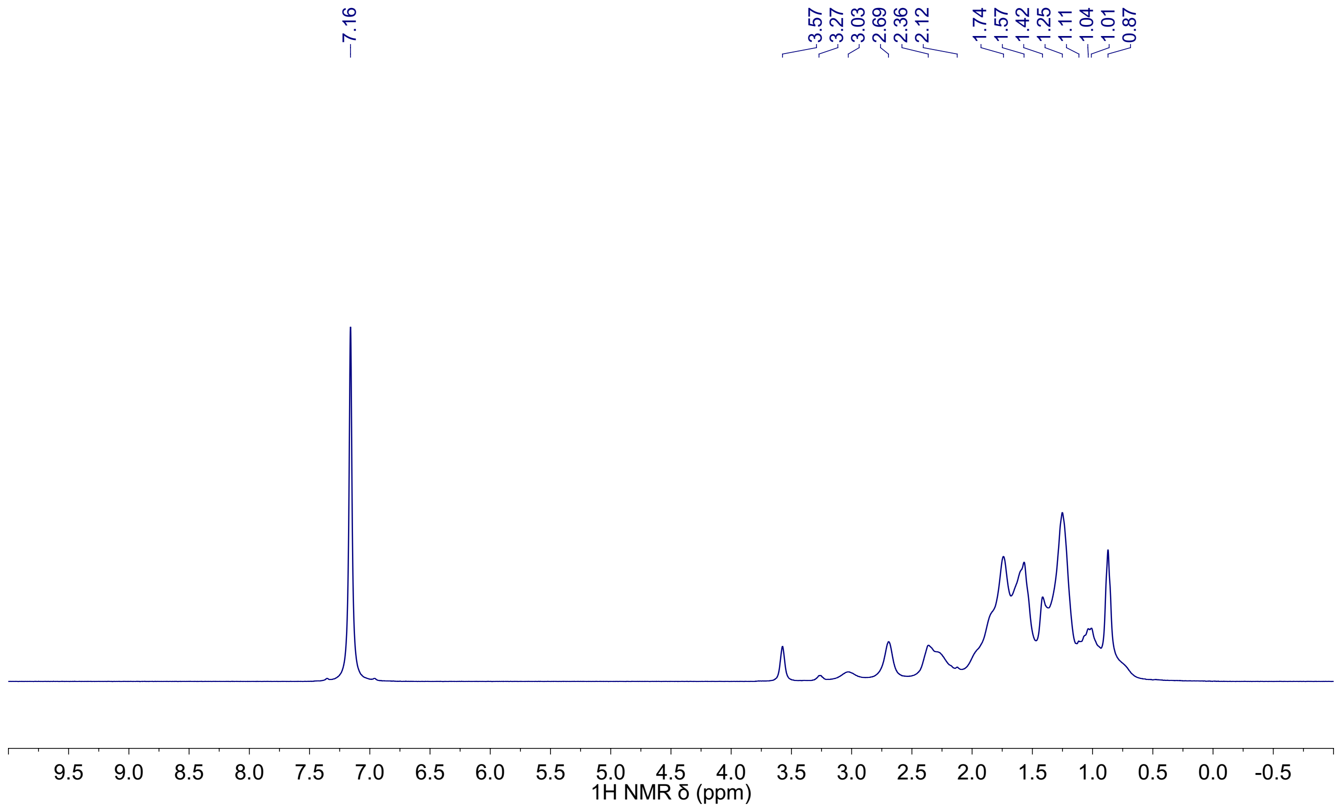

Figure S51 ^1H NMR spectrum of $(Cy_2\text{–DPC})Ni(CH_2CH_2COO)$ in C_6D_6 from *Prep 1* at 25 °C

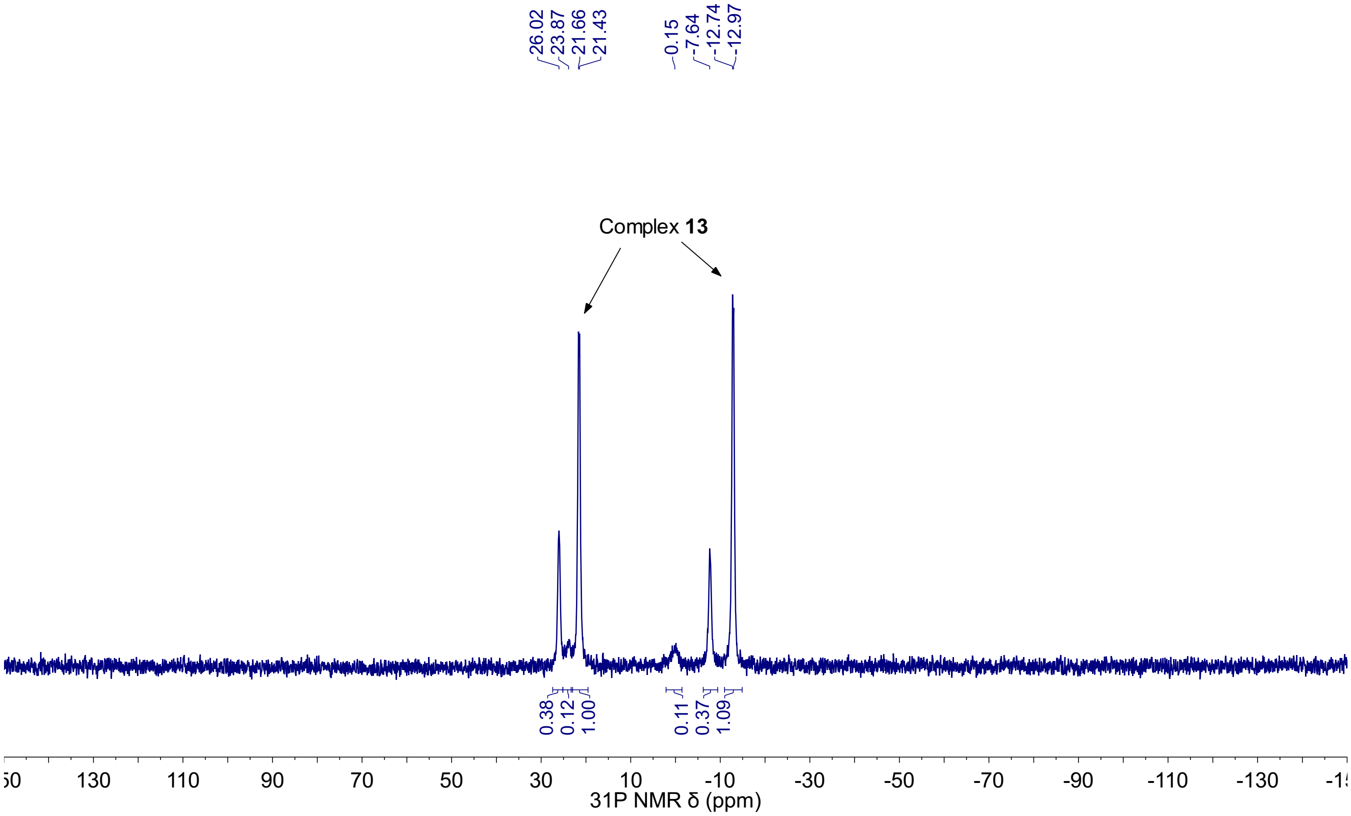

Figure S52 $^{31}P\{^{1}H\}$ NMR spectrum of (Cy$_2$–DPC)Ni(CH$_2$CH$_2$COO) in C_6D_6 from *Prep 1* at 25 °C

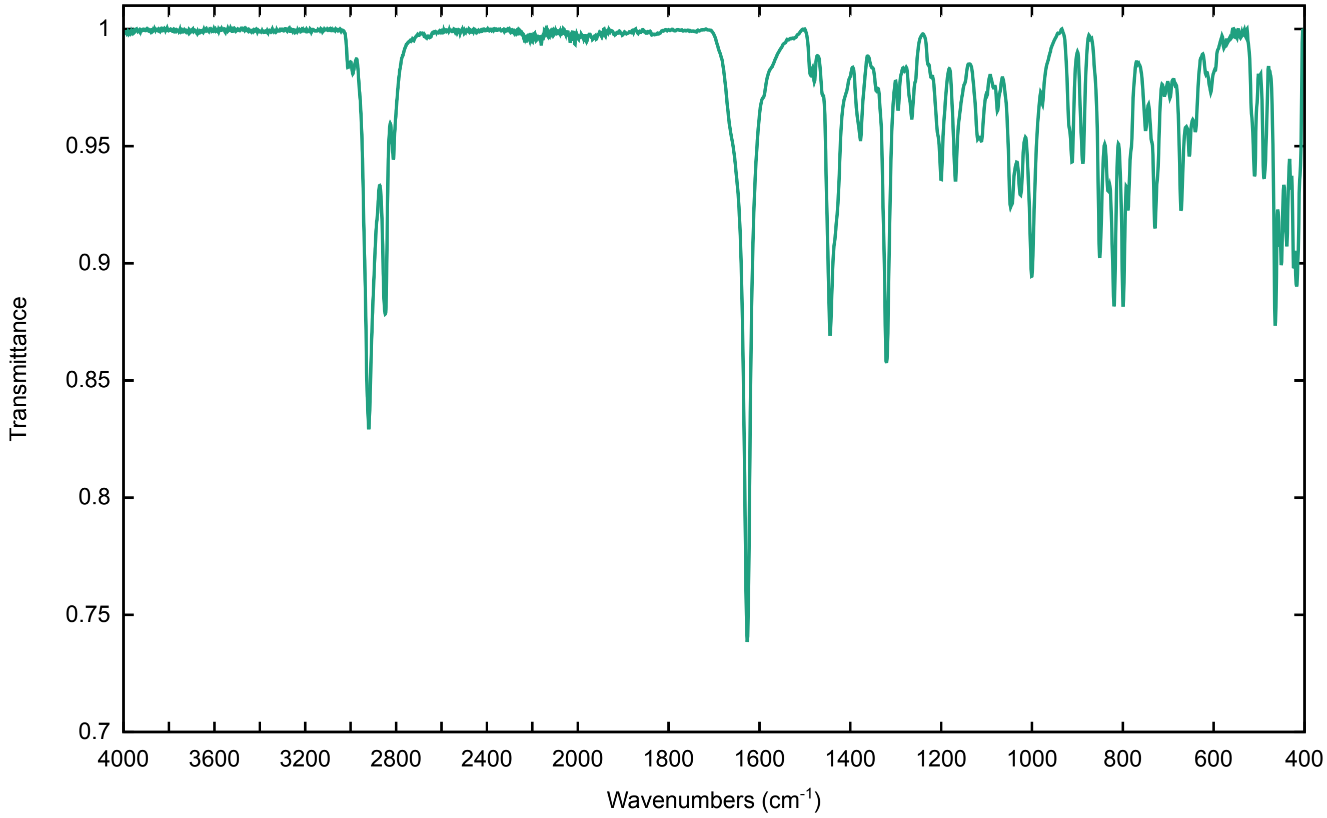

Figure S53 ATR-IR spectrum of (Cy$_2$–DPC)Ni(CH$_2$CH$_2$COO) from *Prep 1*

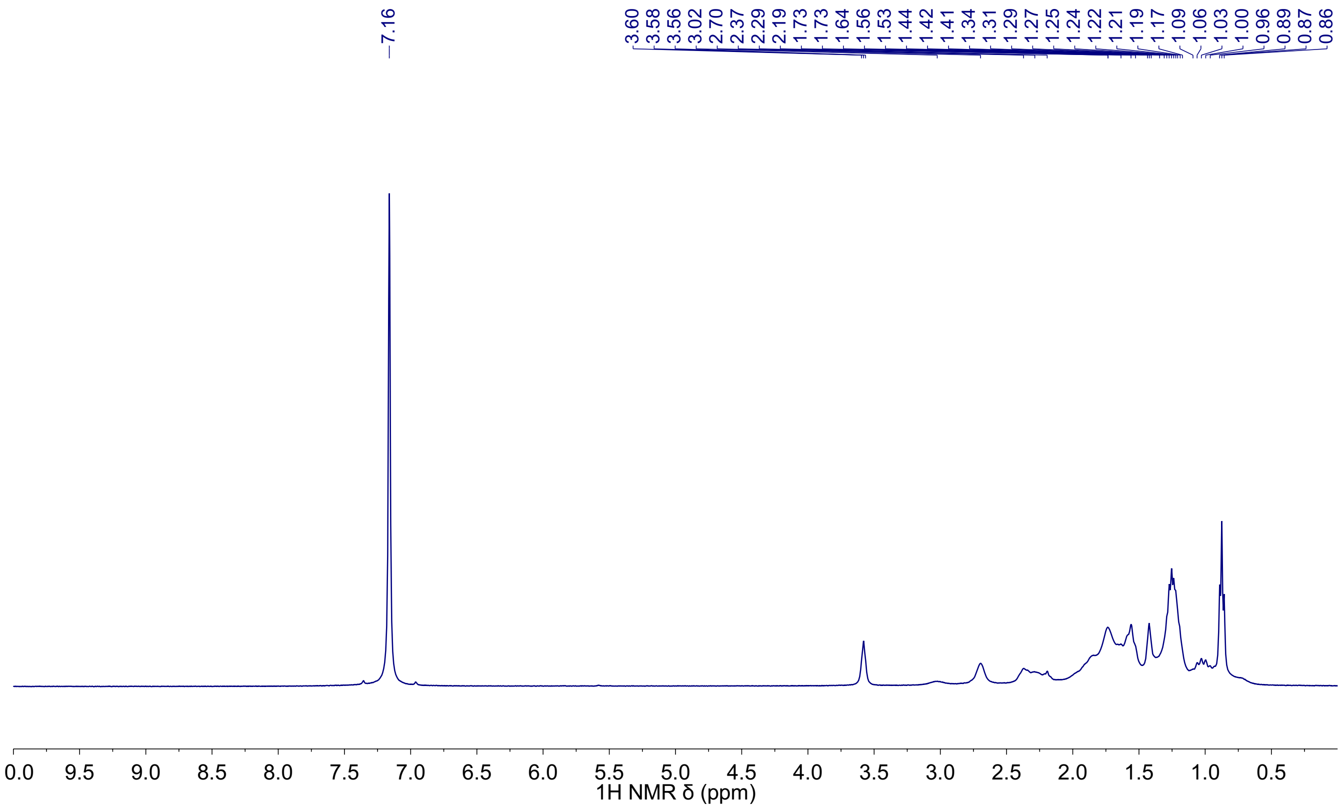

Figure S54 ^{1}H NMR spectrum of (Cy$_2$–DPC)Ni(CH$_2$CH$_2$COO) in C_6D_6 from *Prep 2* at 25 °C

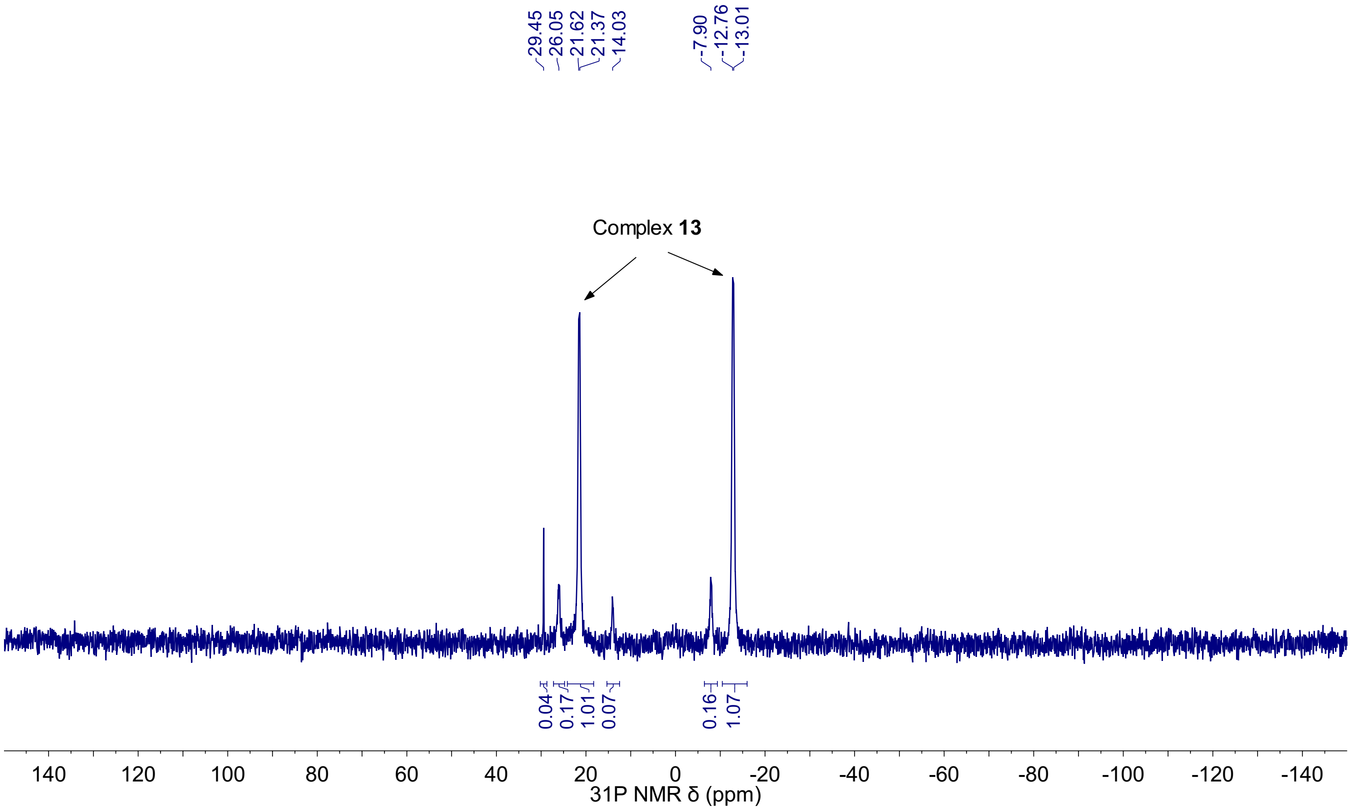

Figure S55 $^{31}P\{^{1}H\}$ NMR spectrum of (Cy$_2$–DPC)Ni(CH$_2$CH$_2$COO) in C_6D_6 from *Prep 2* at 25 °C

15 Experimental details for CO_2/C_2H_4 coupling catalytic tests

Method A (adapted from Vogt et al.[15]): Ni(COD)$_2$ (13.8 mg, 0.05 mmol, 1 equiv) and one equivalent of ligand were dissolved in 2 mL chlorobenzene. The resulting solution was added to a 10 mL vial, equipped with a small stirring bar, containing Zn (163.5 mg, 2.5 mmol, 50 equiv) and LiI (167 mg, 1.25 equiv, 25 equiv). To the solution was added triethylamine (0.35 mL, 2.5 mmol, 50 equiv), the vial was capped with a septum seal and the septum was perforated with a needle that was kept in place. The vial was placed in a Parr autoclave together with 3-5 other vials of other experiments, the autoclave was closed and heated to 50 °C by placing the autoclave in an oil bath kept at that temperature. Subsequently, the autoclave was pressurized first to 25 bar using ethylene, and subsequently further pressurized to 30 bar with carbon dioxide. The solution was stirred for 24 hours at this pressure and temperature. After 24 hours, the heating was turned off, and the reactor slowly vented over a 30 minute period by cracking open one of the needle valves. The reactor was opened and the samples removed from the autoclave. The reaction mixture (solution) typically had an orange-red color over a grey powder.

The vial was opened, and 20.0 mg lithium acetate dihydrate (LiOAc·2H_2O) was added, followed by ca. 1 mL of D_2O. The contents of the vial were vigorously shaken to dissolve all the salts in the D_2O layer. The solution was transferred to a centrifuge tube and centrifuged (5000 rpm) to precipitate all solids, and the D_2O layer was separated from the chlorobenzene layer. The D_2O layer was transferred to an NMR tube and analyzed by NMR spectroscopy. ^{1}H NMR and ^{13}C NMR spectra were recorded on a Bruker 400 Mz spectrometer. In the spectra, the only products that can be observed are acrylate (several multiplets between 6.1 and 5.6 ppm; 3H), D_2O (residual solvent signal and H_2O from the internal standard; 4.73 ppm), a quartet and a triplet for triethylamine (3.12 ppm and 1.22 ppm) and lithium acetate (1.86 ppm, methyl group, 3H). Significant vertical expansion of the spectrum shows some minor multiplets in the baseline. The amount of acrylate produced is determined by integrating the total area of the acrylate signals (Aacr) and the area of the methyl signal of the internal standard (ALA) and calculating the amount of acrylate from:

(mmol acrylate) = (Aacr) / (ALA) × ((20 mg LiOAc·2H_2O) / (102.01 mg / mmol)) × (3H / 3H)

The turnover number (TON) then follows from:

TON = (mmol acrylate) / ((13.8 mg Ni(cod)$_2$) / (275.08 mg / mmol))

Table S2 Catalytic acrylate production from CO_2 and ethylene using the procedure described in *Method A*

Ligand	TON (test #1)	TON (test #2)	TON (average)
Me_2–DPC (**5**)	n/a[a]	1.6	1
Me,Cy–DPC (**6**)	5.7	2.8	4
^{i}Bu,Cy–DPC (**7**)	10.1	9.2	10
Bn,Cy–DPC (**8**)	10.0	7.9	9
Bn,Ph–DPC (**9**)	9.8	14.7	12
Bn,Mes–DPC (**10**)	0.3	0.2	0
Cy_2–DPC (**12**)	10.9	12.5	12
dicyclohexylphosphinoethane (dcpe)	9.0	7.0	8
dicyclohexylphosphinopropane (dcpp)	19.2	16.6[b]	18
dicyclohexylphosphinobutane (dcpb)	6.2	6.0	6

[a] acrylate signals could not be reliably integrated; [b] no zinc.

Method B (adapted from Limbach et al.[14]): $Ni(COD)_2$ (20 mg, 0.07 mmol, 1 equiv) was weighed into the glass liner of a 50 mL Parr vessel. A stir bar and a solution of the ligand (0.077 mmol, 1.1 equiv) in 4 mL THF were added. Sodium 2-fluorophenoxide (469 mg, 3.5 mmol, 50 equiv) and Zn (229 mg, 3.5 mmol, 50 equiv) were added as solids to this solution, followed by 6 mL THF (total 10 mL THF). The liner was carefully placed inside the Parr vessel, which was then sealed and taken outside the glovebox. The vessel was placed inside a heating mantle that was then filled with aluminum shot, and the entire setup was connected to a temperature controller. Ethylene was introduced first, up to 10 bar, then CO_2 (10 bar) was added (total 20 bar). The temperature controller was set to 100 °C and the reaction heated overnight. After 20 hours, the reaction was cooled down for 1 hour, then put in an ice-water bath to help with cooling. The pressure was released carefully, then the reaction mixture was transferred to a 50 mL tube. The liner was washed with 5 mL D_2O, which was added to the tube with the reaction mixture. The internal standard, sodium 3-(trimethylsilyl)propionate-2,2,3,3-d_4 (25.8 mg, 0.15 mmol) in 2 mL D_2O was then added, then that vial washed with 1 mL D_2O and everything consolidated. Diethyl ether (20 mL) was added, the tube was closed, shaken, vented, shaken twice more, then allowed to settle so the layers separated. A syringe with a long needle was used to take a 2 mL aliquot of the aqueous layer. This was then filtered (to remove the Zn) and analyzed by ^{1}H NMR (64 scans). The internal standard integral was set to 9.0 (as there are 9 protons per internal standard), then the three acrylate protons were integrated separately, and the average of these integrals (A) was used as the ratio of acrylate to internal standard. The TON was obtained by multiplying the ratio A by the mmols of internal standard used, then divided by the mmols of Ni used.

16 Crystallographic details

Colorless needles of [Me–P_2(dmb)$_2$]I (**2**) were grown from THF. The compound crystallized in the monoclinic space group $P2_1/c$, with one [Me–P_2(dmb)$_2$]I and one THF in the asymmetric unit. The THF solvent molecule was disordered and was modeled over two positions. The ratio of the two components converged to 0.497(17):0.503(17).

Colorless plates of Me_2–DPC (**5**) were grown from toluene. The diphosphine crystallized in the monoclinic space group $P2_1/n$, with one molecule of Me_2–DPC in the asymmetric unit, no disorders and no crystallization solvent.

Colorless blocks of I_2·P_2(dmb)$_2$ (I_2·**1**) were grown from chloroform. The complex crystallized in the monoclinic space group $C2/c$, with one I_2·**1** and two molecules of $CHCl_3$ per asymmetric unit. The chloroform solvent molecules were disordered and modeled over two positions. The ratio of the two components converged to 0.65(4):0.35(4) for one molecule, and 0.58(4):0.42(4) for the other one. One of the iodine atoms was also disordered over two positions, with an occupancy ratio of 0.71(4):0.29(4).

Note: While the identity of this adduct (I_2·**1**) was established through X-ray crystallography, adequate NMR spectroscopic data could not be acquired for this compound as it is extremely insoluble in common organic solvents.

Gold needles of (Cy_2–DPC)Ni(CH_2CH_2COO) (**13**) were grown by slow vapor diffusion of pentane into a toluene solution of the complex. The compound crystallized in the orthorhombic space group $P2_12_12_1$, with one molecule of **13** in the asymmetric unit, no disorders and no crystallization solvent.

Table S3 Crystallographic details for [Me–P_2(dmb)$_2$]I (**2**) and Me_2–DPC (**5**)

	**[Me–P_2(dmb)$_2$]I (2)**	**Me_2–DPC (5)**
Reciprocal Net code / CCDC no.	D8_10140 / 1477899	D8_12021 / 1477900
Empirical formula, FW (g/mol)	$C_{17}H_{31}IOP_2$, 440.26	$C_{14}H_{26}P_2$, 256.29
Color / Morphology	Colorless / Needle	Colorless / Plate
Crystal size (mm^3)	$0.25 \times 0.05 \times 0.05$	$0.50 \times 0.30 \times 0.05$
Temperature (K)	100(2)	100(2)
Wavelength (Å)	1.54178	1.54178
Crystal system, Space group	Monoclinic, $P2_1/c$	Monoclinic, $P2_1/n$
Unit cell dimensions (Å, °)	a = 10.0682(2), α = 90	a = 8.4848(2) , α = 90
	b = 13.5159(2), β = 92.3520(10)	b = 12.9532(3) , β = 105.0400(10)
	c = 14.7514(2) , γ = 90	c = 14.0671(3), γ = 90
Volume (Å^3)	2005.69(6)	1493.09(6)
Z	4	4
Density (calc., g/cm^3)	1.458	1.140
Absorption coefficient (mm^{-1})	14.023	2.421
F(000)	896	560
Theta range for data collection (°)	4.395 to 65.824	4.717 to 69.789
Index ranges	$-11 \le h \le 11$, $-15 \le k \le 15$,	$-10 \le h \le 10$, $-15 \le k \le 15$,
	$-17 \le l \le 17$	$-16 \le l \le 17$
Reflections collected	37624	30850
Independent reflections, R_{int}	3398 (0.0572)	2805 (0.0375)
Completeness to θ_{max} (%)	93.8	100.0
Absorption correction	Semi-empirical from equivalents	Semi-empirical from equivalents
Max. and min. transmission	0.5407 and 0.1274	0.8885 and 0.3774
Refinement method	Full-matrix least-squares on F^2	Full-matrix least-squares on F^2
Data / restraints / parameters	3398 / 196 / 241	2805 / 8 / 175
Goodness-of-fit^a	1.078	1.074
Final R indices^b [$I > 2\sigma(I)$]	R_1 = 0.0395 , wR_2 = 0.1013	R_1 = 0.0381, wR_2 = 0.1018
R indices^b (all data)	R_1 = 0.0465, wR_2 = 0.1071	R_1 = 0.0421, wR_2 = 0.1067
Largest diff. peak and hole ($e \cdot$ Å^{-3})	1.850 and −0.900	0.805 and −0.224

^{a} GooF = $\left[\frac{\Sigma[w(F_o^2-F_c^2)^2]}{(n-p)}\right]^{\frac{1}{2}}$ ^{b} $R_1 = \frac{\Sigma||F_o|-|F_c||}{\Sigma|F_o|}$; $wR_2 = \left[\frac{\Sigma[w(F_o^2-F_c^2)^2]}{\Sigma[w(F_o^2)^2]}\right]^{\frac{1}{2}}$; $w = \frac{1}{\sigma^2(F_o^2)+(aP)^2+bP}$; $P = \frac{2F_c^2+\max(F_o^2,0)}{3}$

Table S4 Crystallographic details for $I_2 \cdot P_2(dmb)_2$ ($I_2 \cdot$**1**) and (Cy_2–DPC)Ni(CH_2CH_2COO) (**13**)

	**$I_2 \cdot P_2(dmb)_2$ ($I_2 \cdot 1$)**	**(Cy_2–DPC)Ni(CH_2CH_2COO) (13)**
Reciprocal Net code / CCDC no.	X8_12021 / 1477901	X8_15156 / 1477902
Empirical formula, FW (g/mol)	$C_{14}H_{22}Cl_6I_2P_2$, 718.75	$C_{27}H_{46}NiO_2P_2$, 523.29
Color / Morphology	Colorless / Block	Gold / Needle
Crystal size (mm^3)	$0.40 \times 0.10 \times 0.05$	$0.38 \times 0.13 \times 0.09$
Temperature (K)	100(2)	100(2)
Wavelength (Å)	0.71073	0.71073
Crystal system, Space group	Monoclinic, *C2/c*	Orthorhombic, $P2_12_12_1$
Unit cell dimensions (Å, °)	$a = 23.5655(16)$, $\alpha = 90$	$a = 9.6408(9)$, $\alpha = 90$
	$b = 10.2998(7)$, $\beta = 98.051(2)$	$b = 12.6202(12)$, $\beta = 90$
	$c = 20.9628(14)$, $\gamma = 90$	$c = 22.256(2)$, $\gamma = 90$
Volume (Å^3)	5037.9(6)	2707.8(4)
Z	8	4
Density (calc., g/cm^3)	1.895	1.284
Absorption coefficient (mm^{-1})	3.259	0.856
$F(000)$	2752	1128
Theta range for data collection (°)	1.745 to 30.508	1.830 to 27.550
Index ranges	$-33 \leq h \leq 33$, $-14 \leq k \leq 14$,	$-6 \leq h \leq 12$, $-16 \leq k \leq 11$,
	$-29 \leq l \leq 29$	$-17 \leq l \leq 28$
Reflections collected	53977	10744
Independent reflections, R_{int}	7692 (0.0337)	4926 (0.0392)
Completeness to θ_{max} (%)	100.0	98.5
Absorption correction	Semi-empirical from equivalents	Semi-empirical from equivalents
Max. and min. transmission	0.8540 and 0.3556	0.7456 and 0.6035
Refinement method	Full-matrix least-squares on F^2	Full-matrix least-squares on F^2
Data / restraints / parameters	7692 / 132 / 287	4926 / 0 / 293
Goodness-of-fit[a]	1.046	1.010
Final R indices[b] $[I > 2\sigma(I)]$	$R_1 = 0.0204$, $wR_2 = 0.0482$	$R_1 = 0.0477$, $wR_2 = 0.1111$
R indices[b] (all data)	$R_1 = 0.0248$, $wR_2 = 0.0503$	$R_1 = 0.0610$, $wR_2 = 0.1182$
Largest diff. peak and hole ($e \cdot$ Å^{-3})	1.451 and −0.572	1.163 and −0.785

[a] GooF = $\left[\frac{\Sigma[w(F_o^2-F_c^2)^2]}{(n-p)}\right]^{\frac{1}{2}}$ [b] $R_1 = \frac{\Sigma||F_o|-|F_c||}{\Sigma|F_o|}$; $wR_2 = \left[\frac{\Sigma[w(F_o^2-F_c^2)^2]}{\Sigma[w(F_o^2)^2]}\right]^{\frac{1}{2}}$; $w = \frac{1}{\sigma^2(F_o^2)+(aP)^2+bP}$; $P = \frac{2F_c^2+\max(F_o^2,0)}{3}$

References

[1] C. L. Perrin and T. J. Dwyer, *Chem. Rev.*, 1990, **90**, 935–967.

[2] H. Bircher, B. R. Bender and W. Vonphilipsborn, *Magn. Reson. Chem.*, 1993, **31**, 293–298.

[3] E. R. Civitello, P. S. Dragovich, T. B. Karpishin, S. G. Novick, G. Bierach, J. F. O'Connell and T. D. Westmoreland, *Inorg. Chem.*, 1993, **32**, 237–241.

[4] A. Gogoll, J. Örnebro, H. Grennberg and J.-E. Bäckvall, *J. Am. Chem. Soc.*, 1994, **116**, 3631–3632.

[5] Bruker, *SAINT, Bruker-AXS Inc., Madison, Wisconsin, USA*, 2010.

[6] G. M. Sheldrick, *SADABS, University of Göttingen: Germany*, 1996.

[7] G. M. Sheldrick, *Acta Cryst.*, 1990, **A46**, 467–473.

[8] G. M. Sheldrick, *Acta Cryst.*, 2015, **A71**, 3–8.

[9] G. M. Sheldrick, *Acta Cryst.*, 2008, **A64**, 112–122.

[10] P. Müller, R. Herbst-Irmer, A. L. Spek, T. R. Schneider and M. R. Sawaya, *Crystal Structure Refinement: A Crystallographers Guide to SHELXL*, Oxford University Press: Oxford, 2006.

[11] P. Müller, *Cryst. Rev.*, 2009, **15**, 57–83.

[12] D. Tofan and C. C. Cummins, *Angew. Chem., Int. Ed.*, 2010, **49**, 7516–7518.

[13] R. Fischer, J. Langer, A. Malassa, D. Walther, H. Görls and G. Vaughan, *Chem. Commun.*, 2006, 2510–2512.

[14] N. Huguet, I. Jevtovikj, A. Gordillo, M. L. Lejkowski, R. Lindner, M. Bru, A. Y. Khalimon, F. Rominger, S. A. Schunk, P. Hofmann and M. Limbach, *Chem. Eur. J.*, 2014, **20**, 16858–16862.

[15] C. Hendriksen, E. A. Pidko, G. Yang, B. Schäffner and D. Vogt, *Chem. Eur. J.*, 2014, **20**, 12037–12040.
